# Under pressure: Clinical management of venom-induced compartment syndrome in snakebite–A scoping review of the global literature

**DOI:** 10.1371/journal.pntd.0014536

**Published:** 2026-07-31

**Authors:** Jonathan Steinhorst, John H. Amuasi, Paul M. N. Werker, Abdulrazaq G. Habib, Eric J. Lavonas, Benno Kreuels, David G. Lalloo, Ymkje Stienstra

**Affiliations:** 1 Department of Internal Medicine/ Infectious Diseases, University of Groningen and University Medical Center Groningen, Groningen, The Netherlands; 2 Centre for Snakebite Research and Interventions, Liverpool School of Tropical Medicine, Liverpool, United Kingdom; 3 Kumasi Center for Collaborative Research in Tropical Medicine, Kwame Nkrumah University of Science and Technology, Kumasi, Ghana; 4 Department of Plastic Surgery, University of Groningen and University Medical Center Groningen, Groningen, The Netherlands; 5 Department of Medicine, Bayero University, Kano, Nigeria; 6 Rocky Mountain Poison and Drug Safety, Denver Health, Denver, Colorado, United States of America; 7 Department of Emergency Medicine, University of Colorado School of Medicine, Aurora, Colorado, United States of America; 8 Research Group Neglected Diseases and Envenoming, Bernhard Nocht Institute for Tropical Medicine, Hamburg, Germany; 9 Department of Internal Medicine, University Medical Centre Hamburg-Eppendorf, Hamburg, Germany; Fundação de Medicina Tropical Doutor Heitor Vieira Dourado: Fundacao de Medicina Tropical Doutor Heitor Vieira Dourado, BRAZIL

## Abstract

**Background:**

Venom-induced compartment syndrome (VICS) is a rare but severe complication of snakebite which poses unique challenges in diagnosis and treatment. Published literature largely focuses on VICS in the North American context, while information on clinical presentations, diagnostic and treatment approaches, clinical outcomes and associated challenges from the world’s most snakebite endemic regions remain fragmented and scarce.

**Methods:**

A scoping review using PRIMSA-ScR methodology of the global literature on VICS was performed by searching the PubMed, Embase, and Cochrane databases. Literature was divided into 1) Case reports on VICS and 2) General literature. Clinical data from eligible case reports was extracted to describe management of VICS and associated challenges. Available evidence on diagnostic and treatment strategies was extracted from the general literature.

**Results:**

Of 115 cases of VICS analyzed, most were from Europe (40%); Only 13% and 6% were reported from South-East Asia and Africa respectively. Viperids caused 73% of bites and upper extremities were most frequently affected (63%). Compartment pressure was measured in 38% of patients. Compartment pressure was more commonly measured in the 12% of non-surgically treated patients, none of whom developed ischaemic contracture or required amputation. Coagulopathy was the most common systemic toxicity, present in 40% of patients at admission. Results from eight animal studies support the use of antivenom for treating VICS. Analysis of 17 human observational studies suggests an overdiagnosis of VICS using clinical symptoms alone, highlighting the need to investigate diagnostic tools, such as ultrasound, for diagnosing compartment swelling.

**Conclusion:**

Antivenom as first-line treatment for VICS is supported by animal and human observational studies. However, additional data is needed to inform decision-making on when fasciotomy is indicated as a rescue therapy. Improved evidence generation will depend on the collection of high-quality clinical and diagnostic observational data from patients treated with antivenom in snakebite-endemic regions.

## Author summary

Venom-induced compartment syndrome (VICS) occurs when swelling caused by snake venoms inside a muscle compartment compresses capillary vessels leading to ischaemia, muscle necrosis, and potentially limb loss. VICS is a rare complication of snakebite and data describing patient characteristics, clinical management strategies and their associated outcomes are scarce and fragmented. In this global scoping review, 115 case reports detailing the clinical management and outcomes of patients with VICS alongside evidence from human observational studies and animal experiments are analyzed. Reporting of cases was skewed towards high-income regions, with few publications from Africa and South-East Asia where the global snakebite burden is greatest. Viperid bites were the most frequent cause of VICS (73%) and 40% of patients had a coagulopathy upon admission. Non-surgical treatment with antivenom alone was more frequently associated with serial compartment pressure monitoring than treatment involving surgical decompression. Human observational studies show that utilizing diagnostic tools can reduce the overdiagnosis of VICS. Animal experimental data supports the use of antivenom to reduce compartment pressures. Developing and facilitating access to diagnostic tools for VICS could reduce the number of decompression surgeries and combined with improved case-reporting in scientific literature, could support the development of evidence-informed diagnostic and treatment algorithms.

## Introduction

Snakebite is a neglected tropical disease affecting an estimated 5.4 million people globally per year, of which 2.7 million bites likely result in envenoming [1]. Envenoming can result in a myriad of systemic and local symptoms and complications, depending on the involved snake species, the quantity of venom injected and the anatomic location of the bite [2–5]. Snake venoms, particularly those of viperids and some elapids, contain a host of enzymes implicated in tissue destruction [6]. In extreme cases, progression to venom-induced compartment syndrome (VICS), a state of excessive swelling inside a muscle compartment which compromises local tissue perfusion, causing ischaemia and muscle necrosis, can occur [7]. VICS constitutes a clinical emergency which can cause loss of limb function [8] or even the extremity itself [9–11].

The incidence of VICS following snakebite is generally considered to be low [12,13]. For instance, only 1% of 1604 snakebite patients reported to the North American Snakebite Registry were suspected of having VICS [14]. However, some authors report a diagnosis in as many as 17–51% of snakebite cases [3,15–17]. Most evidence primarily originates from studies performed in the United States of America (USA), Europe, the Middle East and a select number of countries in Asia. There is a paucity of data from African nations and countries in Asia where cytotoxic snakebite is common [18]. Estimating the incidence of VICS is further complicated by the lack of uniform diagnostic criteria. Adding to this difficulty, envenoming can cause subcutaneous tissue inflammation which mimics the signs and symptoms of compartment syndrome [10,12,19,20].

While there is little debate that the only definitive management of compartment syndrome in general trauma victims is decompression through fasciotomy [21,22], this approach has been questioned in VICS [12,23–29]. Antivenom therapy may reduce compartment pressures and sometimes obviate the need for fasciotomy [24,25,28,30]. Fasciotomy is a very invasive procedure which can cause inadvertent damage to nerves and blood vessels, the exposure of underlying tissue to infection, functional limitations due to scarring, skin graft contracture, and life-threatening haemorrhage in the envenomed patient [21,26,27,31]. Conversely, delayed treatment of compartment syndrome can also cause permanent loss of limb function. Deciding on a safe and effective treatment strategy for VICS in the face of diagnostic uncertainty can pose a major challenge for clinicians.

Questions about the appropriate diagnosis and treatment of VICS that remain unanswered are 1) how to accurately identify compartment syndrome in a patient who may just have subcutaneous swelling, 2) the effectiveness of antivenom and other non-invasive methods in reducing local tissue swelling, 3) the optimal timing and indication for fasciotomy, 4) and the requirements for pre- and post-operative adjunctive care in the envenomed patient. To date, two literature reviews [23,32], a published evidence-based expert recommendation [12] and a recently published scoping review [7] have sought to address these questions. However, except one general review on the topic [32], all of the abovementioned studies are focused on North America, which differs considerably from the world’s regions with the highest morbidity and mortality burden of snakebite envenoming in terms of endemic snake species and their venom profiles, patient demographics and the availability of diagnostic and treatment resources.

This scoping review was performed to provide clinicians with an overview of the different management strategies pursued globally for VICS and to highlight the challenges and outcomes associated with these. The available evidence underpinning different diagnostic and management strategies as well as information on their respective outcomes will be presented and discussed.

## Methods

### Rationale for scoping review

Heterogeneity in study designs, study subjects, reported variables and the predominance of lower-level evidence preclude efforts to synthesize existing knowledge on VICS in the form of a meta-analysis as in a systematic review. A scoping review format was selected instead. Scoping reviews provide a methodological framework for synthesizing evidence from a variety of study designs and sources. Their objective is to summarize existing research and knowledge on a topic as a basis for formulating future research questions. The methodology as outlined in the JBI Manual for Evidence Synthesis [33] was used as guidance during the literature review and the PRISMA-ScR checklist (S1 File) was followed while writing the report [34]. We first present data from case reports detailing the management of patients with VICS. Next, we summarized evidence from human observational studies, animal experiments and expert commentaries to discuss the current state of evidence on the management of VICS and to relate this evidence to the management challenges identified from case reports. This scoping review was pre-registered with the Center for Open Science, where the study protocol is publicly accessible [35].

### Search strategy and literature screening

A literature search was conducted in PubMed (NLM), Embase (Elsevier), and the Cochrane databases with a search string containing the keywords ‘snakebite’ and ‘compartment syndrome’ (S2 File). A professional librarian from the University Medical Center Groningen assisted in developing the PubMed search string and its translation for use in the Embase and Cochrane databases. The search included all literature published before October 5^th^, 2025, without language or time restrictions. Records were automatically de-duplicated in Endnote X9 [36] and Rayyan [37]. Subsequently, title and abstract screening was performed in Rayyan by two authors, JS and YS. Any remaining duplicates were removed manually during the screening process. Articles for which decisions between authors conflicted were included in full-text screening. Differences were resolved by consensus, with consultation of a third reviewer if required. Reference lists of included articles were manually searched for additional literature meeting the inclusion criteria. Articles published in languages other than English were translated by JS and YS (German, Dutch, French) or by native speakers in the respective language.

### Inclusion and exclusion criteria

Two sets of inclusion criteria for literature were formulated: the first for case reports and the second for all observational studies, animal experiments and commentaries, hereafter referred to collectively as ‘General literature’. The minimal requirements for inclusion of case reports were a reported diagnosis of VICS of the bitten extremity, mention of the affected body part, the treatment performed, and the clinical outcome. A diagnosis of VICS based on clinical grounds alone, i.e., without elevated compartment pressure measurements, was deemed sufficient for inclusion. Where compartment pressure was measured, author-reported pressure thresholds were maintained for the diagnosis of VICS. Patients treated with fasciotomy for reasons other than increased compartment pressure (e.g., necrotizing fasciitis) were excluded. Individual case descriptions from observational studies, conference abstracts and commentaries meeting the abovementioned inclusion criteria were included in the case-based analysis. Observational studies, animal experiments and commentaries were included if they explicitly reported on the management of VICS.

### Data extraction and interpretation

Two data extraction charts for case reports and general literature were made using Microsoft Excel. Data extracted from case reports included the country and World Health Organization (WHO) region [38] where the snakebite occurred, patient demographics, the context of the bite (snake family/genus/species; wild or captive snake), the anatomic location of the bite, clinical symptoms, diagnostic tools used for diagnosing VICS, results of coagulation tests, non-surgical and surgical treatments performed and the clinical outcomes achieved. Reliability of snake species identification was assumed when an exact genus and species name was reported. Bites incurred by snakes kept privately and at zoos were recorded as captive snake bites. Where available, the time intervals between the snakebite and hospital presentation, diagnosis of VICS, antivenom administration, and surgical treatment were recorded.

The following coagulation test results at admission or pre-operatively were noted: 20-minute whole blood clotting test (20WBCT), Activated Partial Thromboplastin Time (APTT), Prothrombin time (PT); International Normalized Ratio (INR) and platelet counts (Plt). In individual cases where reference values were missing, reference values from one of the included case reports [39] were used to classify patients as coagulopathic or thrombocytopenic. Author-reported coagulopathies without recording of coagulation tests were counted as coagulopathies. Details about antivenom administration, surgical interventions, the use of medications and blood products, intubation/intensive care and non-surgical treatments of the affected limb were extracted. Depending on the detail of reporting, clinical outcomes were categorized as ‘Survival/Deceased’, ‘Limb salvage’ (i.e., preservation of the limb could be inferred), ‘No sequelae’ (i.e., the status of full functional recovery/ no lasting disabilities was explicitly reported) or ‘Sequelae’ (irreversible functional or sensory impairments, including amputations). Scarring was not categorized as a sequela, as all patients treated surgically invariably have scars, which may be disfiguring but do not necessarily cause functional impairment. Complications during treatment (haemorrhage, new-onset coagulopathy, systemic organ failure, adverse reactions to antivenom, and wound/surgical site infections) were recorded. The day of discharge, if available, was noted as the last verifiable follow-up time point unless otherwise specified.

Data extracted from human observational studies from the rubric of general literature included 1) the proportion of patients diagnosed with VICS, 2) diagnostic methods used, 3) the proportion of patients treated with antivenom and/or fasciotomy, 4) intra-operative findings for patients treated with fasciotomy, 5) complications, and 6) investigational findings regarding diagnostic procedures and/or treatments performed for patients with suspected VICS. For animal studies, the species and number of animals, snake species of venom used, interventions tested, and experimental outcomes were extracted. Key recommendations for the diagnosis and treatment of VICS were summarized from included guidelines.

### Data analysis

Demographic and clinical variables extracted from case reports were inputted into IBM SPSS statistics version 30 to generate summary statistics. Graphs were made using RStudio Version 2025.09.1 + 401 [40].

## Results

### Case reports

**Fig 1:**
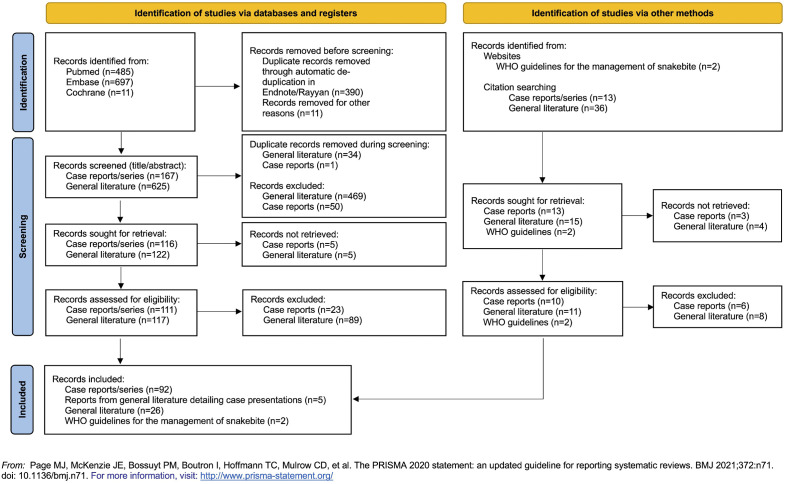
PRISMA-ScR flow diagram.

A total of 115 cases [19, 28–30, 39, 41–132] were included for analysis (S3 File). These cases were extracted from 92 case reports/ case series and 5 reports [29,54,65,71,103] from human observational studies (Fig 1). The largest number of compartment syndrome cases (n = 40) were reported from Europe, of which 40% (n = 16) involved bites by captive snakes (Table 1). These 16 cases constituted 84% of the reported captive snakebite VICS cases. Nearly three quarters of all reported cases of VICS occurred in male patients; 27% of patients were aged 10 years or younger. Most cases involved bites by *Viperidae* (73%). The fingers and hands were the most frequently reported bite sites, with 63% (n = 72) of all bites affecting the upper extremity. Bites from captive snakes were incurred exclusively on upper extremities and except for two cases, by males only. Most bites by captive snakes (n = 16; 84%) were reported from Europe. Patients arrived at the hospital between 15 minutes and seven days after the bite.

**Table 1:** Demographics of patients (n = 115) with reported venom-induced compartment syndrome.

	No Fasciotomy (n = 14; 100%) Median (IQR) or Column (%)	Fasciotomy ^1^ (n = 101; 100%) Median (IQR) or Column (%)	Total (n = 115; 100%) Median (IQR) or Column (%)
**Age in years** (Median; IQR) ^2^	12 (9–40)	22 (10–38)	21 (10–39)
**Sex** ^2^			
Female	5 (36)	25 (25)	30 (26)
**WHO Region**			
Europe	5 (36)	35 (35)	40 (35)
Americas	6 (43)	25 (25)	31 (27)
South-East Asia	1 (7)	14 (14)	15 (13)
Eastern Mediterranean	1 (7)	10 (10)	11 (10)
Western Pacific	0	11 (11)	11 (10)
Africa	1 (7)	6 (6)	7 (6)
**Captive snake bite**	2	17	19 (17)
**Snake family/genera implicated in bite** ^3^			
*Viperidae* (total)	10 (71)	74 (73)	84 (73)
*Vipera*	2 (14)	14 (14)	16 (14)
*Bothrops*	1 (7)	12 (12)	13 (11)
*Crotalus*	4 (29)	11 (11)	15 (13)
*Bitis*	2 (14)	6 (6)	8 (7)
*Agkistrodon*	1 (7)	6 (6)	7 (6)
Other *Viperidae*	0	25 (25)	25 (22)
*Elapidae* (total)	0	4 (4)	4 (3)
*Naja*	0	2 (2)	2 (2)
*Dendroaspis*	0	1 (1)	1 (1)
*Pseudoechis*	0	1 (1)	1 (1)
Unknown	4 (29)	23 (23)	27 (23)
**Anatomic locations of snakebites**			
Finger	2 (14)	33 (33)	35 (30)
Hand	2 (14)	22 (22)	24 (21)
Leg	2 (14)	16 (16)	18 (16)
Foot	4 (29)	12 (12)	16 (14)
Arm	2 (14)	11 (11)	13 (11)
Ankle	2 (14)	6 (6)	8 (7)
Toe	0	1 (1)	1 (1)
**Time bite-hospital presentation** ^2^			
(Median; IQR)	3.5h (1–4h)	2h (1–8h)	2h (1–8h)

^1^Includes skin incisions for tension release

^2^Missing data for n number of cases: Age in years (n = 3), Sex (n = 4), Time-bite hospital (n = 44)

^3^For *Viperidae*, genera presented if frequency n ≥ 5

### Case reports: Clinical symptoms and diagnosis of VICS

**Fig 2:**
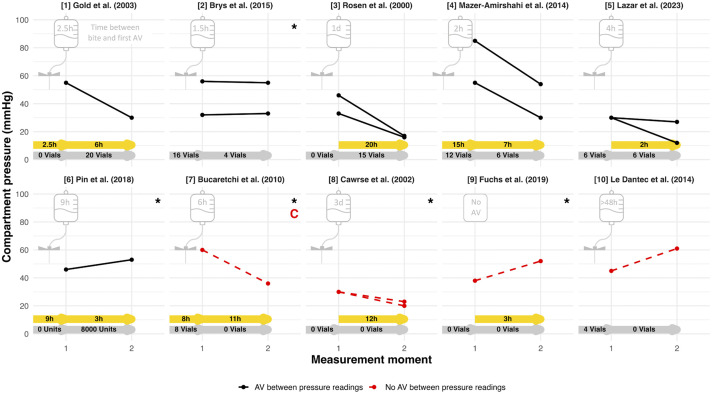
Repeated compartment pressure measurements in limb compartments of patients treated with antivenom (n = 6) and without antivenom (n = 4) between the first two pressure readings. Yellow arrows denote the time intervals between the bite and measurement moment 1 as well as measurement moment 1 and measurement moment 2. Grey arrows denote the amount of antivenom administered before and between the two pressure measurements. Compartment pressure was measured in multiple limb compartments in five patients. Patient 8 received antivenom after the second measurement moment. Patient 9 was the only patient who did not receive antivenom during the entire course of treatment. AV is antivenom, h is hours, d is day, the asterisks highlight patients treated with fasciotomy after the second pressure measurement, and C denotes that a complication was reported following treatment.

Swelling and pain were the most frequently reported symptoms (Table 2). Compartment pressure was measured in 38% (n = 44) of patients. By WHO region, compartment pressure was most frequently measured in patients from the Americas (n = 21, 48%), followed by Europe (n = 11, 25%). Serial compartment pressure measurements were reported for eleven patients, ten of whom were managed non-surgically between the two readings (Fig 2). Other diagnostic modalities employed included limb circumference measurements (n = 16), ultrasound (n = 3) and MRI (n = 2). Forty-eight patients (42%) had a coagulopathy at some stage during treatment.

**Table 2:** Diagnostic methods used and findings in patients (n = 115) with reported venom-induced compartment syndrome.

	No Fasciotomy (n = 14; 12%) Median (IQR) or Column (%)	Fasciotomy (n = 101; 88%) Median (IQR) or Column (%)	Total (n = 115; 100%) Median (IQR) or Column (%)
**Local clinical symptoms on presentation**			
Swelling	14 (100)	90 (89)	104 (90)
Pain	13 (93)	69 (68)	82 (71)
Paraesthesia/ hypoesthesia	5 (36)	39 (39)	44 (38)
Pain on passive stretch	9 (64)	25 (25)	34 (30)
Decreased/ absent arterial pulsations	4 (29)	23 (23)	27 (24)
Muscular paralysis	0	15 (15)	15 (13)
**Compartment pressure measured**	7 (50)	37 (37)	44 (38)
**Anatomic region of compartment pressure measurement** ^1,2^			
Hand	2	9	11
Arm	1	4	5
Leg	3	12	15
Foot	1	1	2
**Repeated measurement of compartment pressure**	5	6	11
**Maximum compartment pressure (mmHg) reported** ^1^			
< 30	0	2	2
30-40	2	5	7
41-60	3	13	16
61-80	1	7	8
> 81	1	4	5
**Time elapsed between snakebite and diagnosis of compartment syndrome** ^1^			
Median (IQR)	1h (0–12h)	6h (0–11h)	5h 30min (0–11h 15min)
**Haemorrhage at time of hospital presentation**	2 (14)	20 (20)	22 (19)
**Coagulopathy at time of hospital presentation** ^1, 3^			
Yes	6 (43)	40 (40)	46 (40)
No	7 (50)	24 (24)	31 (27)
**Thrombocytopenia at admission/ pre-operatively** ^1, 3^			
Yes	4 (29)	17 (17)	21 (18)
No	3 (21)	12 (12)	15 (13)
**Platelet counts (per mm**^**3**^**) at time of hospital presentation** ^1, 3^			
< 30 000	3	6	9 (8)
30 000–60 000	0	4	4 (3)
> 60 000	4	16	20 (17)

^1^Missing data for the following number of cases: Time elapsed in hours between snakebite and diagnosis of compartment syndrome (n=55), compartment pressure measurements (n=6), anatomic region of compartment pressure measurements (n = 14), tests of coagulation/coagulopathy (n=38), platelet counts (n=82), thrombocytopenia (n=79)

^2^In some patients, multiple compartments were measured

^3^If reference values were not reported, reference values from Bucaretchi et al. [39] were used for defining coagulopathy and thrombocytopenia: International normalized ratio (INR) <1.20, Activated Partial Thromboplastin Time (APTT/PTT) <28s, Prothrombin time (PT) <14.3s, platelets (plt) 150–400 x 10^3^/mm^3^ or 150–400 x 10^9^/L. Author-reported coagulopathy and thrombocytopenia are included.

### Case reports: Treatment of VICS

One-hundred-one patients were treated with fasciotomy and 14 were treated non-surgically (Table 3). The majority (n = 11) of the latter were treated in Europe (n = 5) or the Americas (n = 5 in the USA; n = 1 in Brazil). Compartment pressure was measured more frequently in the non-surgically managed group than in the surgically managed group (50 vs. 37%). Among patients undergoing compartment pressure measurements, serial measurements were more often performed among patients managed non-surgically (71% vs 16%).

**Table 3:** Treatments provided to patients (n = 115) with reported venom-induced compartment syndrome.

	No Fasciotomy (n = 14; 12%) Median (IQR) or Column (%)	Fasciotomy (n = 101; 88%) Median (IQR) or Column (%)	Total (n = 115; 100%) Median (IQR) or Column (%)
**Antivenom**			
At any time during course of treatment	13 (93)	75 (74)	88 (77)
**Time bite-antivenom** ^1^			
Median	3h 15min	6h	5h 30min
IQR	(1h–4h 30min)	(2h 40min–13h)	(2h 9min–12h)
**Blood products** ^2^	5 (36)	20 (20)	25 (22)
**Surgical procedures (other than fasciotomy)**			
Secondary direct wound closure	0	39 (39)	39 (34)
Skin grafting	0	22 (22)	22 (19)
Debridement	1	21 (21)	22 (19)
Amputation	0	2 (2)	2 (2)
Other ^3^	0	14 (14)	14 (12)
**Complications**			
Acute adverse reaction to antivenom	3 (21)	8 (8)	11 (10)
Postoperative haemorrhage	--	4 (4)	4 (3)
Wound infection	0	5 (5)	5 (4)
**Clinical outcome**			
Sequelae ^4^	0	26 (26)	26 (23)
Deceased	0	5 (5)	5 (4)
**Duration of hospitalization (days)**			
Median (IQR)	7 (5–7)	15 (10–28)	14 (7–24)
**Maximum follow-up time** ^1^			
Discharge and/or < 3 months	13	69	82 (71)
≥ 3 months	1	27	28 (24)

^1^Missing data for the following number of cases: Time bite-antivenom (n = 34), Maximum follow-up time (n=5 patients who died during hospitalization)

^2^Includes red blood cells, platelets, fresh frozen plasma and cryoprecipitate

^3^Includes the following procedures: Adhesiolysis, re-exploration of fasciotomy wound, bone-grafting, skin-flap/muscle flap placements, other (unspecified) plastic surgical procedures, wound irrigation,and arthrodesis

^4^Encompasses irreversible functional and tissue deficits such as abnormal or loss of sensation, muscular palsy, amputations/partial loss of digit, joint contractures and limited mobility of affected limbs. Two patients were reported to have sequelae of unspecified nature.

Of the 10 patients initially managed non-surgically with serial compartment pressure monitoring (Fig 2), nine received antivenom. In seven of these patients, pressure decreased in at least one of the measured compartments (Fig 2). In four patients, the pressure decreased to an absolute value of 30 mmHg or lower on the second reading. The maximum reported decrease in compartment pressure between the first two measurements, following the administration of antivenom, and in the absence of fasciotomy/dermotomy, was 31 mmHg [30]. Five patients were treated with fasciotomy following two or more serial compartment pressure measurements. In these patients, serial measurements indicated either further increased (n = 3) [43,91,124] or unimproved/elevated pressures (n = 1) [42]. In one patient [85], fasciotomies were performed due to clinical deterioration despite significant reductions in compartment pressure to almost normal levels.

Of all patients treated with fasciotomy and antivenom, 63 (84%) received antivenom before undergoing surgery. The median time between start of antivenom treatment and fasciotomy was 4h (IQR 0h–13h, missing data for n = 65). Fasciotomies were performed at a median time of 18h (IQR 8–48h, missing data for n = 28) after the bite. The minimum recorded time was 1h 20min and the maximum recorded time between bite and fasciotomy was seven days. Intraoperative findings were reported for 34 patients (34%), of whom 16 had intraoperative signs of tissue pressure elevation (tightness, bulging or oedematous muscle), while two did not. Necrotic, ischaemic or an otherwise abnormal appearance of muscle tissue was described in 19 patients during fasciotomy. Seven of the patients with abnormal intraoperative muscle appearance had maximum compartment pressures greater than 40 mmHg. None of the patients in whom compartment pressure was measured and with necrotic or ischaemic muscle appearance intraoperatively had pressures less than 30 mmHg. Absence of intraoperative muscle ischaemia or necrosis was noted in eight patients, of whom four had maximum compartment pressures of 30 mmHg or greater.

Twenty-five patients (22%) were given blood products, such as frozen plasma (n = 21; 19%), red blood cells (n = 12; 10%) and platelets (n = 9; 8%). Less frequently administered blood products included cryoprecipitate, fibrinogen and whole blood. Twenty-four patients (20%) required intubation/intensive care treatment, 18 of whom received intensive care prior to any surgical procedures being performed. Excessive intra-or post-operative haemorrhage was reported in four patients [46,89,93,101], three of whom [46,89,101] were diagnosed with a coagulopathy preoperatively (Table 3). Sequelae were reported more frequently for patients treated with fasciotomy, including the only recorded amputations and deaths. A single patient in the group treated without fasciotomy underwent tissue debridement of the bite site following a puff adder bite [64] (Table 3). Causes of death included generalized haemorrhage and multiple organ failure (n = 1) [45], uncontrolled postoperative haemorrhage (n = 1) [65], acute kidney injury (n = 1) [77], multiple organ failure, disseminated intravascular coagulation and bowel necrosis/peritonitis (n = 1) [125], and sepsis (n = 1) [61].

### General literature

Twenty-eight records from the general literature, including eight animal studies [25,26,133–138] (S4 File), 17 human observational studies [10,14,17,20,27,139–150] (S5 File) and three guidelines/expert recommendations [12,13,31], were deemed eligible for inclusion (Fig 1). All animal studies were conducted in the USA between 1979 and 2004 using animal models involving dogs (n = 3), rabbits (n = 2), and pigs (n = 3) injected with venom of North American rattlesnakes (*Crotalus atrox* and *Crotalus viridis helleri*). Human observational studies were mostly reported from the Americas (n = 6), followed by the Western Pacific Region (n = 5) and the Eastern Mediterranean (n = 2).

### Compartment pressures in intramuscular and subcutaneous envenoming and diagnosis of VICS

Four animal studies [25,133,135,137] showed that intramuscular injection of cytotoxic snake venom results in increased compartment pressures which exceeded the commonly accepted critical thresholds of 30–45 mmHg within the first two hours of venom administration [25]. A single animal study [138] reported no notable increases in compartment pressures following intramuscular venom injection. In two animal studies involving the subcutaneous injection of cytotoxic snake venom (*Crotalus viridis helleri*), neither elevated compartment pressures nor muscle necrosis ensued [133,137]. One human study [142] reported higher subcutaneous than intramuscular tissue pressures for two snakebite patients, with subcutaneous pressures ranging from 5–40 mmHg and intramuscular pressures ranging from 10–20 mmHg for the entire group of patients.

Cases with VICS were reported in 15/17 of the human observational studies included in this review. Two studies [20,142] were included in the analysis because they explored diagnostic approaches to VICS in snakebite patients, even though in both studies no patients were diagnosed with VICS. The percentage of hospitalized patients with snakebite envenoming diagnosed with VICS ranged from 0–69% [20,146]. The included clinical studies utilized the following diagnostic methods: compartment pressure measurement (n = 9) [10,14,139,140,142,145,146,148,150], ultrasound (n = 2) [20,141], limb circumference measurements (n = 2) [140,145], and venous oxygen saturation (SpO_2_) measurement (n = 1) [150].

Clinical diagnostic features of VICS were compared with compartment pressure measurements in four studies [139,140,142,148], all of which suggested that reliance on clinical symptoms alone could lead to an overdiagnosis of VICS. One human study [142] reported that in six patients with snakebite-induced limb swelling, hallmark signs of compartment syndrome, such as sensorimotor deficits, were present despite all patients having normal compartment pressures (less than 20 mmHg). In the other three studies, a clinical suspicion of VICS was confirmed with compartment pressure measurement in only 52% [148], 11% [139] and 8% [140] of patients respectively. Importantly, diagnostic criteria between the studies varied, and two studies [140,148] defined VICS based on elevated compartment pressures during serial measurements, meaning that initially elevated pressures that reduced over time were not counted toward a diagnosis of VICS.

The role of ultrasound in the diagnosis of VICS has been explored by two studies and has focused on two parameters: measurement of tissue swelling and arterial blood flow. In 42 patients with snakebite-induced limb swelling in South Africa, the sonographically determined thickness of intramuscular tissue was increased by an average of 6% in bitten limbs, compared to an increase of 100% in subcutaneous tissue [141]. The only patient in the study diagnosed with VICS also had the highest recorded increase of intramuscular tissue thickness (40%). The predilection of snakebite-induced swelling for the subcutaneous tissue plane was likewise demonstrated in 27 patients in Taiwan [20], none of whom showed sonographic signs of arterial compromise or were diagnosed with VICS.

The measurement of limb circumferences was reported in two studies [140,145], one of which specifically evaluated the diagnostic accuracy of circumference measurements for identifying patients with VICS [145]. Using a compartment pressure of at least 40 mmHg for confirming the presence of VICS, the authors reported a sensitivity and specificity of 76.9% and 66.7% linked to a circumference difference above the knee between bitten and non-bitten limbs of at least 2.8 cm [145]. One study including four snakebite patients concluded that SpO_2_ is not a reliable indicator for elevated compartment pressures [150].

Two WHO snakebite management guidelines for Africa and Asia [13,31] and one evidence-informed consensus recommendation on the management of VICS in North American *Crotalinae* envenoming were included in this review [12]. All three highlighted that clinical signs of compartment syndrome are unreliable in envenomed patients and may lead to overdiagnosis of VICS, recommending (serial) measurement of compartment pressures instead. In a word of caution, the evidence-informed consensus recommendation highlights that suspected VICS in digits, hands and feet cannot be reliably assessed using standard tools for compartment pressure measurement.

### Treatment of VICS and clinical outcomes

Treatments explored in animal studies included fasciotomy/fasciectomy, antivenom, glucocorticoids and pressure immobilization. Three animal studies [26,133,137] showed that fasciectomy/fasciotomy reduced compartment pressures, although the level of muscular necrosis in animals that underwent fasciotomy/fasciectomy appeared unaltered by the procedure. One additional animal study [135] likewise found that fasciotomy/fasciectomy did not reduce the level of muscle necrosis and one study reported an increased extent of myonecrosis following fasciotomy/fasciectomy [26].

The effect of antivenom on compartment pressure and/or muscular necrosis was investigated in five animal studies [25,26,135,136,138]. In the study by Garfin et al. [25], increasing doses of antivenom were associated with incremental reductions in pressure post-envenoming. Similarly, in a porcine model [136], treatment with antivenom was associated with increased perfusion of envenomed limbs. With respect to muscular necrosis and preservation of limb function, rabbits treated with antivenom following intramuscular venom injection had preserved muscle mass, greater measured muscle tension and resistance to muscle fatigue compared to surgically treated or untreated animals [135]. Two studies [26,138] found that muscular necrosis was unaltered by antivenom treatment.

The largest proportion (n = 17, 89%) of human patients with confirmed VICS (compartment pressures exceeding 40 mmHg) treated non-surgically were reported in a study from China [145]. Non-surgical treatment methods were evaluated using serial pressure measurements in two studies (seven patients total), all of whom had a reduction in compartment pressure from greater than 30 mmHg on the first measurement to less than 30 mmHg on the second measurement [10,145]. One study showed in a multivariate analysis that antivenom was associated with a lower risk of developing VICS [17]. In a Moroccan hospital, the percentage of patients treated with fasciotomy reduced after antivenom and a new snakebite treatment protocol were made available (47% vs 20%) [143].

Fasciotomies were performed on all patients diagnosed with VICS in 7/15 studies. Muscular necrosis determined intraoperatively was reported in three studies in 2/25 [146], 6/9 [147] and 1/1 patients who underwent fasciotomy [150]. The following complications were reported from nine studies (total n = 56 patients) for patients treated with fasciotomy: Requirement for blood products (16–86%) [146,149], wound infections (8–100%) [144,146,149], soft tissue complications, including need for debridement, skin grafting, or tissue flap (67%) [147], amputation (11–60%) [139,147], and two deaths [139,149]. A single patient (out of a total of n = 56) with VICS treated non-surgically was reported to have developed Volkmann’s ischaemic contracture, resulting in limb amputation [10].

The WHO guidelines and the North American expert-based consensus statement for the management of VICS all recommend initial non-surgical treatment using antivenom, guided by serial compartment pressure monitoring. In addition, the expert-based consensus recommendation from North America advises against prophylactic fasciotomies, recommending fasciotomies only if initial treatment with antivenom fails.

## Discussion

### Epidemiology of VICS

To our knowledge, this is the first literature review to compile clinical data from snakebite patients diagnosed with VICS worldwide. Despite the mortality and morbidity burden of snakebite envenoming being concentrated predominantly in tropical and subtropical low- and middle-income countries (LMICs) [18,151], half of all globally published case reports on VICS are from high-and middle-income countries in Europe or the USA. Only a small proportion (19%) of case reports originated from Africa and South-East Asia, two regions that rank highest in snakebite envenoming incidence and snakebite-associated mortality [18,152,153]. The rarity [14] and the special clinicopathologic entity of VICS [7,12,23], especially if caused by exotic captive snakes, combined with readily available diagnostic confirmation using compartment pressure measurements, are likely drivers of case reporting from Europe and the USA. The lower reporting from snakebite-endemic lower-middle-income (LMIC) settings may be related to the perception of snakebite envenoming and VICS in particular not being an unusual disease entity [154–156], lack of awareness and recognition of VICS [157], absence of tools for diagnostic confirmation, lack of surgical expertise and treatment capacity as well as insufficient access to resources and training for authoring publications [158].

Viperid bites were by far the most frequent cause of reported VICS; only 3% of bites could be attributed to *Elapidae*. Snakes of both the *Viperidae* and *Elapidae* families produce venoms with potently tissue destructive effects [159] and *Elapidae*, most notably spitting cobras, are a major cause of snakebite-related soft tissue injury in Sub-Saharan Africa [160–162] and South-East Asia [147,163]. The low number of elapid bites reported in this sample could be partly related to the underreporting of cases from these two regions. Nonetheless, the longer fangs [164], higher strike velocities [165] and the venom proteomes of viperids may all lead to a higher risk of subfascial venom deposition and VICS [163]. Data from animal experiments, in which elevated compartment pressures could only be induced by intramuscular, but not subcutaneous venom injections, highlight venom injection depth as an important risk factor for VICS [133,137]. Finally, from a pathological viewpoint, viperid envenoming is more likely to cause swelling and elevated tissue pressures [166]. Some viperid venoms are rich in snake venom metalloproteinases, which degrade extracellular matrix and basement membranes of blood vessels, leading to massive and diffuse fluid extravasation, haemorrhage and secondary ischaemic changes, ultimately setting the stage for a vicious cycle of uncontained swelling, inflammation and pressure increase [6,138,166]. Haemorrhage and fluid extravasation are exacerbated by venom-induced coagulopathy–a frequent occurrence in this sample of VICS patients. Cytotoxic elapid venoms, in contrast, induce patterns of tissue damage that are more focal and superficial and are seldomly a cause of coagulopathy [162,163,166].

### Diagnosis of VICS

Establishing a reliable diagnosis of compartment syndrome in the envenomed patient remains a major challenge. Reliance on clinical symptoms alone is associated with a high false positive rate [140,142,146,148] and the risk of unnecessary fasciotomies [167]. Our search retrieved only four diagnostic studies which investigated objective measurement methods for diagnosing VICS [20,141,145,148]. Only two of these studies [145,148] reported diagnostic reference criteria for VICS. The non-reporting of diagnostic criteria greatly limits the interpretability and comparability of the limited data that is available on this subject. Currently, measurement of compartment pressure provides the only objective means of establishing a diagnosis of compartment syndrome, with an estimated sensitivity and specificity in acute traumatic compartment syndrome of 0.94 and 0.98, respectively [168]. A variety of methods, some of which can be improvised [169–171], exist to measure compartment pressure, but simpler point-of-care tools are unavailable or prohibitively expensive in the context of LMICs [66,78,123,172]. This is reflected in the fact that most compartment pressure measurements in this study were performed in well-resourced healthcare settings in Europe or the Americas. Our data shows that compartment pressure measurements, particularly serial pressure measurements, were more frequently performed in patients managed without fasciotomy. Diagnostic tools for continuous monitoring of compartment pressures are a prerequisite for responsibly and safely using antivenom as a stand-alone treatment for suspected VICS and importantly, to prompt an emergency fasciotomy if non-surgical measures fail. Continuous pressure monitoring could generate observational data in patients with VICS and serve as a diagnostic reference standard in studies seeking to explore alternative diagnostic modalities [173].

However, certain challenges with compartment pressure measurement will remain. It requires training to safely target individual muscle compartments and to avoid iatrogenic injury, it is invasive and it carries the risk of inducing infection and haemorrhage in the coagulopathic patient [39]. Bites to hands, fingers and feet accounted for 51% of all patients with VICS and nearly one third of all anatomic locations in which pressure measurements were performed. In acute traumatic compartment syndrome, pressure monitoring in hands and feet has been suggested to improve diagnostic accuracy compared with clinical examination alone [22,174,175]. However, expert consensus on the surgical management of VICS states that compartment pressure measurements cannot be reliably obtained in hands, fingers and feet [12,167]. The challenge of obtaining pressure readings in hands and feet pertains to their many muscle compartments–ten in the hand [174], and nine in the foot [22]–and their relatively small sizes, which complicate accurate needle placement and reliable pressure readings. The large proportion of VICS cases affecting distal extremities combined with the limitations of invasive pressure monitoring in this subset of patients underlines the need to explore alternative diagnostic tools for VICS. Point-of care ultrasound holds promise as a non-invasive diagnostic modality [20,141,176,177] for differentiating subcutaneous from intramuscular envenoming, but additional research is required to establish optimal measurement parameters and importantly, their association with compartment pressure and perfusion.

### Treatment of VICS

In contradistinction to traumatic compartment syndrome, where fasciotomy is the only definitive treatment to restore limb perfusion, antivenom is a potential alternative treatment in VICS. Animal experiments have demonstrated a decrease in compartment pressure [25], increases in perfusion pressure [136] and when compared to fasciotomy alone, improved preservation of muscle mass and limb function [135] following antivenom administration. While experimental animal data is based on (North American) viperid envenoming–the most commonly studied snake family in VICS–significant interspecies variation in venom composition [178] limits the extrapolation of these findings outside of North America. We could not find cohort-level data on compartment pressure changes following antivenom treatment, although in some studies this data appeared to have been collected but, unfortunately, not fully reported [145,148]. Although causation cannot be inferred, considerable decreases in compartment pressure following antivenom administration were recorded in five published patient reports. None of these patients required an amputation or developed ischaemic muscle contracture.

Initial management of VICS with antivenom is widely recommended, especially when signs of systemic envenoming, in particular coagulopathy, are present [13,31,167]. Definitive treatment with fasciotomy cannot be safely performed until clotting ability has been restored [13,31], a time window during which compartment pressures can be serially re-assessed [12] without the ethical dilemma of deferring a potentially beneficial treatment (fasciotomy) in favor of an alternative for which high-quality clinical evidence is still lacking. Serial pressure measurement may avert fasciotomies in some cases and generate important observational data, which may strengthen the evidence for antivenom being an effective stand-alone treatment for VICS. New modalities, such as small-molecule inhibitors [179], could also be effective. Given the rarity of VICS, a future clinical trial is unlikely. However, collecting high-quality clinical observational data in patients with suspected VICS during trials investigating treatments for snakebite envenoming could be very beneficial. Furthermore, registries such as the North American Snake Bite Registry and poison center reports can be an invaluable source of epidemiological and clinical data on VICS; for example showing important trends in diagnostic and treatment approaches over time, while tracking clinical outcomes [14,27,180,181].

Several factors associated with the non-surgical management of VICS in this sample require highlighting. Firstly, most in this group were treated in high-income settings in Europe and the USA. Rapid presentation of patients to hospitals allows clinicians to reliably estimate limb ischaemia time, which helps estimate the time at which irreversible tissue damage may occur if compartment pressure is not reduced. This information is crucial when deferring a fasciotomy, which provides rapid relief of elevated compartment pressure, in favor of antivenom as a stand-alone treatment, which may take longer to reduce pressure. Access to antivenom, improved diagnostic modalities and the availability of clinical snakebite expertise would support decision-making during the non-invasive management of VICS [27]. Intervening on these factors in LMIC settings has the potential to reduce the overdiagnosis of VICS, to lessen the number of unnecessary fasciotomies being performed and to make the fasciotomies that are ultimately required safer [31,167].

Our data shows that clinicians must maintain a high vigilance for coagulopathies, present in 40% of patients in this study, when treating VICS [31,167]. Despite this, intra- and postoperative haemorrhage were reported only in four patients, one of whom suffered fatal exsanguination following fasciotomy. Abnormal clotting was probably restored in many patients with more severe coagulopathy, given the frequent use of antivenom and blood products and the considerable time intervals between antivenom infusion and fasciotomies in many cases.

Altogether, this scoping review underscores that decisions in the clinical management of VICS are highly context-dependent and must factor in the local and systemic toxicities associated with different snake species as well as the available diagnostic equipment, antivenom stocks, surgical capacity and resources for adjunctive and post-operative care.

### Strengths and limitations

Our review is the first to globally synthesize evidence from case reports on VICS and our literature search covered three large databases without language restrictions. We had to rely on author-reported diagnoses of VICS instead of a priori defined diagnostic criteria, for which no universal consensus definition exists. This invariably resulted in a clinically heterogeneous patient population and some of the included patients may have been falsely diagnosed with VICS. Conversely, it is likely that patients with VICS who had an uneventful recovery following antivenom treatment were never described in the scientific literature. Our analysis of cases was limited by incomplete reporting of clinical data, and unclarity about the chronology of events. Adherence to international reporting standards, as formulated in the CARE checklist [182], could improve the quality of data from future cases of VICS. It is important to acknowledge that our sample is most likely subject to publication bias, which likely favored the reporting of surgically treated patients, and that it does not constitute a representative sample of VICS patients. The heterogeneity of involved snake species, differing management settings and antivenom products in addition to the likely presence of indication bias precluded us from performing in-depth statistical analyses, in particular comparisons between treatment groups. Lastly, we did not perform a formal appraisal of the scientific quality of selected literature, which would extend beyond the objectives of a scoping review.

## Conclusion

VICS is a rare but potentially debilitating complication of snakebite, and its clinical management is associated with multiple challenges. Uniform diagnostic criteria and widely available point-of-care diagnostic tools for objectively diagnosing VICS remain elusive. Compartment pressure measurement is the currently established standard for diagnosing VICS, but it is invasive and largely unavailable in resource-constrained settings. Ultrasound holds promise to distinguish intramuscular from subcutaneous swelling, but further studies are necessary to establish its diagnostic value in VICS. Developing and making available tools for reliable and continuous monitoring of compartment pressure has the potential to support decision-making and to avert fasciotomies when antivenom as a first-line treatment is effective in VICS. The common co-occurrence of VICS and coagulopathy underscores the importance of initial treatment with antivenom, providing a critical time window during which compartment status can be reassessed for potential reductions in pressure. Finally, observational data will remain a cornerstone of evidence synthesis for this rare but potentially debilitating condition, emphasizing the need to expand and improve upon the reporting of cases in the scientific literature.

## Supporting information

S1 File PRISMA-ScR checklist - From: Tricco AC, Lillie E, Zarin W, O’Brien KK, Colquhoun H, Levac D, et al. PRISMA Extension for Scoping Reviews(PRISMAScR): Checklist and Explanation. Ann Intern Med. 2018;169:467–473. https://doi.org/10.7326/M18-0850
(PDF)

S2 File Search strategy. (PDF)

S3 File Case reports. (DOCX)

S4 File Animal studies. (DOCX)

S5 File General literature. (DOCX)

## References

[1] World Health Organization. Snakebite envenoming- A strategy for prevention and control. Geneva; 2019 https://www.who.int/publications/i/item/9789241515641 10.1016/S2214-109X(19)30225-6 31129124

[2] Fry BG. Snakebite: When the Human Touch Becomes a Bad Touch. Toxins (Basel). 2018;10(4):170. doi: 10.3390/toxins10040170 29690533 PMC5923336

[3] Brenes-Chacón H, Gutiérrez JM, Camacho-Badilla K, Soriano-Fallas A, Ulloa-Gutierrez R, Valverde-Muñoz K, et al. Snakebite envenoming in children: A neglected tropical disease in a Costa Rican pediatric tertiary care center. Acta Trop. 2019;200:105176. doi: 10.1016/j.actatropica.2019.105176 31526777

[4] Naik BS. “Dry bite” in venomous snakes: A review. Toxicon. 2017;133:63–7. doi: 10.1016/j.toxicon.2017.04.015 28456535

[5] Nascimento dCT, Mota-da-Silva A, Colombini M, Moura-da-Silva AM, Medeiros dSR, Monteiro WM, et al. Relationship between snake size and clinical, epidemiological and laboratory aspects of Bothrops atrox snakebites in the Western Brazilian Amazon. Toxicon. 2020;186:160–7. 32822734 10.1016/j.toxicon.2020.08.010

[6] Gutiérrez JM, Rucavado A, Escalante T, Herrera C, Fernández J, Lomonte B, et al. Unresolved issues in the understanding of the pathogenesis of local tissue damage induced by snake venoms. Toxicon. 2018;148:123–31. doi: 10.1016/j.toxicon.2018.04.016 29698755

[7] Newman J, Therriault C, White MS, Nogee D, Carpenter JE. Compartment Syndrome Following Snake Envenomation in the United States: A Scoping Review of the Clinical Literature. West J Emerg Med. 2024;25(4):651–60. doi: 10.5811/westjem.18401 39028252 PMC11254155

[8] Volkmann R. Ischaemic muscle paralyses and contractures. J Hand Surg Br. 2005;30(2):233–4. doi: 10.1016/j.jhsb.2004.10.008 15757782

[9] Stracciolini A, Hammerberg E. UpToDate. Acute compartment syndrome of the extremities. 2019. https://www.uptodate.com/contents/acute-compartment-syndrome-of-the-extremities

[10] Otero R, Gutiérrez J, Beatriz MM, Duque E, Rodríguez O, Luis AJ, et al. Complications of Bothrops, Porthidium, and Bothriechis snakebites in Colombia. A clinical and epidemiological study of 39 cases attended in a university hospital. Toxicon. 2002;40(8):1107–14. 12165312 10.1016/s0041-0101(02)00104-6

[11] Elbey B, Baykal B, Yazgan ÜC, Zengin Y. The prognostic value of the neutrophil/lymphocyte ratio in patients with snake bites for clinical outcomes and complications. Saudi J Biol Sci. 2017;24(2):362–6. doi: 10.1016/j.sjbs.2015.10.002 28149174 PMC5272947

[12] Toschlog EA, Bauer CR, Hall EL, Dart RC, Khatri V, Lavonas EJ. Surgical considerations in the management of pit viper snake envenomation. J Am Coll Surg. 2013;217(4):726–35. doi: 10.1016/j.jamcollsurg.2013.05.004 23891068

[13] World Health Organization. Guidelines for the Prevention and Clinical Management of Snakebite in Africa. Brazzaville; 2010. https://www.who.int/publications-detail-redirect/9789290231684

[14] Spyres MB, Maker G, Aldy K, Wolk BJ, Meadors KE, Christian M, et al. Compartment Syndrome after Crotalid Envenomation in the United States: A Review of the North American Snakebite Registry from 2013 to 2021 on Behalf of the ToxIC Snakebite Study Group. Wilderness Environ Med. 2023;34(3):322–7. doi: 10.1016/j.wem.2023.05.007 37474357

[15] Hernandez MC, Traynor M, Bruce JL, Bekker W, Laing GL, Aho JM, et al. Surgical Considerations for Pediatric Snake Bites in Low- and Middle-Income Countries. World J Surg. 2019;43(7):1636–43. 30783764 10.1007/s00268-019-04953-9

[16] Aktar F, Aktar S, Yolbas I, Tekin R. Evaluation of Risk Factors and Follow-Up Criteria for Severity of Snakebite in Children. Iran J Pediatr. 2016;26(4):e5212. doi: 10.5812/ijp.5212 27729959 PMC5047028

[17] Hsieh Y-H, Hsueh J-H, Liu W-C, Yang K-C, Hsu K-C, Lin C-T, et al. Contributing Factors for Complications and Outcomes in Patients With Snakebite: Experience in a Medical Center in Southern Taiwan. Ann Plast Surg. 2017;78(3 Suppl 2):S32–6. doi: 10.1097/SAP.0000000000001002 28195896

[18] Kasturiratne A, Wickremasinghe AR, de Silva N, Gunawardena NK, Pathmeswaran A, Premaratna R, et al. The global burden of snakebite: a literature analysis and modelling based on regional estimates of envenoming and deaths. PLoS Med. 2008;5(11):e218. doi: 10.1371/journal.pmed.0050218 18986210 PMC2577696

[19] Sachett J de AG, Val FF, Alcântara JA, Cubas-Vega N, Montenegro CS, da Silva IM, et al. Bothrops atrox Snakebite: How a Bad Decision May Lead to a Chronic Disability: A Case Report. Wilderness Environ Med. 2020;31(3):317–23. doi: 10.1016/j.wem.2020.03.001 32456876

[20] Ho C-H, Ismail AK, Liu S-H, Tzeng Y-S, Li L-Y, Pai F-C, et al. The role of a point-of-care ultrasound protocol in facilitating clinical decisions for snakebite envenomation in Taiwan: a pilot study. Clin Toxicol (Phila). 2021;59(9):794–800. doi: 10.1080/15563650.2021.1881535 33605805

[21] Chang J. Global reconstructive surgery. Elsevier Inc.; 2018. 403 p.

[22] von Keudell AG, Weaver MJ, Appleton PT, Bae DS, Dyer GSM, Heng M, et al. Diagnosis and treatment of acute extremity compartment syndrome. Lancet. 2015;386(10000):1299–310. doi: 10.1016/S0140-6736(15)00277-9 26460664

[23] Cumpston KL. Is there a role for fasciotomy in Crotalinae envenomations in North America?. Clin Toxicol (Phila). 2011;49(5):351–65. doi: 10.3109/15563650.2011.597032 21740134

[24] Corneille MG, Larson S, Stewart RM, Dent D, Myers JG, Lopez PP, et al. A large single-center experience with treatment of patients with crotalid envenomations: outcomes with and evolution of antivenin therapy. Am J Surg. 2006;192(6):848–52. doi: 10.1016/j.amjsurg.2006.08.056 17161106

[25] Garfin SR, Castilonia RR, Mubarak SJ, Hargens AR, Akeson WH, Russell FE. The effect of antivenin on intramuscular pressure elevations induced by rattlesnake venom. Toxicon. 1985;23(4):677–80. doi: 10.1016/0041-0101(85)90372-1 4060178

[26] Tanen DA, Danish DC, Grice GA, Riffenburgh RH, Clark RF. Fasciotomy worsens the amount of myonecrosis in a porcine model of crotaline envenomation. Ann Emerg Med. 2004;44(2):99–104. doi: 10.1016/j.annemergmed.2004.01.009 15278079

[27] Darracq MA, Cantrell FL, Klauk B, Thornton SL. A chance to cut is not always a chance to cure- fasciotomy in the treatment of rattlesnake envenomation: A retrospective poison center study. Toxicon. 2015;101:23–6. doi: 10.1016/j.toxicon.2015.04.014 25935457

[28] Gold BS, Barish RA, Dart RC, Silverman RP, Bochicchio GV. Resolution of compartment syndrome after rattlesnake envenomation utilizing non-invasive measures. J Emerg Med. 2003;24(3):285–8. doi: 10.1016/s0736-4679(02)00762-x 12676299

[29] Shaw BA, Hosalkar HS. Rattlesnake bites in children: antivenin treatment and surgical indications. J Bone Joint Surg Am. 2002;84(9):1624–9. 12208920

[30] Mazer-Amirshahi M, Boutsikaris A, Clancy C. Elevated compartment pressures from copperhead envenomation successfully treated with antivenin. J Emerg Med. 2014;46(1):34–7. doi: 10.1016/j.jemermed.2013.05.025 23871482

[31] World Health Organization. World Health Organization. 2016. p. 201 Guidelines for The Management of Snakebites, 2nd edition.

[32] Cañas CA. Is the acute compartment syndrome diagnosed in snake bites true?: A review. Medicine (Baltimore). 2024;103(40):e40008. doi: 10.1097/MD.0000000000040008 39465701 PMC11460897

[33] JBI Manual for Evidence Synthesis. JBI Manual for Evidence Synthesis. 2020.

[34] Tricco AC, Lillie E, Zarin W, O’Brien KK, Colquhoun H, Levac D, et al. PRISMA Extension for Scoping Reviews (PRISMA-ScR): Checklist and Explanation. Ann Intern Med. 2018;169(7):467–73. doi: 10.7326/M18-0850 30178033

[35] Center for Open Science (OSF). Protocol: Clinical Management Of Compartment Syndrome Secondary To Snakebite Envenoming: A Global Scoping Review. 2022 https://osf.io/w4pe2/overview

[36] The EndNote Team. EndNote. Philadelphia, PA: Clarivate; 2013.

[37] Ouzzani M, Hammady H, Fedorowicz Z, Elmagarmid A. Rayyan-a web and mobile app for systematic reviews. Syst Rev. 2016;5(1):210. doi: 10.1186/s13643-016-0384-4 27919275 PMC5139140

[38] Our World in Data. World regions according to the World Health Organization. 2023 https://ourworldindata.org/grapher/who-regions

[39] Bucaretchi F, De Capitani EM, Hyslop S, Mello SM, Fernandes CB, Bergo F, et al. Compartment syndrome after South American rattlesnake (Crotalus durissus terrificus) envenomation. Clin Toxicol (Phila). 2014;52(6):639–41. doi: 10.3109/15563650.2014.913177 24940645

[40] R Core Team (R Foundation for Statistical Computing). R: A Language and Environment for Statistical Computing. Vienna, Austria; 2025.

[41] Thomas DK, Budhoo EJ, Mencia MM, Ali TF, Santana D. A Case of Upper Limb Compartment Syndrome following Snake Envenomation: Measure Twice, Cut Once. West Indian Med J. 2014;63(4):384–7. doi: 10.7727/wimj.2013.261 25429488 PMC4663926

[42] Brys AK, Gandolfi BM, Levinson H, Gerardo CJ. Copperhead Envenomation Resulting in a Rare Case of Hand Compartment Syndrome and Subsequent Fasciotomy. Plast Reconstr Surg Glob Open. 2015;3(5):e396. doi: 10.1097/GOX.0000000000000367 26090286 PMC4457259

[43] Bucaretchi F, de Capitani EM, Hyslop S, Mello SM, Madureira PR, Zanardi V, et al. Compartment syndrome after Bothrops jararaca snakebite: monitoring, treatment, and outcome. Clin Toxicol (Phila). 2010;48(1):57–60. doi: 10.3109/15563650903356201 20095815

[44] Hardy DL Sr, Zamudio KR. Compartment syndrome, fasciotomy, and neuropathy after a rattlesnake envenomation: aspects of monitoring and diagnosis. Wilderness Environ Med. 2006;17(1):36–40. doi: 10.1580/1080-6032(2006)17[36:csfana]2.0.co;2 16538944

[45] Mendez-Dominguez N, Gomez-Carro S, Diaz-Novelo R, Bobadilla-Rosado LO, Chi-Mendez C. Emergency treatment for a venomous snakebite accident in rural southern Mexico. Rural Remote Health. 2019;19(2):4701. doi: 10.22605/RRH4701 30966755

[46] McBride KM, Bromberg W, Dunne J. Thromboelastography Utilization in Delayed Recurrent Coagulopathy after Severe Eastern Diamondback Rattlesnake Envenomation. Am Surg. 2017;83(4):332–6. doi: 10.1177/000313481708300417 28424125

[47] Valente-Aguiar MS, Gonçalves dCESB, Magalhães T, Dinis-Oliveira RJ. Compartment Syndrome following Bothrops Snakebite Leads to Decompressive Fasciotomies. Vol. 2019, Case reports in medicine. 2019:6324569. 10.1155/2019/6324569 PMC6425384 30949208

[48] Resiere D, Gautier M, Valentino R, Mehdaoui H, Mégarbane B. Bothrops lanceolatus envenomation in a patient with arteriovenous fistula for hemodialysis access. Clin Toxicol (Phila). 2016;54(5):460–1. doi: 10.3109/15563650.2016.1144888 26919642

[49] Rosen PB, Leiva JI, Ross CP. Delayed antivenom treatment for a patient after envenomation by Crotalus atrox. Ann Emerg Med. 2000;35(1):86–8. doi: 10.1016/s0196-0644(00)70111-9 10613947

[50] Thomas KC, Crouch BI, Caravati EM. Elevated compartment pressures in two pediatric rattlesnake envenomations. In: Clinical Toxicology. 2011. p. 541.

[51] Ruha AM. Finger Debridement And Amputation After Rattlesnake Envenomation: A Case Series. Abstr Clin Toxicol. 2011;49(6):515–627.

[52] Roberts RS, Csencsitz TA, Heard CW Jr. Upper extremity compartment syndromes following pit viper envenomation. Clin Orthop Relat Res. 1985;(193):184–8. doi: 10.1097/00003086-198503000-00026 3971621

[53] Oporta EA. Síndrome compartimental por envenenamento ofídico. (Manejo de fasciotomía con terapia VAC). Rev Médica Costa Rica y Centroamérica. 2010;67(594):405–15.

[54] Campbell BT, Corsi JM, Boneti C, Jackson RJ, Smith SD, Kokoska ER. Pediatric snakebites: lessons learned from 114 cases. J Pediatr Surg. 2008;43(7):1338–41. doi: 10.1016/j.jpedsurg.2007.11.011 18639692

[55] Cavazos DR, Schultz R, Higginbotham DO, Goethals J, Vaidya R. Refractory compartment syndrome after antivenom administration for an Eastern Diamondback Rattlesnake bite requiring fasciotomy for limb salvage: A case report. Trauma Case Rep. 2023;46:100852. doi: 10.1016/j.tcr.2023.100852 37274542 PMC10238865

[56] Lazar A, Dicker F, Semple M, Baumgartner K, Liss D. Hand compartment pressures decrease after Crotalidae polyvalent immune fab in suspected timber rattlesnake (Crotalus horridus) envenomation. Clin Toxicol. 2023;61:117–8.

[57] Lizarzaburu-Ortiz C, Yumi G, Carvajal A, Pachacama AB, Berrazueta A, Rojas E. A Rare and Urgent Consequence After a Snake Bite. Cureus. 2022;14(2):e21910. doi: 10.7759/cureus.21910 35265431 PMC8898570

[58] Yakey B, Dimovska M, Isaacson A, Vaidya R, Dolcourt B. Eastern diamondback rattlesnake envenomation with severely elevated compartment pressures despite antivenin administration. Clin Toxicol. 2022;60:33–4.

[59] Lund J, Peredy T, Aleguas A. Compartment pressures requiring fasciotomy. Toxicon. 2020;182:S18. doi: 10.1016/j.toxicon.2020.04.046

[60] Navarro-Vergara A, Navarro-Fretes A. Síndrome compartimental agudo no traumático en pediatría. Serie de casos y revisión de tema. Acta Ortopédica Mex. 2025;39(3):173–9. 40645789

[61] Firth GB, Street M, Ramguthy Y, Doedens L. Mortality following snake bite envenomation by Bitis arietans in an HIV positive child: A case report. Medicine (Baltimore). 2016;95(27):e4001. doi: 10.1097/MD.0000000000004001 27399076 PMC5058805

[62] Gras S, Plantefève G, Baud F, Chippaux J. Snakebite on the hand: lessons from two clinical cases illustrating difficulties of surgical indication. J Venom Anim Toxins incl Trop Dis. 2012;18(4):467–77. doi: 10.1590/s1678-91992012000400019

[63] Kouassi KJE, Sery BLNJ, Yao LB. Syndrome des loges de l’avant-bras secondaire à une morsure de vipéridé chez l’enfant. Ann Fr Med Urgence. 2016;7(1):45–7. doi: 10.1007/s13341-016-0696-y

[64] Le Dantec P, Hervé Y, Niang B, Chippaux JP, Bellefleur JP, Boulesteix BdG. Morsure Par Vipèr Bitis Arietans Au Sénégal, Intérêt De La Mesure De Pression Intracompartimentale. Médecine Trop. 2004;64(2):187–91. 15460152

[65] Blaylock R. Epidemiology of snakebite in Eshowe, KwaZulu-Natal, South Africa. Toxicon. 2004;43(2):159–66. doi: 10.1016/j.toxicon.2003.11.019 15019475

[66] Schweitzer G, Lewis JS. Puff adder bite--an unusual cause of bilateral carpal tunnel syndrome. A case report. S Afr Med J. 1981;60(18):714–5. 7302727

[67] Blaylock RS. Femoral vessel entrapment and compartment syndromes following snakebite. S Afr J Surg. 2003;41(3):72–3. 14626892

[68] Tangtermpong A, Pinyopornpanish K, Vasaruchapong T, Chenthanakij B, Pinyopornpanish K. The Treatment of Unidentified Hematotoxic Snake Envenomation and the Clinical Manifestations of a Protobothrops kelomohy Bite. Wilderness Environ Med. 2021;32(1):83–7. doi: 10.1016/j.wem.2020.11.001 33516621

[69] Hon KLE, Chow CM, Cheung KL, Leung TF. Snakebite in a child: could we avoid the anaphylaxis or the fasciotomies?. Ann Acad Med Singap. 2005;34(7):454–6. doi: 10.47102/annals-acadmedsg.v34n7p454 16123822

[70] Panda R, Singh S, Radhakrishnan RV, Mohanty CR, Shaji IM, Prusty AV, et al. A Case of Cobra Bite in a Term Pregnant Woman: The Obstetric and Wound Management Challenges. Wilderness Environ Med. 2023;34(4):571–5. doi: 10.1016/j.wem.2023.09.007 37923681

[71] Rathnayaka RMMKN, Ranathunga PEAN, Kularatne SAM. Epidemiological and clinical features of hump-nosed pit viper (Hypnale hypnale and Hypnale zara) envenoming in children. PLoS Negl Trop Dis. 2022;16(12):e0011013. doi: 10.1371/journal.pntd.0011013 36548435 PMC9822102

[72] Senthilkumaran S, Arathisenthil SV, Williams J, Almeida JR, Williams HF, Rajan E, et al. Neutrophil-mediated erythrophagocytosis following Russell’s viper (Daboia russelii) bite. Toxicon. 2023;228:107111. doi: 10.1016/j.toxicon.2023.107111 37060927

[73] Jatsho J. Compartment Syndrome Complicating Snakebite; a Clinician’ s Dilemma. Clin Case Reports Int. 2020;4:1179.

[74] Bhattarai N, Dhakal P, Bhandari D, Katwal S, Bhandari S, Shrestha S. Compartment syndrome of upper limb following snake bite, a case report. Ann Med Surg (Lond). 2025;87(9):6197–200. doi: 10.1097/MS9.0000000000003674 40901144 PMC12401192

[75] Dahal A, Basi A, Shrestha R, Khadka SK, Das A, Malla M, et al. Compartment syndrome of arm secondary to snake bite on hand: a case report. Ann Med Surg (Lond). 2024;86(8):4832–5. doi: 10.1097/MS9.0000000000002253 39118675 PMC11305786

[76] Mariappan M, C J, S R. Acute Transverse Myelitis following ASV in South Tamil Nadu. Abstr Ann Indian Acad Neurol. 2024;27(Suppl 1):S175.

[77] Mahadevaiah V, Ravikumar K, Manjunath H. “Fanged to Peril”: Interesting Snake Bite Case Series. Cureus. 2024;16(1):e53319. 10.7759/cureus.53319 PMC10906932 38435905

[78] Namal Rathnayaka RMMK, Ranathunga PEAN, Abeyrathne YNMP, Kularatne DA, Kularatne SAM. Acute compartment syndrome leading to fasciotomy, severe morbidity and long-term disabilities following Sri Lankan Green pit viper (Peltopelor trigonocephalus) envenomation. Toxicon. 2024;252:108179. doi: 10.1016/j.toxicon.2024.108179 39557218

[79] Srivastava UK, Verma CS, Jha S, Verma UK, Verma P, Tripathi SS, et al. Cases Series of Snake Bite Presented to the Emergency Medicine Department of Tertiary Care Centre. Int J Pharm Clin Res. 2024;16(9):1562–5.

[80] Vigasio A, Battiston B, De Filippo G, Brunelli G, Calabrese S. Compartmental syndrome due to viper bite. Arch Orthop Trauma Surg. 1991;110(3):175–7. doi: 10.1007/BF00395805 2059546

[81] Al-Azzawi M, Al-Azzawi AJK. Case report: Compartment syndrome secondary to a snakebite; To cut or not to cut?. Journal of Pediatric Surgery Case Reports. 2016;11:7–8. doi: 10.1016/j.epsc.2016.05.005

[82] Anil AB, anil m, kara OD, Bal A, Özhan B, Aksu N. Mannitol Therapy in Three Cases with Severe Edema Due to Snakebite: Case Report. Turkiye Klinikleri J Med Sci. 2011;31(3):720–3. doi: 10.5336/medsci.2009-13354

[83] Arif T, Bartecka-Mino K, Hruby K. Vipera berus bite causing compartment syndrome in a 14-year-old boy. Clin Toxicol. 2014;52(4):295–443.

[84] Barani C, Mortamet G, Forli A. Upper limb compartment syndrome after a viper bite in a child: A case report. Hand Surg Rehabil. 2021;40(1):97–100. doi: 10.1016/j.hansur.2020.07.003 32781253

[85] Cawrse NH, Inglefield CJ, Hayes C, Palmer JH. A snake in the clinical grass: late compartment syndrome in a child bitten by an adder. Br J Plast Surg. 2002;55(5):434–5. doi: 10.1054/bjps.2002.3888 12372375

[86] Carvalho J, Moinho R, Macao P, Oliveira G. When snakebites complicate: a paediatric case with shock and compartment syndrome. BMJ Case Rep. 2021;14(2):e240206. doi: 10.1136/bcr-2020-240206 33563677 PMC7875306

[87] Pietrangiolillo Z, Frassoldati R, Leonelli V, Freschi R, Russomando A, Lucaccioni L, et al. Compartment syndrome after viper-bite in toddler: case report and review of literature. Acta Biomed. 2012;83(1):44–50. 22978057

[88] Faber K, Ceschi A, Botti P, Peruzzi S, Rauber-Lüthy C, Giampreti A, et al. Human Envenomation by Bitis parviocula (Ehtiopian Mountain Adder). Abstr 2010 Int Congr Eur Assoc Poisons Centres Clin Toxicol May 2010, Bordeaux, Fr Clin Toxicol. 2010;48(3):246.

[89] Anil AB, Anil M, Karaarslan U, Bal A, Aksu N. Compartment syndrome in a child following snakebite. Nobel Med. 2010;6(6):108–11.

[90] Grenc D, Brvar M, Steblovnik K, Knafelj R, Gorjup V, Zivec K. A case of severe envenoming by eastern diamondback rattlesnake. In: 36th International Congress of the European Association of Poisons Centres and Clinical Toxicologists (EAPCCT),24-27 May, 2016, Madrid, Spain, Clinical Toxicology. 2016. p. 488.

[91] Fuchs J, Weiler S, Meier J. Envenomation by a western green mamba (Dendroaspis viridis) - A report of three episodes in Switzerland. Toxicon. 2019 Oct;168:76–82. 10.1016/j.toxicon.2019.06.223 31254601

[92] Evers LH, Bartscher T, Lange T, Mailänder P. Adder bite: an uncommon cause of compartment syndrome in northern hemisphere. Scand J Trauma Resusc Emerg Med. 2010;18:50. doi: 10.1186/1757-7241-18-50 20854675 PMC2949668

[93] Glatstein M, Lerman L, Friedman S, Carbell G, Munchak I, Valla U, et al. Severe disseminated intravascular coagulation in a child envenomated by Echis coloratus and successful treatment with monovalent equine immunoglobulin G antivenom. Toxicon. 2019;167:82–6. doi: 10.1016/j.toxicon.2019.05.012 31150660

[94] Meyer-Rath JC. Kompartmentsyndrom der oberen Extremitaät nach Klapperschlangenbiss (Crotalus adamanteus)- ein Fallbericht. Aktuelle Traumatol. 2003;33(3):138–41.

[95] Le Roux GL, Rochet S, Godde F, Larréche S, Descatha A. Far from home: compartmental syndrome after envenomation by Crotalus atrox in metropolitan France. 41st Int Congr Eur Assoc Poisons Centres Clin Toxicol 25-28 May 2021, Virtual Meet Clin Toxicol. 2021;59(6):580.

[96] Rainer PP, Kaufmann P, Smolle-Juettner FM, Krejs GJ. Case report: Hyperbaric oxygen in the treatment of puff adder (Bitis arietans) bite. Undersea Hyperb Med. 2010;37(6):395–8. 21226389

[97] Parrilla-Parrilla JS, Muñoz SM, Soult RJA, Cano FJ, López CJD. Síndrome compartimental por mordedura de víbora. An Esp Pediatr. 2002;56(5):477–8. 12042128

[98] Schnecker K. Seltene Ursache eines Kompartmentsyndroms an Unterarm und Hand nach einer Schlangenbißverletzung. Unfallchirurgie. 1990;16(3):158–9. doi: 10.1007/bf02588014 2382320

[99] Kuzbari R, Seidler D, Deutinger M. Lokale Komplikationen nach einem Giftschlangenbiß. Handchirurgie, Mikrochirurgie, Plast Chir. 2022;26(1):48–50. 8150389

[100] Tucker SC, Josty I. Compartment syndrome in the hand following an adder bite. J Hand Surg Br. 2005;30(4):434–5. doi: 10.1016/j.jhsb.2005.04.004 15935533

[101] Top LJ, Tulleken JE, Ligtenberg JJM, Meertens JHJM, van der Werf TS, Zijlstra JG. Serious envenomation after a snakebite by a Western bush viper (Atheris chlorechis) in the Netherlands: a case report. Neth J Med. 2006;64(5):153–6. 16702615

[102] Tincu RC, Ghiorghiu Z, Tomescu D, Macovei RA. The Compartment Syndrome Associated with Deep Vein Thrombosis due to Rattlesnake Bite: A Case Report. Balkan Med J. 2017;34(4):367–70. doi: 10.4274/balkanmedj.2016.0218 28443568 PMC5617890

[103] Wagner HE, Barbier P, Frey HP, Janggen FM, Rothen HU. Akutes Compartment-Syndrom nach Schlangenbiß. Der Chir. 1986;57(4):248–52. 3709299

[104] Styf J, Körner L. Acute compartment syndrome caused by snake bite. Lakartidningen. 1985;82(12):1059–60. 3990459

[105] Roed C, Lebech AM, Poulsen JB, Katzenstein T. Kompartmentsyndrom efter hugormebid. Ugeskr Laeger. 2009;171(5):327–8. 19176169

[106] Sennwald GG. Nécrose toxique sur morsure de Naja nigricollig, un défi thérapeutique. Schweiz Med Wochenschr. 1992;122(18):696–701. 1589745

[107] Bozkurt M, Kulahci Y, Zor F, Kapi E. The management of pit viper envenomation of the hand. Hand (N Y). 2008;3(4):324–31. doi: 10.1007/s11552-008-9114-2 18780013 PMC2584225

[108] Zimmerman J, Mann G, Kaplan HY, Sagher U. Envenoming by Cerastes viper - a report of two cases. Trans R Soc Trop Med Hyg. 1981;75(5):702–5. doi: 10.1016/0035-9203(81)90155-3 7330925

[109] Turchanyi BB. Kígyómarás okozta sérülések [Snake bite injuries]. Orvosi Hetil [Medical weekly]. 2000;141(20):1067–71. 10851889

[110] Lau YL, Kenna AP. Surgical treatment of adder bite. J R Soc Med. 1985;78(12):1028–30. doi: 10.1177/014107688507801210 4067976 PMC1290058

[111] Bernasconi L, Carnovale M, Lonati D, Brolli B, Negrini V, Grazioli C, et al. Compartment syndrome after Italian viper bite: two case reports. Clin Toxicol. 2023;61:35.

[112] Glatstein M, Lerman L, Gatt D, Scolnik D, Rimon A, Hoyte C, et al. Echis coloratus envenomation in children: a retrospective case series. Clin Toxicol. 2021;Jul 28:1–5. 10.1080/15563650.2021.1959604 34319210

[113] Aboud F, Assaf M, Shtarker H. A rare consequence of snakebite: A case report and literature review. Trauma Case Rep. 2025;57:101174. doi: 10.1016/j.tcr.2025.101174 40395366 PMC12090314

[114] Maffè S, Paffoni P, Facchini E, Bergamasco L, Prenna E, Ariotti S, et al. Venom-induced myocarditis: An unusual case attributable to Vipera aspis bite. Toxicon. 2024;250:108104. doi: 10.1016/j.toxicon.2024.108104 39303996

[115] Sassoè-Pognetto M, Cavalcante R, Paonessa M. Acute compartment syndrome and fasciotomy after a viper bite in Italy: a case report. Ital J Pediatr. 2024;50(1):70. doi: 10.1186/s13052-024-01638-5 38627836 PMC11020867

[116] Bouziri A, Khaldi A, Hamdi A, Belhadj S, Borgi A, Menif K, et al. Etat de choc, syndrome de détresse respiratoire aigue et syndrome des loges, causés par une morsure de vipère. La Tunesie Medicale. 2011;89(2):217–8. 21308641

[117] Dhar D. Compartment Syndrome Following Snake Bite. Oman Med J. 2015;30(2):e082. doi: 10.5001/omj.2015.32 30834067 PMC6387663

[118] Hachimi K, Fnini S, El Andaloussi Y, Trafeh M. Envenimations par morsure de serpents et syndrome de loge: À propos de deux observations. Chir Main. 2005;24(3–4):184–6. 16121627 10.1016/j.main.2005.06.005

[119] Hamdi MF, Baccari S, Daghfous M, Tarhouni L. Upper limb compartment syndrome after an adder bite: a case report. Chin J Traumatol. 2010;13(2):117–9. 20356449

[120] Al-Hashaykeh N, Al Jundi A, Abuhasna S. Delayed administration of antivenin three days after snakebite saves a life. Anaesthesia, Pain Intensive Care. 2011;15(3):167–9.

[121] Kazemi SM, Al-Sabi A, Long C, Shoulkamy MI, El-Aziz TMA. Case report: Recent Case Reports of Levant Blunt-Nosed Viper Macrovipera lebetina obtusa Snakebites in Iran. Am J Trop Med Hyg. 2021;104(5):1870–6. 33819174 10.4269/ajtmh.20-1640 PMC8103458

[122] Brimo AMZ, Toutounji Z, Arab R, Al Sam SO, Alhaj AMA, Kenno MF, et al. Successful management of late stage of acute compartment syndrome after 72 h snake bite in 8-year-old female. A case report. Trauma Case Reports. 2023;47:100922 37663375 10.1016/j.tcr.2023.100922 PMC10471994

[123] Navaeifar MR, Zakariaei Z, Ghadiri A, Soleymani M, Zakariaei A. Compartment syndrome following snakebite in a boy: A case report and literature review. Int J Surg Case Rep. 2023;105:108050. doi: 10.1016/j.ijscr.2023.108050 36989626 PMC10074563

[124] Pin L, Lutao X, Weijun F, Feng P, Chaohui C, Jianrong W. Ulnar artery pseudoaneurysm and compartment syndrome formation after snake bite to the left forearm. Clin Toxicol (Phila). 2018;56(6):436–8. doi: 10.1080/15563650.2017.1400042 29124975

[125] Okamoto O, Nakashima R, Yamamoto S, Hashimoto T, Takasaki T, Tokuda H, et al. A lethal case of mamushi (Gloydius blomhoffii) bite: severe bowel symptoms as a lethal sign. Acute Med Surg. 2016;4(1):135–9. doi: 10.1002/ams2.240 29123851 PMC5667284

[126] Hsu KY, Shih HN, Chen LM, Shih CH. Lower Extremity Compartmental Syndrome Following Snake-Bite Envenomation- One Case Report. Chang Gung Med J. 1989;13:54–8. 2379106

[127] Sugamata A, Yoshizawa N, Okada T. Relaxation incisions of venomous snake “Japanese mamushi” bites to the hand. Int Med Case Rep J. 2011;4:87–91. doi: 10.2147/IMCRJ.S27711 23754913 PMC3658245

[128] Singline P, Bush B. Controversial Australian Snakebite Treatment: The Deepest Cut of All. Venoms and Toxins. 2023;3(1):e090823219595

[129] Little M. Harm due to the use of pressure bandage immobilisation in patients bitten by snakes in Australia. Clin Toxicol (Phila). 2023;61(8):611–2. doi: 10.1080/15563650.2023.2252586 37668172

[130] Gakkai NH, Matori S. Early Onset of Acute Compartment Syndrome Diagnosed by a Simple Needle Manometer Technique. Nishinihon J Dermatology. 2013;76(5):454–8.

[131] Kawamura S, Kiyota K, Inagawa H, Terada T, Nishida M, Suzuki S, et al. A Severe envenomation from the bite of agkistrodon blomhoffii brevicaudus imported from China. The SNAKE. 2002;29.

[132] Chen C, Shen C, Peng S, Zou Y. Severe compartment syndrome following snakebite in a man: A case report. Asian J Surg. 2024;:S1015-9584(24)01926-2. doi: 10.1016/j.asjsur.2024.08.186 39237419

[133] Garfin SR, Castilonia RR, Mubarak SJ, Hargens AR, Russell FE, Akeson WH. Rattlesnake bites and surgical decompression: results using a laboratory model. Toxicon. 1984;22(2):177–82. doi: 10.1016/0041-0101(84)90018-7 6729838

[134] Bush SP, Green SM, Laack TA, Hayes WK, Cardwell MD, Tanen DA. Pressure immobilization delays mortality and increases intracompartmental pressure after artificial intramuscular rattlesnake envenomation in a porcine model. Ann Emerg Med. 2004;44(6):599–604. doi: 10.1016/j.annemergmed.2004.06.007 15573035

[135] Stewart RM, Page CP, Schwesinger WH, McCarter R, Martinez J, Aust JB. Antivenin and fasciotomy/debridement in the treatment of the severe rattlesnake bite. Am J Surg. 1989;158(6):543–7. doi: 10.1016/0002-9610(89)90188-8 2589586

[136] Tanen DA, Danish DC, Clark RF. Crotalidae polyvalent immune Fab antivenom limits the decrease in perfusion pressure of the anterior leg compartment in a porcine crotaline envenomation model. Ann Emerg Med. 2003;41(3):384–90. doi: 10.1067/mem.2003.80 12605206

[137] Garfin SR, Castilonia RR, Mubarak SJ, Hargens AR, Akeson WH, Russell FE. Role of surgical decompression in treatment of rattlesnake bites. Surg Forum. 1979;30:502–4. 538678

[138] Grace TG, Omer GE. The management of upper extremity pit viper wounds. J Hand Surg Am. 1980;5(2):168–77. doi: 10.1016/s0363-5023(80)80149-3 7358958

[139] Mars M, Hadley GP, Aitchison JM. Direct intracompartmental pressure measurement in the management of snakebites in children. S Afr Med J. 1991;80(5):227–8. 1887348

[140] Türkmen A, Temel M. Algorithmic approach to the prevention of unnecessary fasciotomy in extremity snake bite. Injury. 2016;47(12):2822–7. doi: 10.1016/j.injury.2016.10.023 27810154

[141] Wood D, Sartorius B, Hift R. Ultrasound findings in 42 patients with cytotoxic tissue damage following bites by South African snakes. Emerg Med J. 2016;33(7):477–81. doi: 10.1136/emermed-2015-205279 27068867

[142] Garfin SR, Mubarak SJ, Davidson TM. Rattlesnake bites: Current Concepts. Clin Orthop Relat Res. 1979;140:50–7. 477087

[143] Essafti M, Fajri M, Rahmani C, Abdelaziz S, Mouaffak Y, Younous S. Snakebite envenomation in children: An ongoing burden in Morocco. Ann Med Surg (Lond). 2022;77:103574. doi: 10.1016/j.amsu.2022.103574 35399368 PMC8987801

[144] Toffano LL, da Silva LO, Neves FdF, Teixeira LdAS, Silva-Vergara ML. Compartment Syndrome Secondary to Bothrops spp. Envenomation in Triângulo Mineiro, Region, Minas Gerais, Brazil. Rev Soc Bras Med Trop. 2023;56(March):e0130–023. 10.1590/0037-8682-0130-2023 PMC10367216 37493738

[145] Xian X, Jiang Z, Ren Y, Tang S, Liu Y, Bai T, et al. Limb circumference measurements contributing to the diagnosis of snake venom-induced compartment syndrome. Heliyon. 2024;10(17):e37057. doi: 10.1016/j.heliyon.2024.e37057 39286135 PMC11402951

[146] Downey DJ, Omer GE, Moneim MS. New Mexico rattlesnake bites: demographic review and guidelines for treatment. J Trauma. 1991;31(10):1380–6. 1942147

[147] Hsu C-P, Chuang J-F, Hsu Y-P, Wang S-Y, Fu C-Y, Yuan K-C, et al. Predictors of the development of post-snakebite compartment syndrome. Scand J Trauma Resusc Emerg Med. 2015;23:97. doi: 10.1186/s13049-015-0179-y 26561300 PMC4642665

[148] Kim YH, Choi J-H, Kim J, Chung YK. Fasciotomy in compartment syndrome from snakebite. Arch Plast Surg. 2019;46(1):69–74. doi: 10.5999/aps.2018.00577 30685944 PMC6369054

[149] El Koraichi A, Tsala G, Ahid S, Tadili J, Kharraz H, Zinelabidine E, et al. Le syndrome des loges au décours des envenimations vipérines de l’enfant. Reanimation. 2011;20(5):463–6.

[150] Mars M, Hadley GP. Failure of pulse oximetry in the assessment of raised limb intracompartmental pressure. Injury. 1994;25(6):379–81. doi: 10.1016/0020-1383(94)90130-9 8045642

[151] Harrison RA, Hargreaves A, Wagstaff SC, Faragher B, Lalloo DG. Snake envenoming: a disease of poverty. PLoS Negl Trop Dis. 2009;3(12):e569. doi: 10.1371/journal.pntd.0000569 20027216 PMC2791200

[152] Chippaux JP. Snake-bites: appraisal of the global situation. Bull World Health Organ. 1998;76(5):515–24. 9868843 PMC2305789

[153] GBD 2019 Snakebite Envenomation Collaborators. Global mortality of snakebite envenoming between 1990 and 2019. Nat Commun. 2022 Oct 25;13(1):6160. 36284094 10.1038/s41467-022-33627-9 PMC9596405

[154] Habib AG. Public health aspects of snakebite care in West Africa: perspectives from Nigeria. J Venom Anim Toxins Incl Trop Dis. 2013;19(1):27. doi: 10.1186/1678-9199-19-27 24134780 PMC3831819

[155] Alcoba G, Potet J, Vatrinet R, Singh S, Nanclares C, Kruse A, et al. Snakebite envenoming in humanitarian crises and migration: A scoping review and the Médecins Sans Frontières experience. Toxicon X. 2021;13:100089. doi: 10.1016/j.toxcx.2021.100089 35005609 PMC8718667

[156] Said M, Valdespino E, Baba SP, Lako R, Malm A, Gonzalez A, et al. Perspectives from MSF snakebite programme implementation in Agok, Abyei region, South Sudan. South Sudan Med J. 2020;13(4):146–52.

[157] Steinhorst J, Baker C, Padidar S, Litschka-Koen T, Ngwenya E, Mmema L, et al. Developing and applying a training needs analysis tool for healthcare workers managing snakebite envenoming: A cross-sectional study in Eswatini. PLoS Negl Trop Dis. 2025;19(1):e0012778. doi: 10.1371/journal.pntd.0012778 39776319 PMC11709266

[158] Anane-Binfoh NA, Flaherty KE, Zakariah AN, Nelson EJ, Becker TK, Afaa TJ. Barriers to Decolonizing Global Health: Identification of Research Challenges Facing Investigators Residing in Low- and Middle-Income Countries. Glob Health Sci Pract. 2024;12(1):e2300269. doi: 10.9745/GHSP-D-23-00269 38242635 PMC10906550

[159] Gutiérrez JM, Calvete JJ, Habib AG, Harrison RA, Williams DJ, Warrell DA. Snakebite envenoming. Nat Rev Dis Primers. 2017;3:17079. doi: 10.1038/nrdp.2017.79 28980622

[160] Steinhorst J, Litschka-Koen T, Ascenção B, Mmema L, Shongwe N, Murray J, et al. Symptoms and Management of Painful Progressive Swelling in Eswatini Snakebite Patients: A Prospective Observational Study. Am J Trop Med Hyg. 2025;112(6):1345–54. doi: 10.4269/ajtmh.24-0671 40233729 PMC12139540

[161] Pattinson JP, Kong VY, Bruce JL, Oosthuizen GV, Bekker W, Laing GL, et al. Defining the need for surgical intervention following a snakebite still relies heavily on clinical assessment: The experience in Pietermaritzburg, South Africa. S Afr Med J. 2017;107(12):1082–5. doi: 10.7196/SAMJ.2017.v107i12.12628 29262961

[162] Tilbury CR. Observations on the bite of the Mozambique spitting cobra (Naja mossambica mossambica). S Afr Med J. 1982;61(9):308–13. 7058469

[163] Mao YC, Liu PY, Chiang LC, Lai CS, Lai KL, Ho CH, et al. Naja atra snakebite in Taiwan. Clin Toxicol (Phila). 2018 Apr;56(4):273–80. 28830248 10.1080/15563650.2017.1366502

[164] African Snakebite Institute. Teeth and Fangs. https://www.africansnakebiteinstitute.com/articles/teeth-and-fangs-2/?srsltid=AfmBOopSR75F8-_H8q_fa3hhyT2sWTgs7leIiarQI753xL7GC7diNAQv

[165] Cleuren SGC, Rule JP, Ksas R, Herrel A, Hocking DP, Evans AR. Kinematics of strikes in venomous snakes. J Exp Biol. 2025;228(20):jeb250347. doi: 10.1242/jeb.250347 41128666 PMC12582407

[166] Bittenbinder MA, van Thiel J, Cardoso FC, Casewell NR, Gutiérrez J-M, Kool J, et al. Tissue damaging toxins in snake venoms: mechanisms of action, pathophysiology and treatment strategies. Commun Biol. 2024;7(1):358. doi: 10.1038/s42003-024-06019-6 38519650 PMC10960010

[167] Warrell D. Venomous and Poisonous Animals. Manson’s Tropical Diseases (24th Edition). Elsevier Limited; 2023;1099–1023.

[168] McQueen MM, Duckworth AD, Aitken SA, Court-Brown CM. The Estimated Sensitivity and Specificity of Compartment Pressure Monitoring for Acute Compartment Syndrome. The Journal of Bone and Joint Surgery. 2013;95(8):673–7. doi: 10.2106/jbjs.k.01731 23595064

[169] Whitesides TE, Haney TC, Morimoto K, Harada H. Tissue pressure measurements as a determinant for the need of fasciotomy. Clin Orthop Relat Res. 1975;(113):43–51. doi: 10.1097/00003086-197511000-00007 1192674

[170] Musculoskeletal Key. Compartment Syndromes. https://musculoskeletalkey.com/compartment-syndromes-2/?utm_source=chatgpt.com

[171] Courtney MT, Beauchamp RD, Evers BM, Mattox KL. Sabiston Textbook of Surgery- The Biological Basis of Modern Surgical Practice. 21st ed. Elsevier; 2021. p. 2176.

[172] Uliasz A, Ishida JT, Fleming JK, Yamamoto LG. Comparing the methods of measuring compartment pressures in acute compartment syndrome. Am J Emerg Med. 2003;21(2):143–5. doi: 10.1053/ajem.2003.50035 12671817

[173] Montreuil J, Corban J, Reindl R, Harvey EJ, Bernstein M. Novel digital continuous sensor for monitoring of compartment pressure: a case report. OTA Int. 2022;5(3):e208. doi: 10.1097/OI9.0000000000000208 36425093 PMC9580258

[174] Wong JC, Vosbikian MM, Dwyer JM, Ilyas AM. Accuracy of measurement of hand compartment pressures: a cadaveric study. J Hand Surg Am. 2015;40(4):701–6. doi: 10.1016/j.jhsa.2014.12.003 25648783

[175] Reichman EF. Compartment Syndrome of the Hand: A Little Thought about Diagnosis. Case Rep Emerg Med. 2016;2016:2907067. doi: 10.1155/2016/2907067 27293917 PMC4880689

[176] National Library of Medicine (ClinicalTrials.gov). ACS Monitoring Charité Berlin. 2024 https://clinicaltrials.gov/study/NCT06030635?cond=CompartmentSyndrome&rank=3

[177] National Library of Medicine (ClinicalTrials.gov). SWISS_CLEARANCE- Compartment Compressibility Monitoring Using CPM#1. 2022 https://clinicaltrials.gov/study/NCT05483946?cond=CompartmentSyndrome&rank=4

[178] Casewell NR, Wagstaff SC, Wüster W, Cook DAN, Bolton FMS, King SI, et al. Medically important differences in snake venom composition are dictated by distinct postgenomic mechanisms. Proc Natl Acad Sci U S A. 2014;111(25):9205–10. doi: 10.1073/pnas.1405484111 24927555 PMC4078820

[179] Bartlett KE, Hall SR, Rasmussen SA, Crittenden E, Dawson CA, Albulescu L-O, et al. Dermonecrosis caused by a spitting cobra snakebite results from toxin potentiation and is prevented by the repurposed drug varespladib. Proc Natl Acad Sci U S A. 2024;121(19):e2315597121. doi: 10.1073/pnas.2315597121 38687786 PMC11087757

[180] Ruha A-M, Kleinschmidt KC, Greene S, Spyres MB, Brent J, Wax P, et al. The Epidemiology, Clinical Course, and Management of Snakebites in the North American Snakebite Registry. J Med Toxicol. 2017;13(4):309–20. doi: 10.1007/s13181-017-0633-5 28975491 PMC5711762

[181] Varney S, Watkins S, Stuteville H, Gao T, Roth B, Alindogan A, et al. Fasciotomy after North American pit viper envenomation in 2004-2021. Clin Toxicol. 2023;61:132–3. 10.1080/15563650.2024.2338559 38804837

[182] Gagnier JJ, Kienle G, Altman DG, Moher D, Sox H, Riley D, et al. The CARE guidelines: consensus-based clinical case reporting guideline development. J Med Case Rep. 2013;7:223. doi: 10.1186/1752-1947-7-223 24228906 PMC3844611

