# Supplementary material for: Under pressure: Clinical management of venom-induced compartment syndrome in snakebite–A scoping review of the global literature
Source: PLoS Negl Trop Dis. 2026 Jul 31;20(7):e0014536. doi: 10.1371/journal.pntd.0014536 (PMC13427016; doi:10.1371/journal.pntd.0014536)
Supplement: S2 File — (PDF) [file pntd.0014536.s002.pdf]

Supplementary Material S2: Search string for literature search

PubMed (NLM)

("Snake Bites"[Mesh] OR "Snakes"[Mesh] OR "Snake Venoms"[Mesh] OR snake*[tiab] OR elapid*[tiab] OR viper*[tiab] OR colubrid*[tiab] OR bungarus[tiab] OR crotal*[tiab] OR bitis [tiab] OR adder*[tiab] OR naja [tiab] OR cobra* [tiab] OR bothrops [tiab] OR dendroaspis [tiab] OR mamba* [tiab] OR copperhead* [tiab] OR cottonmouth* [tiab] OR rattlesnake*[tiab] OR russelii [tiab] OR hydrophidae [tiab] OR Micrurus [tiab] OR haemachatus [tiab] OR hemachatus[tiab] OR ringhals [tiab] OR krait*[tiab] OR laticauda semifasciata[tiab] OR ophiophagus[tiab])

AND

("Compartment Syndromes"[Mesh] OR compartment*[tiab] OR painful progressive swelling [tiab] OR intracompartmental pressure* [tiab] OR fasciotom*[tiab])

Embase

(‘snakebite’/exp OR ‘snake venom*’/exp OR ‘snake*’/exp OR (‘snakebite*’ OR ‘snake*’ OR ‘elapid*’ OR ‘viper*’ OR ‘colubrid*’ OR ‘sea snake*’ OR ‘bungarus’ OR ‘crotal*’ OR ‘bitis’ OR ‘adder*’ OR ‘naja’ OR ‘ophiophagus’ OR ‘cobra*’ OR ‘bothrops’ OR ‘dendroaspis’ OR ‘mamba*’ OR ‘copperhead*’ OR ‘cottonmouth*’ OR ‘rattlesnake*’ OR ‘russelii’ OR ‘hydrophidae’ OR ‘hemachatus’ OR haemachatus OR ‘laticauda semifasciata’ OR ‘micrurus’ OR ringhals OR krait*):ab,ti)

AND

(‘compartment syndrome’/exp OR ‘intracompartmental pressure monitor’/exp OR (‘compartment*’ OR ‘painful progressive swelling’ OR ‘intracompartmental pressure*’ OR ‘fasciotom*’):ab,ti)

Cochrane

(snake* OR elapid* OR viper* OR colubrid* OR bungarus OR crotal* OR bitis OR adder* OR naja OR cobra* OR bothrops OR dendroaspis OR mamba* OR copperhead* OR cottonmouth* OR rattlesnake* OR russelii OR hydrophidae OR Micrurus OR haemachatus OR hemachatus OR ringhals OR krait* OR “laticauda semifasciata” OR ophiophagus)

AND

(compartment* OR “painful progressive swelling” OR “intracompartmental pressure*” OR fasciotom*)
