# Supplementary material for: Under pressure: Clinical management of venom-induced compartment syndrome in snakebite–A scoping review of the global literature
Source: PLoS Negl Trop Dis. 2026 Jul 31;20(7):e0014536. doi: 10.1371/journal.pntd.0014536 (PMC13427016; doi:10.1371/journal.pntd.0014536)
Supplement: S3 File — (DOCX) [file pntd.0014536.s003.docx]

**Supplementary Material S3: Summary of cases (n=115) with reported snake venom-induced compartment syndrome**

| Case report | Demographics | | Diagnostics | | | | Treatment | | |
| --- | --- | --- | --- | --- | --- | --- | --- | --- | --- |
| Author, year, country & case number (if multiple cases reported) | **Age, sex, snake species, captive snake ^1^, time bite-hospital** | **Anatomic site of the bite** | **Compartment syndrome** | | | **Coagulation at admission/**  **pre-op ^3^** | **Timing (relative to time of the bite), quantity of AV administered, non-surgical treatments provided, & response of local symptoms to treatment (*)** | **Timing of surgical treatment in hours after the bite ^2^, type of procedure (intra-operative findings), clinical signs, & symptoms post-op (*)** | **Maximum follow-up time ^5^, clinical**  **outcome ^6^, & complications** |
|  |  |  | **Symptoms & clinical exam** | **Diagnostics** | **Time of diagnosis (after bite) ^2^** |  |  |  |  |
| Americas | | | | | | | | | |
| Thomas et al. (2014) (41)  Trinidad and Tobago | 16  M  *Bothrops athrox*  20min | Dorsum of hand | Swelling (twice volume of healthy limb)  Pain (VAS=10/10)  Paraesthesia  Muscular paralysis  Cyanosis | Clinical only | 2h 45min | PT: 16.2s  aPTT: 24s  Coagulopathy ^4^ | No AV administered  Analgesic medication | 5h 45min  1 Fasciotomy (oedematous  & ischaemic muscle  tissue, no necrosis)  1 Wound closure  procedure  1 Wound closure  procedure  *Reduced pain, recovery  of motion following  fasciotomy | 6 months: No sequelae |
| Gold et al. (2003) (28)  USA | 43  M  *Crotalus atrox*  Captive snake (intentional)  2h | Thenar eminence of hand | Swelling (marked)  Pain  Pain on passive stretch  Paraesthesia  Hypoesthesia  Arterial pulsations intact  CR: normal | ICP 55 mmHg in thenar eminence 2h after bite | 2h 30min | Not reported | AV administered  2h 30min: 4 vials ^4^  2h30min–  5h 30min: 20 vials  8h 30min: 10 vials  Total AV: 34 vials  Meperidine  Morphine  Mannitol infusion  Hyperbaric O_2_ therapy  *ICP 30–32 mmHg at 8h 30 min after the bite | No operation performed (fasciotomy recommended by surgeon, but refused by patient) | Discharge (3d): No sequelae  Adverse reaction to AV (serum sickness) |
| Brys et al. (2015) (42)  USA | 9  M  *Agkistrodon contortrix*  1h 30min | Thenar eminence of hand | Swelling (dense, ext. to upper arm)  Tenderness  Ecchymosis | ICP 56 mmHg in thenar, 55 mmHg in palmar, 32 mmHg in dorsal interosseous of index finger & 15 mmHg in volar forearm compartments | 1d | Not reported | AV administered  1h 30min: 6 vials  12h: 4 vials  18h: 4 vials  21h: 2 vials  Pre-op: 4 vials  Total AV: 20 vials  Limb elevation  *Persistence of elevated ICP, in thenar (55 mmHg) & dorsal interosseous compartment (33mmHg) as measured pre-op | 1d  1 Fasciotomy (grey-looking  intrinsic muscles of the  hand) | 2 weeks: No sequelae |
| Bucaretchi et al. (2010) (43)  Brazil | 39  M  *Bothrops jararaca*  5h | Lower leg | Swelling (tense)  Pain (VAS= 8–9/10)  Pain on passive stretch  Hypoesthesia  Limited dorsiflexion of foot  Ecchymosis  Gingival haemorrhage | ICP 60 mmHg in anterior compartment 8h after bite  MRI^9^ of lower leg 48h after bite: loss of muscle architecture, muscle oedema & haemorrhage in anterior compartment | 2d 6h | PT: Incoagulable aPTT: Incoagulable  INR: Incoagulable  Plt: 12  Coagulopathy  Thrombocytopenia ^4^ | AV administered  6h: 8 vials  2d 9h (pre-op): 4 vials  Total AV: 12 vials  Antibiotics  Tramadol  Paracetamol  Codeine  Glucocorticoids  Diphenhydramine  *Progressive decrease in ICP to 36 mmHg 19h after bite, reduction of pain (VAS=  2–4/10), increase in platelet count (190), improvement of INR (1.51). Persistence of pain, limited dorsiflexion. | 2d 9h:  1 Fasciotomy (necrosis &  haemorrhage in medial &  distal segments of  anterior tibial muscle)  ≥ 2 serial debridements  1 Wound closure  procedure | 74d: Sequelae (permanent fibular palsy)  Local wound infection  Adverse reaction to AV (urticaria & flushing) |
| Bucaretchi et al. (2014) (39)  Brazil | 63  M  *Crotalus durissus terrificus*  1h | Elbow | Swelling (tense)  Pain (disproportionate, VAS= 10/10)  Pain on passive stretch  Paraesthesia  CR: < 2s  Erythema | ICP 40 mmHg & ΔP^8^ 97 mmHg in anterior compartment 25h after bite  MRI 24h after bite showed muscular oedema | 1d 1h | PT: >120s aPTT: >180s  INR: >11 Plt: 238  Coagulopathy | AV administered  1h: 8 vials  15h: 20 vials  Total AV: 28 vials  Morphine  IV fluids  Urine alkalization Nitroprusside infusion  Amlodipine  Limb elevation  *Improvement of VAS score (7/10), persistent coagulopathy at 13–14h after bite, evolving muscle weakness & neuro-myotoxicity, palpebral ptosis, general myalgia, severe rhabdomyolysis. | No operation performed | Discharge (6d): No sequelae |
| Hardy et al. (2006) (44)  USA | 35  F  *Crotalus molossus*  1h 9min | Anterior aspect of lower leg | Swelling (ext. to inguinal region)  Pain (significant)  Hypoesthesia  Muscular paralysis  Ecchymosis | ICP 68 mmHg in anterior compartment (max pressure recordable by device) & 25 mmHg in lateral compartment 54h after bite  Circumference measurements (no details) | 54h | aPTT: 12.6s  Plt: 229  Coagulation tests normal | AV administered  2h 9min: 4 vials  7h 39min: 4 vials  15h: 2 vials  21h: 2 vials  Total AV: 12 vials  Morphine | 56h  1 Fasciotomy (substantial  amounts of necrotic  muscle)  1 Re-exploration &  debridement of wound  (central 2/3 of anterior  compartment muscles  nonviable)  1 Debridement of infection  1 Abscess drainage  1 Adhesiolysis | 24 months: Sequelae (peroneal nerve damage, no recovery of motor function & sensory function, inability to dorsiflex foot beyond neutral position). Despite disability, patient was able to perform long-distance running.  Local wound infection |
| Mendez-Dominguez et al. (2019) (45)  Mexico | 40  M  *Bothrops asper*  5h | Dorsal popliteal fossa of leg | Swelling  Ecchymoses  Haemorrhage from bite site & head trauma (due to fall) | Clinical only | >8h | Coagulopathy  DIC | AV administered  5h: 15 vials  Total AV: 15 vials  Outside hospital:  Tourniquet  Inside hospital:  Antibiotics  Steroids  Haemodialysis  Bandages  Platelets  *Progression of oedema in affected leg. | 1 Fasciotomy | 3d: Patient deceased (digestive tract haemorrhage, renal failure, multiple organ failure & cranial lesions from head trauma through fall) |
| McBride et al. (2017) (46)  USA | 48  M  *Crotalus adamanteus* | Lower leg | Swelling (ext. to thigh)  Tenderness | ICP 42 mmHg in anterior & 72 mmHg in lateral compartment | Not reported | PT: >120s  PTT: >200s INR: incalculable plt=125  Coagulopathy | AV administered  Pre-op: 12 vials  Post-op (over 48h): 18 vials  Day 6 (& later): 24 vials  Total AV: 54 vials  VAC wound closure (post-op)  pRBCs (8 units)  Platelets (3 super packs)  FFP (14 units)  Cryoprecipitate (4 units)  PCC (2 doses)  *Coagulopathy improved (INR =1.29, PTT=26.4sec) | 1 Fasciotomy  1 Wound closure  1 Re-exploration of  haemorrhaging leg wound | Discharge (15d): Limb salvage  Coagulopathy & haemorrhage from fasciotomy wound continuing post-operatively (1000cc of blood in VAC drainage system) |
| Valente-Aguiar at al. (2019) (47)  Brazil | 28  M  *Bothrops jararaca*  5d  Discharged after initial presentation to hospital; returned 5 days after the bite (this presentation) | Lateral aspect of middle third of leg | Swelling  Pain (severe)  Ecchymosis  Pallor  Myonecrosis  Bullous erythema  Regional lymphadenopathy | Clinical only | Not reported | Plt: 54.3  Thrombocytopenia | AV administered  5d: 30 vials  Total AV given: 30 vials  Analgesia  Imipenem  Ampicillin Sulbactam  Cilastatin  Vancomycin | ≥5d  1 Fasciotomy | Discharge (71d): Limb salvage |
| Shaw et al. (2002) (29)  USA | 9  M  *Crotalus atrox* | Leg | Swelling (ext. across entire leg)  Pain on passive movement  Hypoesthesia (in toes)  Ecchymosis | ICP 45 mmHg in anterior & posterior compartment | 8h | Not reported | AV administered  6h: AV administered  *Adverse reaction to AV & further AV withheld. Persisting pain, pain on passive stretch, tense swelling of entire leg, hypoesthesia of toes. | 8h  1 Fasciotomy | Discharge: No sequelae  Adverse reaction to AV (urticaria) |
| Sachett et al. (2020) (19)  Brazil | 27  M  *Bothrops atrox*  2d | Lower limb | Not reported | Clinical only | Not reported | PT: 20s  PTT: 49s  INR: 1.7  Plt: 67  Coagulopathy  Thrombocytopenia | AV administered  2d: 12 vials  Total AV: 12 vials  Outside hospital:  Washing of wound with soap & water  Herbal mixture taken orally  Inside hospital:  Endotracheal intubation  ICU admission  Vancomycin  Imipenem  Fluconazole  Noradrenaline  Dobutamine  *Coma after administration of AV. Declining health status, progression to CS. | 2d  1 Fasciotomy  1 Debridement (for  extensive necrosis at  fasciotomy site)  3 Skin grafting &  debridement procedures | 4 months: Sequalae (low scores on WHO Disability Assessment Schedule in cognition domain (35%), mobility domain (55%), self-care domain (35%), getting along domain (10%), impact on life activities (88%) & participating in society domain (63%))  Transient acute kidney injury (KDIGO stage 3) |
| Resiere et al. (2016) (48)  Martinique | 64  M  *Bothrops lanceolatus*  1h 5min | Forearm | Swelling (extensive)  Pain  Ecchymosis  Blistering (forearm & hand palm) | ICP measured but not reported | 15h 20min | Coagulation tests (aPTT, PT, INR) all within normal ranges | AV administered  3h 20min: 30 mL  Day 3: 20mL  Day 5: 20mL  Total AV: 80mL    Outside hospital:  Tourniquet  Inside hospital:  Haemodialysis started (in jugular vein) with unfractionated heparin to treat hyperkalaemia  Gentamicin  Ceftriaxone  Metronidazole  Insulin | >15h  1 Fasciotomy  1 Thrombectomy (day 15)  *Post-op rapid improvement of hand function, but several hours after operation paraesthesia of second & third finger developed | Discharge (17d): Limb salvage with sequelae (sensory deficit in second & third finger)  Thrombosis of arteriovenous fistula (authors believe this was related to the fasciotomy) |
| Rosen et al.  (2000) (49)  USA | 59  M  *Crotalus atrox* | Dorsum of foot | Swelling (ext. to thigh & scrotum)  Pain on passive movement  Tenderness  Hypoesthesia  No paralysis  Arterial pulsations decreased  Ecchymosis  No pallor | ICP 46 mmHg in anterior tibial compartment & 33 mmHg in lateral tibial compartment | 2d | PT: 12.2s  INR: 1.06  Plt: 84  After transfer to tertiary facility:  PT: 14.4s  INR: 1.61  plt: 8  Coagulopathy  Thrombocytopenia | AV administered  First hospital: 5mL (test-dose)  Day 2: 15 vials  Total AV: 15 vials + 5mL  Outside hospital:  Poultice of herbs & clay  Inside hospital:  Diphenhydramine Methylprednisolone  IV fluids  Morphine  Platelets (70U)  Cryoprecipitate (30U)  Elevation of leg above heart  *Improvement of coagulation (PT=13.5s, INR= 1.41). Plt increased to 40. ICP 17 mmHg in anterior tibial & 16 mmHg in lateral tibial compartment. Leg was softer & less painful, extremity decreased in size & neurovascular status returned to normal. | Patient declined fasciotomy; adverse AV reaction led patient to refuse further AV treatment until CS was diagnosed, which is when AV treatment was resumed | 2 weeks: Limb salvage with sequalae (persisting swelling in ankle & discomfort when walking, although functionality preserved)  Adverse reaction to AV (headache, nausea, flushing vomiting, diaphoresis, hypotension, loss of bowel & bladder control, unconsciousness  & atrial fibrillation, later serum sickness) |
| Mazer-Amirshahi et al. (2014) (30)  USA | 17 months  F  *Agkistrodon contortrix* | Dorsum of foot | Swelling (circumferential)  Pain limiting mobilization of foot  Sensation intact  Arterial pulsations palpable  CR: brisk  Ecchymosis (ext. to ankle)  Erythema | ICP 85 mmHg in anterior, 55 mmHg in lateral, 27 mmHg in posterior, 34 mmHg in deep posterior, 60 mmHg in foot & 30 mmHg quadriceps compartment  Distal arterial pulsations measurable by doppler | 15h | PT: 11.4s  aPTT: 24.4s  INR: 1.1  Plt: 310 | AV administered  2h: 4 vials  8h: 2 vials  12h: 6 vials  21h: 6 vials  26h: 6 vials  36h: 2 vials  Total AV: 26 vials  Analgesic medication  *In course of AV treatment, ICP reduced to 54 mmHg in anterior & 30 mmHg in lateral compartment. Reduction in swelling & limb circumference & clinical improvement. | No operation performed (in consultation with paediatric ICU team & poison centre decided to defer fasciotomy in favour of AV treatment) | 2 weeks: No sequelae |
| Thomas et al. (2011) (50)  USA  Case 1 | 8  F  *Crotalus lutosus* | Ankle | Swelling (foot & ankle)  Pain (severe)  Ecchymosis (ext. to lower leg)  Erythema | ICP 65–68 mmHg in lower leg & 28 mmHg in upper leg | Not reported | No coagulopathy | AV administered  Total AV: 32–38 vials | No operation performed | Discharge (6d): No sequelae |
| Thomas et al. (2011) (50)  USA  Case 2 | 2  M  *Crotalus lutosus* | Index finger | Swelling (ext. to arm)  Cyanosis | ICP 60 mmHg between first & second metacarpals | Not reported | PT: 18.8s  aPTT: 52s  INR: 1.6  Coagulopathy | AV administered  <8h: 4 vials  Total AV: 15 vials | 1 Fasciotomy | Discharge: No sequelae |
| Ruha et al. (2011) (51)  USA | 53  M  *Crotalus atrox* | Base of thumb | Anaesthesia (thumb tip)  Reduced skin temperature  Blistering | Initially ICP ‘normal’ in forearm, clinical deterioration afterwards | 36h | Not reported | AV administered  2h 30min: 8 vials AV  Total AV: 24 vials | >36h  1 Fasciotomy  >1 Incision & debridement  procedures, including  bone grafting | 16 weeks: Sequelae (amputation of digit/portion of digit) |
| Roberts et al. (1985) (52)  USA  Case 1 | 14  M  *Sistrurus miliarius*  3h | Proximal phalanx of thumb | Swelling (extreme & tense)  Pain on movement  Paraesthesia  Limited movement (by oedema)  Arterial pulsations palpable  CR: absent  Cyanosis (thumb) | ICP 60 mmHg in thenar, 30 mmHg in volar & 30 mmHg in dorsal forearm compartment | Not reported | Not reported | AV administered  Total AV: 7 vials  IV fluids | 6h  1 Fasciotomy (thenar  compartment muscles  ischemic, muscles bulged  widely through  fasciotomy incision. Less  ischaemia seen in  forearm. Carpal tunnel  excessively tight)  1 Wound closure  procedure | Discharge: No sequelae |
| Roberts et al. (1985) (52)  USA  Case 2 | 39  M  *Agkistrodon piscivorus*  45min | Metacarpal of thumb | Swelling (ext. to forearm)  Pain on passive movement  Paraesthesia  CR: insufficient  Poor circulation of the thumb  Cyanosis | ICP 60 mmHg in thenar & 40 mmHg in volar compartment | Not reported | Not reported | AV administered  Total AV: 10 vials  IV fluids  *Worsening of forearm swelling | 3h  1 Fasciotomy, including  carpal tunnel release  (distinct linear zone of  ischaemia in biceps  muscle & bulging muscle  through incision)  2 Wound closure  procedures  *Post-op ICP in dorsal compartment 26mmHg | Discharge: No sequelae |
| Oporta (2010)  (53)  Costa Rica  Case 1 | 36  M  *Bothrops asper*  2h | Finger | Swelling (tense, ext. to shoulder & hemithorax)  Pain (extreme)  Dermo-myonecrosis  Haemorrhage | Clinical only | Not reported | Likely  abnormal  coagulation, as was reported to be corrected prior to operation | Not reported  VAC wound closure (post-op) | 1 Fasciotomy  1 Wound closure  procedure  *Post-op drastic change in the evolution of the oedema & relief of pain | Discharge: Limb salvage |
| Oporta (2010)  (53)  Costa Rica  Case 2 | 10  F  *Bothrops asper*  1h | Lateral aspect of leg | Swelling (ext. to thigh)  Pain  Paraesthesia  Hyperesthesia  Absent tibial pulsations  Ischemic changes on the dorsum of the foot  Haemorrhage from puncture marks | Clinical only | Not reported | Not reported | AV administered  VAC wound closure (post-op) | 1 Fasciotomy (ischaemia of  tibial muscles of the  lateral compartment)  1 Debridement  1 Wound closure &  placement of split-skin  graft over foot  13 VAC changes in OR  under general  anaesthesia | Discharge: Limb salvage |
| Oporta (2010)  (53)  Costa Rica  Case 3 | 32  M  *Bothrops asper* | Hand | Swelling (significant, ext. to shoulder)  Arterial pulsations impalpable distally  Cyanosis | Clinical only | Not reported | Not reported | AV administered  Total AV: 10 vials  VAC wound closure (post-op) | 1 Fasciotomy  3 VAC placements  1 SSG procedure | Discharge: Limb salvage |
| Oporta (2010)  (53)  Costa Rica  Case 4 | 33  M  *Bothrops asper*  18h | Second finger/ hand | Swelling (ext. to thorax)  Pain (ample)  Paraesthesia | Clinical only | 18h | Not reported | AV administered | 1 Fasciotomy (no evidence  of muscle tissue damage)  1 Wound closure  procedure  2 VAC placements | Discharge (7d): Limb salvage |
| Campbell et al. (2008) (54)  USA  Case 1 | *2*  *Agkistrodon Piscivorous* | Hand | Not reported | ICP 60 mmHg prior to fasciotomy | Not reported | Not reported | AV administered  Total AV: 2 doses | 20h  1 Fasciotomy | Discharge: Limb salvage |
| Campbell et al. (2008) (54)  USA  Case 2 | *2*  *Agkistrodon piscivorous* | Foot | Not reported | ICP 35 mmHg in leg & 40 mmHg in foot | Not reported | Not reported | No AV administered | 92h  1 Fasciotomy | Discharge: Limb salvage |
| Cavazos et al. (2023) (55)    USA | 56  M  *Crotalus Adamanteus*  Captive snake  <2h 50 min | Hand | Swelling (tense, ext. from hand to upper arm)  Bruising  Haemorrhage  * Not possible to assess  sensory & motor  function due to  intubation | ICP 50, 37 & 29 mmHg in forearm & 35–105 mmHg in hand  Arterial pulsations measurable by doppler | 11h 30min | Not reported | AV administered  2h 50min: 6 vials  5h 50min: 6 vials  11h: 10 vials  Total AV: 22 vials  Intubation | >11h 30min  1 Fasciotomy  3 Debridement &  irrigation procedures  1 Primary wound closure  1 Skin grafting procedure | 2 years: No sequelae  Adverse reaction to AV (anaphylactic reaction to snake venom/ antivenom requiring intubation) |
| Lazar et al. (2023) (56)  USA | 40  M  *Crotalus horridus*  Initial care at other hospital, 3.5h to referral hospital (this presentation) | Index finger | Swelling (ext. to mid forearm) | ICP 30 mmHg in thenar, 30 mmHg in hypothenar, 16 mmHg in dorsal compartment | Not reported | PT: 11.2s  Plt= 250 | AV administered  4h: 6 vials  After diagnosis of CS: 6 vials + 3 x 2 vials in 6-hour intervals  Total AV: 18 vials  *ICP reduced to 27 mmHg in thenar & 12 mmHg in hypothenar compartments. Improvement of pain & clinical exam. | No operation performed  *In consultation with toxicology team ICP was measured and after reduction on ICP on re-assessment fasciotomy was withheld. | Discharge (40h): Survival |
| Lizarzaburu-Ortiz et al. (2022) (57)  Ecuador | 26  M  *Bothrops asper*  Initial care at a local hospital, later referred (this presentation) | Dorsum of foot | Swelling (ext. over entire lower limb)  Pain (severe)  Arterial pulsations impalpable distally  Blistering  Cyanosis | Clinical only | Not reported | PT: 17.7 sec  Coagulopathy | AV administered  >8 vials  Clindamycin  Cefepime  Piperacilline/ Tazobactam | 1 Fasciotomy (necrotic  muscle seen)  1 Wound closure & repair  procedure  1 Escharotomy  1 Skin flap reconstruction  procedure | 3 months: No sequelae |
| Yakey et al. (2022) (58)  USA | 56  M  *Crotalus adamanteus* | Thumb | Swelling (ext. to elbow) | ICP >80 mmHg | >12h | Coagulopathy (patient used apixaban) | AV administered  Total AV: 20 vials  Intubation  Epinephrine  Antihistamines  Steroids  Platelets (1 unit) | >12h  1 Fasciotomy | Discharge (17d):  Limb salvage  Adverse reaction to AV (presumed anaphylaxis) |
| Lund et al. (2020) (59)  USA | 39  M  *Crotalus adamanteus*  Initial care at other hospital, 10h to referral hospital (this presentation) | Anterolateral aspect of lower leg | Swelling (significant)  Pain (debilitating)  Unable to bear weight | ICP 40–63 mmHg | >10h | INR: 1.14  Clotting parameters reported as ‘normal’ | AV administered  Total AV: 12 vials | >10h  1 Fasciotomy  1 Exploration of  fasciotomy wound  (myonecrosis of the  tibialis anterior,  peroneus longus &  brevis muscle observed) | Discharge (21d): Survival |
| Navarro-Vergara et al. (2025) (60)  Paraguay | 12  Female  *Bothrops jararaca*  3h | Finger | Not reported | Clinical only | **≤**9h | Not reported | No AV administered | 9h  1 Fasciotomy  2 Wash-out procedures of  wound  1 Wound closure  procedure | Discharge: Limb salvage  Renal failure (no details reported) |
| Africa | | | | | | | | | |
| Firth at al. (2016) (61)  South Africa | 1  M  *Bitis arietans*  <2d (arrival at referral hospital 2d after bite) | Arm/Hand | Swelling (ext. to axilla)  Necrotic skin | Clinical only | ≥2d (not reported) | Thrombocytopenia | AV administered  4d: AV administered  pRBCs  Platelets | <4d (before AV given)  1 Fasciotomy (tissues not  found to be typical of  compartment  syndrome)  ≥ 2 debridement  procedures  *Post-operatively, the skin & subcutaneous tissues became necrotic | 12d: Deceased due to sepsis |
| Gras et al. (2012) (62)  Central African Republic | 13  M  Snake unknown  6h | Arm/Hand | Swelling (ext. to back & shoulder)  Sensory & movement deficits  Absent circulation to extremity  Hard skin  Blanching of nails  Gingival haemorrhage & haemorrhagic syndrome | Clinical only | 1d | 20WBCT: positive  Coagulopathy | AV administered  6h: 1 dose  12h: 1 dose  Total AV: 3 doses  *After second dose of antivenom, oedema stopped progressing & clinical haemorrhage ceased. Presence of sensory, movement & circulatory deficits. | <24h  1 Fasciotomy (signs of CS  in hand compartment,  deep hematomas,  muscles were black with  almost no blood flow)  ≥7 serial debridement  procedures &  amputation of 2 fingers  *Necrosis eventually extended to the entire hand | 25d (the patient absconded on day 25 prior to skin grafting procedure & was lost to follow-up): Sequelae (amputation of two fingers) |
| Kouassi et al. (2017) (63)  Ivory Coast | 15  M  *Viperidae* | Dorsal aspect of hand | Swelling (ext. up to axilla)  Pain  Posture: flexion of wrist, hyperextension of MCP joints, flexion of proximal interphalangeal joints, flexion abduction of thumb  Blistering  Haemorrhage from bite site | Clinical only | Not reported | PT: 50% (of normal activity)  aPPT: 30s  Plt: 30  Coagulopathy  Thrombocytopenia | AV administered  14h: 2 vials  Total AV: 2 vials  Dexamethasone  IV fluids  Paracetamol,  Tetanus vaccination  RBCs  Amoxicillin & clavulanic acid | 1 Fasciotomy | 40d: Limb salvage with sequelae (scarring of the fasciotomy wound, functional deficits & stiffness in wrist & fingers) |
| Le Dantec et al. (2004) (64)  Senegal | 24  M  *Bitis arietans*  48h (referral) | Leg | Swelling  Pain  Tenderness  Blistering  Paleness  Haemorrhage from bite site | ICP 45 mmHg in three compartments (not specified) on day one; ICP continuously measured for seven days, max. 80 mmHg)  Ultrasound: no muscle necrosis seen & arterial pulsations in foot measurable by doppler | Not reported | PT: 35% (of normal activity)  aPTT: prolonged  Plt: 60  Coagulopathy  Thrombocytopenia | AV administered  >48h: 40mL  Total AV: 40mL  Cephazolin (prophylactically)  Tetanus vaccination  Morphine  IV fluids  O2 (15L/min)  Blood (3 units)  FFP (3 units)  Disinfection  Bandaging  *Within several hours after AV treatment hemodynamic situation improved. Haemorrhage subsided; the APTT, PT & platelet count improved. Systemic signs improved markedly, but oedema and pain worsened, and blisters increased in number and size. | 1 Debridement at site of  the bite | 6 weeks: Limb salvage with sequalae (scarring & granulation tissue over foot/leg)  Rhabdomyolysis (CPK 13 010 U/L)  Acute kidney injury (‘moderate’, serum creatinine: 120 mmol/L) |
| Blaylock (2004) (65)  South Africa | 11  M  *Bitis arietans* | Leg | Swelling (gross) | ICP 140 mmHg in anterior tibial, 110 mmHg in posterior calf & 190 mmHg in anterior thigh compartment | Not reported | Coagulopathy | No AV administered  IV fluids (5150mL in 24h)  pRBCs (1050mL)  FFP (1250mL) | 31h  1 Fasciotomy | 4d: Deceased due to uncontrolled coagulopathy, which was also not controlled at time of operation |
| Schweitzer et al. (1981) (66)  South Africa | 16  F  *Bitis arietans*  2h | 2 separate bites, one into index finger of left hand & one into right hand | Left index finger:  Swelling (ext. to forearm)  Pain on passive stretch of fingers  Tenderness  Hypoesthesia  Paraesthesia  Clawing of fingers & thumb; opposition of thumb impossible  Arterial pulsations in radial artery impalpable  CR: present  Dark blebs  Ecchymosis  Haemorrhaging fang marks  Right hand:  Swelling (right hand & wrist)  Sensation intact  Clawing of fingers & thumb  20 degrees of flexion & extension possible  Arterial pulsation in radial artery palpable  Clinical impression of raised ICP | Clinical only | Not reported | Not reported | AV administered  2h: AV administered  Hydrocortisone  Procaine  Penicillin  Mepyramine maleate Tetanus vaccination Elevation of the arm | 1 Fasciotomy & bilateral  carpal tunnel release in  left & right upper limbs  (Left hand: Clotted blood  underneath palmar  fascia. Oedematous  contents of volar muscle  compartment & carpal  tunnel. Nerve had  cyanotic appearance &  vessel on its volar surface  congested. Indentation  where the nerve passed  under the proximal edge  of the flexor retinaculum.  Right hand: Swelling in  hand & wrist. Clotted  blood deep under palmar  fascia in hand. Findings in  carpal tunnel identical to  other side.) | 35d: No sequelae  Local wound infection |
| Blaylock (2003) (67)  South Africa | 15  M  *Bitis arietans*  15h | Ankle | Swelling (ext. to chest wall)  Tenseness (extreme) of leg below the knee  Arterial pulsations in pedal arteries impalpable  Blistering | Absence of pedal arterial pulsations on doppler examination | Not reported | aPTT= 118s  INR= 2.45  Plt= 127  Coagulopathy | AV administered  3h 20min: 18mL  Total AV: 18mL  IV fluids (9L in first 24h)  4% albumin (1L)  Freeze-dried plasma (1.25L), pRBCs (2.5L)  Dextran 40 (0.5L)  20% Mannitol (1L) | 1 Fasciotomy, including  division of the inguinal  ligament (blood flow to  leg not restored until  inguinal ligament was  divided)  1 Debridement  1 SSG  *Loss of musculature in the anterior & peroneal compartment & skin on dorsum of the foot | 4 months: Limb salvage with sequelae (ambulatory, but with walking aid)  Adverse reaction to AV (anaphylaxis, hypotension) |
| South-East Asia | | | | | | | | | |
| Tangtermpong et al. (2021) (68)  Thailand | 39  M  *Protobothrops kelomohy*  4h | Ankle | Swelling  Pain on passive stretch Paraesthesia  Absent arterial pulsations  Non-haemorrhagic blebs | Leg circumference increased by 4.5cm compared to healthy leg | 18–19h | 20WBCT: positive  PT: 18s aPTT: 31s INR: 6  Plt: 157  Coagulopathy | AV administered  10h: 50ml  18h: 30ml  2d: 30ml every 7–12h until day 5  Total AV: 240ml  FFP | 19h  1 Fasciotomy  1 Wound closure  procedure | 3 months: No sequelae  Rhabdomyolysis (CPK 14 407 U/L; Creatinine 1.3 mg/dL) |
| Hon et al. (2005) (69)  Hong Kong | 6  M  *Trimeresurus albolabris*  30min | Lateral aspect of ankle | Swelling  Tenderness  Bruising | ICP 28 mmHg in anterior compartment 18h after bite  Circumference affected leg 21cm compared to 20cm on healthy side | 18h | PT: 12.5s aPTT: 40.2s  Plt: 137  Coagulopathy | AV administered  14h 30min: Two test doses of 0.1mL & 0.4mL  Total AV: 0.5mL  Paracetamol  Morphine  Adrenaline  IV Hydrocortisone Chlorpheniramine  Antibiotics  Tetanus vaccination  Corticosteroids  FFP  pRBCs  Platelets  *Worsening leg pain & swelling, extending to mid-thigh 12.5h after bite | 18h 30min  1 Fasciotomy  *Orthopaedic surgeon delayed fasciotomy in favour of AV because of falling platelet count (plt=24; 4h prior to operation, plt=18, PT= 12.5s, APTT=47.7s) | Discharge: Limb salvage  Adverse reaction to AV (anaphylactic shock, hypotension) |
| Panda et al. (2023) (70)  India | 26  F  *Naja naja*  60min to health centre, 4h to referral hospital (this presentation) | Dorsum of foot | Swelling (ext. to below knee)  Pain (severe)  Tenderness  Weak arterial pulsations in dorsalis pedis artery  Blistering  Local necrosis | ICP >30 mmHg (compartment not specified) | 7d | 20WBCT: negative | AV administered  60min: 10 vials  Total AV: 20 vials  Paracetamol  Ropivacaine (nerve block)  Antibiotics  Paracetamol  Anti-inflammatory medication  Cleaning of wound  *Progression of swelling, diminishing peripheral arterial pulsations | 7d  1 Fasciotomy  1 Reconstruction with  tissue flap  1 Caesarean section  performed (34^th^ week,  due to envenoming while  pregnant prior to  fasciotomy) | 6 weeks: No sequelae |
| Rathnayaka et al. (2022) (71)  Sri Lanka  Case 1 | 4  F  *Hypnale spp.* | Medial aspect of foot | Pain (severe)  CRT: prolonged | Drop in SpO2 | >13h 40 min | 20WBCT: negative  PT: 13s  aPTT: 31s  INR: 1.09  Plt: 301  Coagulopathy | No AV administered (no AV specific to *Hypnale spp.* exists)  Outside hospital:  Bite site washed with soap & tourniquet applied  *Progression of swelling, prolonging of CRT & reduction SpO_2_ | >13h 40 min  1 Fasciotomy  1 Skin grafting procedure | Discharge (18d): Survival |
| Rathnayaka et al. (2022) (71)  Sri Lanka  Case 2 | 3  M  *Hypnale spp.*  19h 35 min | Dorsum of hand | Pain (severe)  Weak arterial pulsations in radial artery  CRT: < 2s | Drop in SpO_2_ | >31h 30 min | 20WBCT: negative  PT: 11.8s  aPTT: 42.6s  INR: 0.98  Plt: 287  Coagulopathy | No AV administered (no AV specific to *Hypnale spp.* exists)  Elevation of arm  *Prolonging of CRT & diminishing SpO_2_ | >31h 30min  1 Fasciotomy  ≥1 Debridement (s)  1 Skin grafting procedure | Discharge (1d): Survival |
| Rathnayaka et al. (2022) (71)  Sri Lanka  Case 3 | 2,5  Male  *Hypnale spp.*  45min | Middle finger | Swelling (severe)  Pain (severe)  Haemorrhagic blister Necrosis  Lymphadenopathy | Drop in SpO_2_ | <24h | 20WBCT: negative  PT: 14.5s  aPTT: 28s  INR: 1.21  Plt: 411  Coagulopathy | No AV administered (no AV specific to *Hypnale spp.* exists)  *Progression of swelling & diminishing SpO_2_ | <24h  1 Fasciotomy  2 Wound closure  procedures | Discharge (14d): Survival |
| Senthilkumaran et al. (2023) (72)  India | 28  F  *Daboia russelii*  1h to local hospital, returned after 3d to different hospital, this presentation) | Ankle | Swelling (marked)  Ecchymosis | Clinical only | >72h | aPTT: 30.5s  INR: 1.45  Plt: 155  Coagulopathy | AV administered  1h: 100mL  Total AV: 100mL  pRBCs (2 units)  Methylprednisone IV  *Clinical deterioration three days after bite (this presentation) with swelling & ecchymosis | >72h  1 Fasciotomy | 6 months: No sequelae |
| Jatsho (2020) (73)  Bhutan | 11  M  Snake unknown | Index finger | Swelling (ext. to shoulder joint)  Pain (on finger movement & passive extension of arm muscles)  Tenderness  Palpable arterial pulsations in radial artery  Ecchymosis  Blistering  Erythema  Haemorrhage | Clinical only | Not reported | 20WBCT: positive  PT: prolonged  INR: prolonged  Coagulopathy | AV administered  24h: AV administered  Outside hospital:  Sucking out venom  Traditional remedies  Tourniquet  Inside hospital:  Antibiotics  Tetanus prophylaxis  Adrenaline  Antihistamines  Steroids  Oxygen | No operation performed | Discharge (7d): Limb salvage  Adverse antivenom reaction (cough, scratchy throat, urticarial rash & vomiting) |
| Bhattarai et al. (2025) (74)  Nepal | 39  F  *Trimeresurus spp.*  Initial care at local hospital, re-admission to different hospital >24h later, this presentation) | Third finger | Swelling (ext. to hand & forearm)  Pain  Pain on passive stretch  Paraesthesia  Hypoesthesia  Blistering | Clinical only | >24h | PT: 31.1s  INR: 2.7  Coagulopathy | AV administered  Total AV: 10 vials  Whole blood (2 units)  pRBCs (2 units)  FFP (2 units) | >24  1 Fasciotomy  1 Debridement  2 Wound closure  procedures | 1 month: No sequelae |
| Dahal et al. (2024) (75)  Nepal | 47  F  *Trimeresurus spp.* | Dorsum of hand | Swelling  Pain  Pain on passive stretch  Paraesthesia  Haemorrhage | Clinical only | 6h | PT: >3min  APTT: >5min  INR: >10  Coagulopathy | AV administered  <6h: 10 vials  Total AV: 10 vials  Whole blood (7 units; 2450ml)  pRBCs (4 units, 1000ml)  Platelet rich plasma (1 unit; 150m)  FFP (6 units; 1200ml)  Penicillin  Metronidazole  Cefuroxime Admission to ICU | 1 Fasciotomy &  dermotomy  ≥2 Wound closure  procedures | 3 years: No sequelae |
| Mariappan et al. (2024) (76)  India | 36  M  *Daboia russelii* | Leg | Not reported | Not reported | Not reported | Not reported | AV administered  Total AV: 20 vials | 1 Fasciotomy | Discharge: Limb salvage  Transverse myelitis |
| Mahadevaiah et al. (2024) (77)  India | 36  M  Snake unknown  30min | Foot | Swelling  Pain  Haemorrhage | Clinical only | 1d | Coagulation tests normal | AV administered  Total AV: 20 vials  Antibiotics  Plasmapheresis  Admission to ICU | 1 Fasciotomy | 14d: Deceased  Acute kidney injury, hemolytic uremic syndrome |
| Rathnayaka et al. (2024) (78)  Sri Lanka  Case 1 | 56  M  *Peltopelor trigonocephalus* | Foot | Swelling (severe)  No paraesthesia or paralysis  Reduced pulsations in dorsalis pedis artery | Clinical & doppler ultrasound  Reduced O_2_ saturation | Not reported | 20WBCT: positive  PT: >120s  INR: >10.5  Plt: 118  Coagulopathy | No AV administered (AV not available for *Peltopelor trigonocephalus*)  FFP (3 Packs) | 1 Fasciotomy  ≥1 Debridement (s)  1 Skin grafting & wound  closure procedure | Discharge (31d): Limb salvage |
| Rathnayaka et al. (2024) (78)  Sri Lanka  Case 2 | 27  M  *Peltopelor trigonocephalus*  Captive snake | Hand | Swelling (severe)  Pain  Ecchymosis  Lymphadenopathy (axillary & cervical lymph nodes) | Clinical & doppler ultrasound  Reduced O_2_ saturation in fingers | Not reported | Not reported | No AV administered (AV not available for *Peltopelor trigonocephalus*) | 1 Fasciotomy  1 Debridement, wound  closure & skin grafting | 2 months: Limb salvage with sequelae (deformity of left arm, numbness of fingers & inability to perform delicate tasks with fingers) |
| Srivastava et al. (2024) (79)  India | 12  F  Snake unknown | Foot | Swelling  Pain  Paraesthesia  Pulselessness  Poikilothermia  Pallor  Haemorrhage | ICP 34 mmHg | >48h | 20WBCT: negative | AV administered  Total AV: 30 vials | >48h  1 Fasciotomy | Discharge (5d): Limb salvage |
| Europe | | | | | | | | | |
| Vigasio et al. (1991) (80)  Italy | 4  F  *Vipera aspis*  ‘Immediately’ | Third finger | Swelling  Pain (strong)  Pain on passive stretch  Paraesthesia  Muscular paralysis  Cyanosis  Dark venous blood after pricking of thumb pulp | Absence of digital arterial pulsations during doppler examination | 1d 6h | Not reported | No AV administered  Elevation of affected extremity | >1d 6h  1 Fasciotomy (intrinsic  musculature of hand was  entirely oedematous &  haemorrhagic, abundant  outflow of interstitial  fluid & blood)  *Colour change from black  fingers to normal within  minutes | 30d: No sequelae  Coagulopathy developed in course of treatment |
| Al-Azzawi et al. (2016) (81)  Turkey | 1  M  Snake unknown  1d | Medial aspect of foot | Swelling (ext. to groin/ scrotum)  Pain  Pain on passive stretch  Tenderness  Intact arterial pulsations  CRT: 3-4s | Clinical only | 1d | PT: >60s aPTT: >180s INR: 5.10  Coagulopathy | AV administered  >2d: AV administered  Total AV: 25 vials  Herbal medicine  Antibiotics  Vitamin K  FFP  *Restoration of coagulation & significant reduction in swelling | No operation performed (operation first planned, but due to coagulopathy operation was delayed/ not performed) | 5 months: No sequelae |
| Anil et al. (2011) (82)  Turkey  Case 1 | 10  F  *Vipera xanthina*  1h | Ankle | Swelling (progressive)  Pain  Pain on passive stretch  Tenderness  Hypoesthesia  Impalpable arterial pulsations  Bruising up to knee  Pallor | Leg circumference 29.5cm compared to 25cm on healthy limb | 1d 12h | Coagulation tests  normal | AV administered  1h: 1 vial  *1 vial given in other hospital before referral; no additional antivenom given due to ‘late admission’  Mannitol  Antibiotics  *Complete disappearance of oedema after 5 days | No operation performed | Discharge (7d): No sequelae |
| Anil et al. (2011) (82)  Turkey  Case 2 | 12  F  *Vipera xanthina*  4h | Midfoot | Swelling (ext. to abdomen)  Pain  Pain on passive stretch  Tenderness  Paraesthesia  Hypoesthesia  Impalpable arterial pulsations  Pallor | Difference in leg circumference affected versus not affected 5.5cm at thigh & 6.0cm at calf | 3d | Coagulation tests  normal | AV administered  4h: 5 vials  Total AV: 5 vials  *Antivenom given in other hospital before referral; thereafter no additional antivenom given  Mannitol  Antibiotics  Tetanus vaccination  IV fluids | No operation performed | Discharge (7d): Limb salvage |
| Arif et al. (2014) (83)  Austria | 14  M  *Vipera* *berus*  ‘Shortly after bite’ | Proximal phalanx of index finger | Swelling (ext. to thorax)  Pain  Hypoesthesia  Skin discolouration | Clinical only | 2d | PT: 68% (of normal activity)  INR: 1.31  Coagulopathy | No AV administered  Antibiotics  Thrombosis prophylaxis Analgesics  Benzodiazepines  Local cooling  Immobilization of arm | 1 Fasciotomy  ≥1 Follow-up procedure (s) | Discharge: Limb salvage with sequelae (pain, sensory disturbances, restricted mobility of hand/limb) |
| Barani et al. (2021) (84)  France | 11  M  *Viperidae*  1h 30min | First web space of hand | Swelling (extensive)  Pain (VAS=9/10)  Paraesthesia  Posture: Hyperextension of MCP joints & flexion of IP joints in rest  Ecchymosis | Clinical only | **≤1d** | Not reported | AV administered  1h 30min: 4ml  Total AV: 4mL | <6h  1 Fasciotomy (visible  muscle necrosis in first  web space at site of the  bite, on pathological  exam necrotic muscle  cells with haemorrhagic  suffusion)  ≥1 Follow-up procedure (s) | 8 months: No sequelae |
| Cawrse et al. (2002) (85)  United Kingdom | 10  F  *Vipera berus* | Superior malleolus | Swelling (ext. to above knee)  Pain on passive stretch (minimal)  Tenderness  No neurovascular deficit  Erythema | ICP 30 mmHg in anterior & posterior muscle compartment, ΔP continuously >30 mmHg | 3d | ‘Slightly abnormal coagulation tests’ | AV administered  3d: AV administered  Immobilization  Application of venous compression bandages  Limb elevation  *ICP 20–23 mmHg in anterior & posterior compartment after 12h; ICP 7 mmHg in anterior & 14 mmHg in posterior compartment after 24h. After 3 days proximal extension of oedema & ecchymosis into thigh, increasing pain. | 3d  1 Fasciotomy  (haemorrhagic fat &  fascia around puncture  site & tense fascia in  thigh, muscle appeared  healthy)  1 Wound closure  procedure | Discharge: Limb salvage |
| Carvalho et al. (2021) (86)  Portugal | 11  M  *Viperidae*  2h | Dorsum of hand | Swelling (rapidly progressive, ext. to shoulder)  Pain (severe)  Pain on passive stretch  Tenderness  Palpable arterial pulsations  CRT: < 2s  Ecchymosis | Circumference of affected limb > than contralateral limb | 5h | Coagulation tests  normal  Plt: 22  Thrombocytopenia | AV administered  5h (in OR): 1 vial  Post-op: 1 vial  Total AV: 2 vials  Epinephrine  Dopamine  Clindamycin  Antibiotics  Tetanus vaccination  IV fluids  Platelets  FFP  Cryoprecipitate fibrinogen  *Upper limb became firm & tender, increased pain & pain on passive stretch | 5h  1 Fasciotomy (extensive  oedema in affected  compartments)  8 Procedures for  debridement & wound  closure | 1 month: Limb salvage with sequelae (limitation on wrist extension) |
| Pietrangiolillo et al. (2012) (87)  Italy | 22 months  M  Snake unknown  <4h | Proximal phalanx of second finger | Swelling (hard, ext. to arm)  Pain (strong)  Hypoesthesia  Muscular paralysis  Impalpable arterial pulsations  Cyanosis  Lymphangitis (ext. to axilla)  Haemorrhage from bite site | Clinical only | >6h | aPTT: 22s | AV administered  6h: AV administered  Antibiotics  Mannitol  Morphine  Glucocorticoids  Oxygen  IV fluids  *Worsening of local symptoms | <12h  1 Fasciotomy  1 Follow-up procedure  *Significant improvement of condition following fasciotomy | Discharge (11d): No sequelae |
| Faber et al. (2010) (88)  Italy | 44  M  *Bitis parviocula*  Captive snake  1h | Hand | Swelling  Pain  Hypoesthesia  Progressive circulatory impairment in hand  Reduced skin temperature at bite site  Bullous lesions | Clinical only | 5d | Coagulation tests  normal  Plt: 83  Thrombocytopenia | AV administered  18h: 2 vials  25h: 5 vials  Total AV: 7 vials | 5d  1 Fasciotomy | Discharge: Limb salvage with sequelae (contraction deformity, ‘claw-like’, in bitten finger) |
| Anil et al. (2010) (89)  Turkey | 7  F  *Vipera xanthina*  3h | Distal forearm | Swelling (ext. up to shoulder)  Pain  Pain on passive stretch  Tenderness  Hypoesthesia  Impalpable arterial pulsations  Fingertips not perfused  Bruising  Pallor | Clinical only | Not reported | PT: 26.4s  aPTT: 44s  INR: 2.44  Plt: 91  Coagulopathy  Thrombocytopenia | AV administered  4h: 10 vials  Total AV: 10 vials  *More AV was not available, authors intended to give more  Outside hospital:  Incision at bite site  Inside hospital:  Tetanus vaccination  Dopamine  Adrenaline  Antibiotics  IV fluids  Platelets  FFP | 1 Fasciotomy | Discharge (15d): No sequelae  Haemorrhage from surgical wound after operation |
| Grenc et al.  (2016) (90)  Slovenia | 37  M  *Crotalus adamanteus*  Captive snake  1h | Hand (interdigital fold between fourth & fifth fingers) | Swelling (ext. to above wrist)  Pain (intolerable)  Paraesthesia  Blisters  Laceration on the proximal IP joint of the third finger  Haemorrhage from fang marks  Hypotension | Clinical only | 6h | Coagulopathy | AV administered  1h: 8 vials  Total AV: 8 vials  *Despite intention to give more AV, none was available. Only after fasciotomy additional AV could be administered. | 6h  1 Fasciotomy | Discharge: Limb salvage |
| Fuchs et al. (2019) (91)  Switzerland | 45  M  *Dendroaspis viridis*  Captive snake  ‘Immediately’ | Left forearm | Pain  Paraesthesia  Erythema | ICP 38 mmHg in forearm | **≤**1d | Not reported | No AV administered (patient refused treatment with SAIMR polyvalent AV)  Outside hospital:  Patient immediately applied a tourniquet after bite  Inside hospital:  Antibiotics  Analgesia  IV fluids  Elevation of arm  *Increase of ICP after 3h to 52 mmHg | 1 d  1 Fasciotomy  1 Wound closure  procedure  *Decrease of swelling over post-op days 1 & 2 | 2.5 months: No sequelae |
| Evers et al. (2010) (92)  Denmark/ Germany (treatment) | 44  M  *Viperidae*  ‘Same day’ | Hand | Swelling (ext. to upper arm)  Pain (VAS=7/10)  Tenderness  Paraesthesia  Lymphangitis ext. to axilla | Clinical only | 1d | Not reported | AV administered  1d: AV administered  Analgesia  Bandage | 1d (fasciotomy performed before AV was available)  1 Fasciotomy (necrotic  muscle tissue,  haemorrhagic spots)  1 Wound closure  procedure  *Significant reduction of clinical signs of CS post-op | 1 year: No sequelae |
| Glatstein et al. (2019) (93)  Israel | 3  M *Echis coloratus*  1h 40min to initial hospital, 8h to referral centre (this presentation) | Dorsum of hand | Swelling  Pain (severe)  Haemorrhagic vesicles  Erythema | Clinical only | <8h | Coagulopathy  Platelets= 40  Thrombocytopenia | AV administered  8h: 5 vials  10h: 5 vials  Total AV: 10 vials  Ceftriaxone  Epinephrine  Ketamine  RBCs (5 units)  Platelets (10 units)  FFP (15 units)  Cryoprecipitate fibrinogen  IV fluids  Intubation | <8h (fasciotomy performed before AV given)  1 Fasciotomy  1 Wound closure  procedure | Discharge (14d): No sequelae  Profuse haemorrhage during & after operation culminating in haemorrhagic shock & DIC |
| Meyer-Rath (2003) (94)  Germany | 27  M  *Crotalus adamanteus*  Captive snake  8h | Little finger | Swelling (ext. to forearm)  Pain (severe)  Hypoesthesia (ext. from finger to ulnar forearm)  Ecchymosis  Blistering  Livid discolouration | Clinical only | 8–10h | PT: 71% (of normal activity)  aPTT: 51s  Plt: No thrombocytopenia  Coagulopathy | No AV administered (antivenom withheld due to fear of anaphylactic reaction & absence of systemic symptoms) | 10h  1 Fasciotomy  2 Wound closure  procedures, one  involving a debridement | Discharge (14d): Limb salvage with sequelae (dysesthesia/hypoesthesia on little finger) |
| Le Roux et al. (2021) (95)  France | *M*  *Crotalus atrox*  Captive snake  15min | Thenar eminence of hand | Swelling (ext. proximal to elbow)  Pain  Pain on mobilization  Hypoesthesia  Paralysis of arm (difficult to assess due to pain)  Chills  Ecchymosis  Pallor  Erythema | ICP >50 mmHg in thenar compartment, hypothenar & intermetacarpal areas & in anterior territory of the right forearm in operating room | 17h | Coagulation tests  normal | AV administered  3h: 3 vials  Day 1: 3 vials  Total AV: 6 vials  Corticosteroids Acetaminophen  Transfer to ICU | 17h  1 Fasciotomy (fluid  collection on the dorsal  area of the hand, but  preserved muscles) | 20d: Limb salvage with sequelae (incomplete mobility of fifth finger & still healing fascial wounds) |
| Rainer et al. (2010) (96)  Austria | 25  M  *Bitis arietans*  Captive snake  4h | Hand (between thumb & index finger) | Swelling (ext. proximal to elbow)  Pain (severe)  Patient able to move fingers & sensation preserved  Increased skin temperature  Erythema | Clinical only | Not reported | Coagulation tests  normal  Plt: 23  Thrombocytopenia | AV administered  4h 30min: 11 vials  Total AV: 11 vials  *All vials beyond expiration date but given regardless because of progressive swelling  Outside hospital:  Tourniquet  Inside hospital:  IV analgesia  Prednisolon  Tetanus vaccination  Antihistamine  Norepinephrine  Prophylactic antibiotics  IV fluids  pRBCs (2 units)  Hyperbaric O_2_-therapy (9 days) | No operation performed | Discharge (11d): No sequelae |
| Parrilla Parrilla et al. (2002) (97)  Spain | 9  F  Snake unknown | Anterior aspect of wrist | Swelling (ext. to arm)  Tightness of skin  Pain  Pain on mobilization of arm  Ecchymosis  Painful lymphadenopathy in axilla | Clinical only | >12h | Coagulation tests normal | No AV administered  Dexchlorpheniramine Metimazole  Prednisolone  Amoxicillin-clavulanate  Elevation of arm | No operation performed | Discharge (48h): No sequelae |
| Schnecker (1990) (98)  Germany | 25  M  *Crotalus atrox*  Captive snake (suicidal intention)  4–5h | Styloid process of the radius/forearm | Swelling (ext. to forearm, worsened upon release of tourniquet)  Paraesthesia  Black discolouration (where tourniquet was placed)  Ecchymosis  Blueish hue at bite site | Clinical only | Not reported | Consumptive coagulopathy | AV administered  Outside hospital:  Tourniquet (maintained for 3h)  Inside hospital:  Correction of coagulopathy & release of tourniquet | 1 Fasciotomy  1 SSG procedure  *Post-op improvement of perfusion, on post-op day 2 blueish hue disappeared. On day 7 after operation, opposition of thumb still impaired due to injury of median nerve branch but restored after three weeks | 3 weeks: No sequelae |
| Kuzbari et al. (1994) (99)  Austria | 31  M  *Vipera xanthina*  Captive snake (zoo)  1h 10min | Ring finger | Swelling (ext. to proximal extremity)  Pain  Pain on passive stretch (of fingers)  Sensory deficits  Paraesthesia  Palpable arterial pulsations in radial & ulnar arteries  CR: visible on nailbed of ring finger  Haemorrhagic blisters  Ecchymosis | Clinical only | 19h | Coagulation tests  normal | AV administered  40min: 30mL  Total AV: 30mL  Triamcinolone acetonide Splinting of the affected extremity  Opening of haemorrhagic blisters | 19h  1 Fasciotomy, including  carpal tunnel release (no  haemorrhage or necrosis  in muscle seen during  fasciotomy)  1 Wound closure  procedure  *Post-op quick reduction in pain, but swelling extended to ipsilateral thorax | 3 months: Limb salvage (near complete functional recovery) with sequelae (rupture of extensor tendon in distal interphalangeal joint section) |
| Tucker et al. (2005) (100)  United Kingdom | 42  M  *Vipera berus* | Ulnar border of thumb | Swelling (ext. up to middle third of upper arm)  Tenseness of skin  Pain (despite morphine)  Pain on mobilization  Tenderness (extreme)  Sensory deficit (tip of thumb)  Perfusion intact (tip of thumb) | ICP 24 mmHg in forearm, 37 mmHg in thumb adductor, 48 mmHg in thenar eminence & 59 mmHg thumb in OR | 14h | PT: 11.4s | No AV administered  IV fluids  *Increasingly severe pain | 14h  1 Fasciotomy  1 Wound closure  procedure  *ICP 33 mmHg in thumb &  thenar muscles | 5 months: No sequelae |
| Top et al. (2006) (101)  Netherlands | 26  M  *Atheris chlorechis*  Captive snake  ‘Immediately’ | Index finger | Swelling  Pain | Clinical only | Not reported | Plt: 128  Thrombocytopenia | AV administered  12h: 2 vials  In following 6h: 4 vials  Total AV: 6 vials (AV given after operation as not available initially)  Prednisolone  Antihistamines  FFP (during operation) | <7h  1 Fasciotomy  1 Additional fasciotomy  *Post-op after first fasciotomy, pain & swelling increased, progressing to the wrist, for which second fasciotomy initiated. | Discharge: Limb salvage with sequelae (small necrotic area that remained on distal phalanx)  Coagulopathy  Massive haemorrhage (approx. 5L in first 6h) following operation, haemorrhage from wound sites & insertion sites for four days, haematemesis & gingival haemorrhage |
| Tincu et al. (2017) (102)  Romania | 32  F  *Crotalus atrox*  Captive snake  45min | Forearm | Swelling (ext. to axilla)  Pain (severe)  Pain on mobilization of hand & fingers  Tenderness (axilla & epitrochlear lymph nodes)  Paraesthesia  Increased skin temperature  Lymphangitis (ext. to axilla)  Haemorrhage from bite site | Doppler ultrasonography: non-compressibility & microthrombosis of the brachial vein consistent with deep vein thrombosis | Not reported | PT: prolonged  aPTT: prolonged  Coagulopathy | No AV administered (AV was not available)  Piperacillin-tazobactam Linezolid  Metronidazole  Unfractionated heparin  Warfarin | 1 Fasciotomy  *On day 2 after operation, reduction of local tenderness & swelling. Local improvement achieved in next 4 days with progressive diminishment of local tenderness & swelling | Discharge: Limb salvage |
| Wagner et al. (1986) (103)  Switzerland | F  *Bitis arietans*  Captive snake  <3h | Dorsum of hand | Hypoesthesia  Anaesthesia  Limited movement of all fingers  Petechiae (shoulders & gums) | Clinical only | 3h | Severe coagulopathy with reduced coagulation factors  Plt= 4  Thrombocytopenia | AV administered  3h: 80 mL  Total AV: 80mL  Corticosteroids  Platelets (6 units)  *Improvement of coagulation profile after AV | 1 Fasciotomy (no local skin  or muscle necrosis)  1 Wound closure  procedure & skin  grafting | Discharge (10d): No sequelae |
| Styf (1985) (104)  Sweden | 45  F  *Vipera berus*  2d | Dorsum of foot | Swelling  Pain  Pain on palpation of anterior & lateral compartments  Pain in lower leg on passive stretch of toes  Pain on passive flexion of ankle joint & first metatarsal  Hypoesthesia  Discolouration of lateral malleolus | ICP 36 mmHg in anterior, 22 mmHg in lateral, 20 mmHg in deep posterior & 18 mmHg in superficial posterior compartment | 2d | Not reported | No AV administered  Flucloxacilline (for post-op wound infection)  Splinting | 2d  1 Fasciotomy of lower leg  (oedematous muscle in  anterior (here also  haemorrhage) &  posterior muscle  compartment, herniation  of nervus peroneus)  1 Fasciotomy of foot  1 Wound closure  procedure | 10 months: Limb salvage with sequelae (recovery of motor function, though loss of sensation in fourth & fifth toe on foot)  Wound infection of fasciotomy site 6 weeks after operation |
| Roed et al. (2009) (105)  Denmark  Case 1 | 7  M  *Vipera berus*  2d | Small toe | Swelling  Discolouration of the entire lower extremity | ICP higher than perfusion pressure in posterior compartment of lower leg | 2d | Not reported | AV administered  2d (before operation): AV administered | 2d  1 Fasciotomy (Impression  of elevated pressure in  muscle compartment,  but muscle appeared  vital)  1 Wound closure  procedure | Discharge: No sequelae |
| Roed et al. (2009) (105)  Denmark  Case 2 | 11  M  *Vipera berus*  15min | Thumb | Swelling  Discolouration (upper extremity ext. to shoulder & thorax) | Clinical only | 1d | Not reported | AV administered  1d (perioperative): AV administered  Urine alkalization (post-op) | 1d  1 Fasciotomy (muscles  were oedematous upon  opening of fascial  compartments)  1 Wound closure  procedure | Discharge: No sequelae  Rhabdomyolysis |
| Sennwald (1992) (106)  Switzerland | 34  M  *Naja nigricollis*  Captive snake  2h | Hand | Swelling (mild)  Pain (intense)  Pain on mobilization of fingers  Anaesthesia  Blistering  Reduced skin temperature  Vitality of skin at the bite site appeared compromised | Clinical only | 3d | Not reported | AV administered | 3d  1 Fasciotomy, carpal  tunnel release &  debridement  (discolouration of the  intrinsic muscles of the  hand & partial necrosis.  In forearm most muscle  appeared vital. Deep  extensor compartment  of forearm severely  damaged. Tendons of  long extensor & abductor  pollicis longus whitened  & peritendinous capillary  network looked defect.  Vitality of the skin  Compromised. Fat tissue  necrotic.)  1 Placement of a chinois  radialis flap  1 Corrective plastic  surgical procedure (6,5  years later)  *Post-op rapid reduction in swelling of the forearm and arm. Elevated pain persisted for four weeks. Extension of cutaneous necrosis to include the entire extensor apparatus. | 7 years: Limb salvage with sequelae (tenodesis of extensor tendons, difficulty forming a fist & force in hand 25% compared to healthy contralateral hand)  Persisting pain for 6 months  Proteinuria  Haemoglobinuria |
| Bozkurt et al. (2008) (107)  Turkey | *Viperidae* | Dorsum of hand | Swelling  Pain | Clinical only | Not reported | Not reported | No AV administered | 1 Fasciotomy  1 Wound closure  procedure  1 Full-thickness skin  grafting | Discharge: No sequelae |
| Zimmermann et al. (1981) (108)  Israel | 17  M  *Cerastes vipera*  Captive snake  6h | Middle finger | Swelling (ext. to antecubital fossa)  Pain  Haematoma  Blanching in affected finger  Small & tender axillary lymph node palpated on affected side  Haemorrhage (from wound) | Clinical only | 11h | Not reported | No AV administered  Analgesics  Tetanus vaccination  Elevation of affected extremity | 11h  1 Fasciotomy  *Swelling of affected limb was maximal 2–3 days after bite & then receded gradually | 6 months: Limb salvage with sequelae (hypoesthesia & fasciotomy scars (0.5cmx 0.5cm)) |
| Turchanyi et al. (2000) (109)  Hungary  Case 1 | 22/23 (unclear)  M  *Crotalus atrox*  Captive snake  45min | Index finger | Neurologic deficits  Sensory deficits  Motor deficits | Clinical only | 8h | PT: 50% (of normal activity)  Plt: thrombocytopenia  Coagulopathy | No AV administered  *AV was declined by patient for fear of adverse reaction  Plasmapheresis  FFP | 8h  1 Fasciotomy  1 Procedure for coverage  of skin defect | Discharge: No sequelae  Haemolysis  Acute kidney failure |
| Turchanyi et al. (2000) (109)  Hungary  Case 2 | 22/23 (unclear)  M  *Crotalus atrox*  Captive snake  45min | Proximal interphalangeal joint of thumb | Not reported | Clinical only | 7h | Coagulopathy | AV administered | 7h  1 Fasciotomy  ≥1 Skin-grafting & wound  closure procedure | Discharge: Limb salvage |
| Lau et al. (1985) (110)  United Kingdom | 12  M  *Vipera berus*  2h | Index finger | Swelling (hand, ext. to anterior chest wall)  Lymphangitis in upper arm | Clinical only | Not reported | Not reported | No AV administered  IV-fluids  Pethidine  Prochlorperazine  Systemic steroids  Elevation of limb  *Progression of swelling | 38h  1 Fasciotomy  1 Wound closure  procedure | 20 months: Limb salvage with sequelae (occasional aching of affected arm after prolonged use) |
| Arif et al. (2023) (111)  Italy | 6  F  *Vipera berus*  2h | Foot | Swelling (ext. to thigh)  Appearance of ischaemia | ICP (no details) | >7h | Not reported | AV administered  7h: 1 vial  Total AV: 1 vial  Intubation | >7h  1 Fasciotomy | Discharge: Limb salvage |
| Bernasconi et al. (2023) (111)  Italy  Case 2 | 38  F  *Vipera berus*  3h until initial presentation, then discharged, returned 48h later (this presentation) | Foot | Swelling (ext. to proximal thigh)  Pain (severe)  Large haematoma | Clinical only | >87h | Plt: normal | AV administered  Total AV: 1 vial  *Worsening of local symptoms | >87h  1 Fasciotomy | Discharge: Survival with ‘major’ sequelae (unspecified)  Local wound infection |
| Glatstein et al.  (2022) (112) | 8  M  *Echis coloratus* | Upper limb | Swelling | ICP (no details) | Not reported | Coagulopathy | AV administered  6h: 80mL | 1 Fasciotomy | Discharge: No sequelae |
| Aboud et al. (2025) (113)  Israel | 8  F  *Daboia palestinae*  30min | Lower leg | Swelling (ext. to thigh)  Pain  Pain on passive stretch  No neurological or vascular deficits  Blisters (haemorrhagic) | ICP 90 mmHg in anterior compartment & 70 mmHg in posterior compartment | 64,5h | PT=12.8s,  APTT=27s,  INR=1.1  Plt: 623 | AV administered  48min: 5 vials  Total AV: 5 vials  Analgesics  IV fluids | 64,5h  1 Fasciotomy (bulging of  muscles, no visible  ischaemia)  1 Wound closure  procedure | 2 months: No sequelae |
| Maffé et al. (2024) (114)  Italy | 33  M  *Vipera aspis francisciredi*  Captive snake | Hand/Wrist | Swelling  Paralysis  Absent arterial pulsations  Pallor  Erythema | ICP 20 mmHg | Not reported | Plt: 7  Thrombocytopenia | AV administered  Total AV: 1 vial  Norepinephrine  Piperacillin- tazobactam  Vancomycin Methylprednisolone Furosemide  Tetanus booster  Crystalloids  pRBCs (2 units)  Platelets (2 units)  Plasma (1 unit) | 1 Fasciotomy | 6 months: Limb salvage with sequelae (unspecified)  Venom-induced myocarditis |
| Sassoè-Pognetto et al. (2024) (115) | 6  F  *Viperidae*  1h 32min (case later transferred to different hospital, this presentation) | Ankle | Swelling  Pain  Lymph nodes in groin tender to palpation  Muscular paralysis  Absent arterial pulsations  Ecchymosis | ΔP <25mmHg in lateral leg compartment | 9h 40min | INR: 0.97  Plt: 303 | AV administered  8h 35min: 1 vial  Total AV: 1 vial  Paracetamol  Gentamicin  Betamethasone  Piperacillin-tazobactam Vancomycin  Fluconazole  Dexamethasone  Furosemide  Enoxaparin  Admission to the ICU | 1 Fasciotomy (‘Oedematous  imbibition’ in  subcutaneous tissue &  ischaemia of muscles)  1 Wound closure  procedure  *Progressive reduction in oedema & pain post-op | 3 years: No sequelae  Adverse reaction to AV (erythematous-papular skin rash; interpreted to potentially be serum sickness) |
| Eastern Mediterranean | | | | | | | | | |
| Bouziri et al. (2011) (116)  Tunisia | 11  M  *Cerastes cerastes*  2h | Index finger | Pain (intense)  Tenderness  Weak peripheral arterial pulsations  Cold extremity  Blue discoloration | Clinical only | <1d | Coagulation tests normal | No AV administered  Tetanus vaccination  Antibiotics  Adrenaline  IV-fluids  Intubation & mechanical ventilation | 1 Fasciotomy | Discharge (8d): No sequelae |
| Dhar (2015) (117)  Oman | 5  F  *Viperidae*  8h | Radial aspect of wrist | Swelling (significant, ext. to chest)  Pain on passive stretch  Tenderness  Impalpable arterial pulsations  Cold fingers  Bruising  Pallor | Clinical only | 14–15h | PT: 180s  aPTT: 180s INR: 10  Plt: 33  Coagulopathy  Thrombocytopenia | AV administered  8h: 10 vials  14h: 5 vials  Total AV: 15 vials  Outside hospital:  Proximal compression tourniquet  Inside hospital:  Tetanus vaccination  IV fluids  Blood transfusion  FFP  Wound cleaning | 14–15h  1 Fasciotomy ('muscles  with scattered patches of  duskiness’)  1 Wound closure  procedure  *1 week of post-operative pain & swelling, after which both reduced | 6 months: No sequelae |
| Hachimi et al. (2005) (118)  Morocco  Case 1 | 17  M  Snake unknown | Thumb/Hand | Swelling  Hypoesthesia (in hand)  Blisters  Cutaneous necrosis | Clinical only | 5d | Not reported | No AV administered  Analgesia  Antibiotics | 1 Fasciotomy  ≥ 1 Follow-up procedures,  including skin-grafting,  arthrodesis of joint &  drainage of  interphalangeal arthritis | Discharge: Limb salvage with sequelae (stiffness of fingers & wrist after operation)  Arthritis with staphylococcus infection in interphalangeal joint |
| Hachimi et al. (2005) (118)  Morocco  Case 2 | 18  M  Snake unknown  7d | Hand | Swelling  Pain  Ecchymosis (ext. to trunk) | Doppler of right extremity to confirm thrombosis | 7d | Not reported | No AV administered (authors suggest AV not effective seven days after envenomation)  Heparin (post-op) | 1 Fasciotomy | Discharge: No sequelae  Thrombosis  Respiratory distress  Pleuritis |
| Hamdi et al. (2010) (119)  Tunisia | 22  M  *Vipera lebetina*  1h | Thenar eminence/Hand | Swelling (ext. to forearm)  Tenseness (forearm)  Pain (severe)  Paraesthesia  No distal vascular deficit | ICP 49 mmHg in thenar eminence, 43 mmHg in volar forearm & 40 mmHg in dorsal forearm | <1d | Not reported | AV administered  1h: 5 vials  Total AV: 5 vials  IV Fluids | 1 Fasciotomy (muscles  appeared healthy)  1 Wound closure  procedure  1 Skin grafting procedure | 7 months: No sequelae |
| Al-Hashaykeh et al. (2011) (120)  United Arab Emirates | 12  M  *Cerastes* spp.  30min | Anteromedial aspect of lower leg | Swelling  Pain (VAS=5/10)  Tenderness  Diminished arterial pulsations  Shiny skin appearance  Haemorrhage from fang marks | Calf circumference of affected leg 37cm compared to 27cm on healthy side. Thigh circumference of affected leg 40cm compared to 34cm on healthy side | 24h | PT: >120 sec  PTT: >180 sec  Plt: 18  Coagulopathy  Thrombocytopenia | AV administered  30min: 5 vials  Total AV: 5 vials  Hydrocortisone  Tetanus vaccination  Fentanyl  Acetaminophen  Meropenem  Vancomycin  Epinephrine  pRBCs (355mL)  Platelets (7 units)  FFP (570mL)  Cryoprecipitate (10 units)  Vitamin K (5mg)  *Coagulation profile returned to normal. Within 12h of treatment circumference of affected vs non-affected leg normalized. | No operation performed (despite indication for fasciotomy, surgeons were concerned about haemorrhage considering coagulopathy & thrombocytopenia) | Discharge (1 week): No sequelae  Adverse reaction to AV |
| Kazemi et al. (2021) (121)  Iran  Case 1 | 15  M  *Macrovipera lebetina obtusa*  <1h | Hand palm | Swelling  Pain (severe)  Weakness  Itching around bite site | ICP >45 mmHg (compartment not reported) | Not reported | Not reported | AV administered  1h: 1 vial  12h: 5 vials  Total AV: 6 vials  Loratidine  Cefazoline (post-op)  *Progressive swelling & persisting pain | >12h  1 Fasciotomy  *Fasciotomy carried out for lack of responsiveness to AV, 2 days after operation localized swelling reduced | Discharge (13d): Limb salvage with sequelae  (image of fasciotomy site shows significant scarring, no details reported in text) |
| Kazemi et al. (2021) (121)  Iran  Case 2 | 36  M  *Macrovipera lebetina obtusa*  30min | Dorsum of left hand | Swelling  Pain  Tenderness  Increased skin temperature  Ecchymosis  Erythema | Clinical only | Not reported | Not reported | AV administered  Total AV: 5 vials  Hydrochlorthiazide  Loratidine  Blood transfusions  ICU admission  Debridement & cleansing of bite site | 1 Fasciotomy | Discharge (1 week): No sequelae |
| Kazemi et al. (2021) (121)  Iran  Case 3 | 30  M  *Macrovipera lebetina obtusa*  10h | Leg | Swelling (extensive)  Pain  Weakness  Ecchymosis  Blistering | Clinical only | Not reported | Not reported | AV administered  10h: 2 vials  Total AV: 2 vials  Opioids | 1 Fasciotomy  >1 Follow-up procedure  (patient returned to the  OR every 2–3 days for  debridement)  1 Above-knee amputation | Discharge: Sequelae (above-knee amputation) |
| Alsaman at al. (2023) (122)  Syria | 8  F  72h  Snake unknown | Leg | Swelling  Pain  Palpable pulsations  Blistering  Necrosis | Clinical only | >72h | INR: 2.7–3.0  Coagulopathy | AV administered  Total AV: Not specified (5 vials/2h)  Meropenem  Clavulanic acid  IV fluids  pRBCs  FFP | >72h  1 Fasciotomy  1 Debridement  1 Wound closure & skin  grafting procedure | 1 year: Limb salvage with functional disability (footdrop) |
| Navaeifar et al.  (2023) (123)  Iran | 3  M  14h  Snake unknown | Dorsum of hand | Swelling (severe, ext. to forearm)  Pain on passive stretch  Tenderness  Feeble distal arterial pulsations  CR: delayed  Cold fingers  Ecchymosis  Cyanosis  Pallor | Clinical only | Not reported | PT: 14s  aPTT: 35s  INR: 1.3  plt: 220  Coagulopathy | AV administered  Outside hospital:  First-aid in field: tying of ligature around limb, ice packs, incision at the bite site  Inside hospital  Local wound washing  IV fluids | 1 Fasciotomy (dusky  appearance of muscle  tissue)  1 Wound closure  procedure  *After 12h notable improvement | 3 months: No sequelae |
| Western Pacific | | | | | | | | | |
| Pin et al. (2018) (124)  China | 61  M  *Deinagkistrodon acutus*  8h | Ulnar side of forearm | Swelling (tense)  Pain (VAS=8/10)  Pain on passive stretch  Paraesthesia  Erythema | ICP 46 mmHg 9h after bite | Not reported | aPTT: 20.9s  INR: 0.93 | AV administered  9h: 8000 units  Total AV: 8000 units  IV Glucocorticoids infusion Mannitol  Furosemide  *Despite therapy progression of swelling, & severe pain (VAS=10/10) & low skin temperature. ICP 53 mmHg 12h after bite. | 12h  1 Fasciotomy (no bulging  muscle seen, but some  necrotic tissue)  1 Debridement  1 Aneurysm repair  1 Skin grafting procedure | Discharge (37d): No sequelae  Aneurysm of ulnar artery |
| Okamoto et al. (2017) (125)  Japan | 84  F  *Gloydius blomhoffii*  Initial care at other hospital,  2d until admission at referral hospital (this presentation) | Elbow | Swelling (expanding & diffuse)  Flexion contraction in the fingers  Ecchymosis (ext. up to trunk) | Clinical only | Not reported | PT: 59% (of normal activity)  INR:1.3 Plt: 34  Thrombocytopenia  DIC | AV administered  20h: First dose of AV  26h: Second dose of AV  IV fluids  Respiratory support Haemodialysis  Haemofiltration  *Development of ileus, bowel expansion, melena, abdominal free air & sigmoid necrosis. | 24h  1 Fasciotomy  ≥1 Abdominal procedures | 35d: Deceased due to bacterial peritonitis related to bowel necrosis  DIC  Acute renal failure  Hepatic failure  Rhabdomyolysis  Respiratory arrest  Ileus  Bacterial peritonitis  Sigmoid necrosis |
| Hsu et al. (1990) (126)  (Taiwan) | 1 year 10 months  F  *Trimeresurus mucrosquamatous*  Initial care at local hospital, 2h to referral hospital (this presentation) | Lower leg | Swelling (extreme, ext. to foot)  Tenseness  Mobilization of limb painful & limited  CR: absent  Blistering  Cyanosis (toes)  Mottling (lower leg) | Clinical only | Not reported | Coagulation tests normal | AV administered  Total AV: 1.5 vials  Crystalloids | 1 Fasciotomy (muscles of  lateral & anterior  compartment were  ischaemic & bulged  through the fasciotomy  incision. Fang marks  penetrated into the  muscle, ischaemic  change less noticeable in  the superficial & deep  posterior compartments)  ≥1 Wound closure  procedure | Discharge: No sequelae |
| Sugamata et al. (2011) (127)  Japan  Case 1 | 20  M  *Gloydius blomhoffii* | Dorsum of proximal index finger | Swelling (hand & index finger)  Pain (severe)  Hypoesthesia (index finger) | Clinical only | <3h | Coagulation tests normal | AV administered  Total AV: 6000 units  Hydrocortisone  Sodium succinate  Cepharanthin | 3h  1 Relaxation incisions  (both sides of index  finger)  1 Relaxation incisions  (dorsal hand, lateral  thenar compartment &  distal portion of flexor  retinaculum)  1 Wound closure  procedure  *Severe pain & numbness decreased on the second day post-op | 3 months: No sequelae |
| Sugamata et al. (2011) (127)  Japan  Case 2 | 11  M  *Gloydius blomhoffii* | Proximal crease of index finger | Swelling (severe)  Pain (severe) | Subcutaneous tissue pressure was 70 mmHg after first incisions were performed | Not reported | Not reported | AV administered  Total AV: 6000 units  Hydrocortisone  Sodium succinate  Cepharanthin  *Subcutaneous tissue pressure of 70 mmHg | 3h  1 Relaxation incision  (index finger)  1 Relaxation incision on  dorsum of the hand &  thenar compartment  1 Wound closure  procedure  *After initial incisions, the numbness of the index finger continued & subcutaneous tissue pressure measured 70 mmHg. After additional incisions, subcutaneous tissue pressures measured 18 mmHg | 4 months: No sequelae |
| Sugamata et al. (2011) (127)    Japan  Case 3 | 8  M  *Gloydius blomhoffii* | Dorsum of thumb | Swelling (notable)  Pain  Hypoesthesia  Erythema | Clinical only | <3h | Not reported | AV administered  Total AV: 6000 units  Methylprednisolone  Sodium succinate Cepharanthin  *Persisting numbness in thumb | 3h  1 Relaxation incision on  lateral side of the thumb  1 Relaxation incision  added on dorsal hand,  lateral thenar  compartment & partial  flexor retinaculum  1 Wound closure & full-  thickness skin grafting  *After initial incisions, hypoesthesia of thumb persisted. After additional incisions, numbness of finger & swelling of hand diminished. | 5 months: No sequelae |
| Singline et al. (2023) (128)  Australia | 39  M  *Pseudoechis australis* | Wrist | Swelling (severe)  Tenderness  Inability to move hand & fingers  Absent arterial pulsations in wrist | Clinical only | 20h | Not reported | No AV administered  Outside & inside hospital:  Pressure immobilization continued for 8h | 22h  1 Fasciotomy  1 SSG procedure | 31d: Limb salvage with sequelae (recovery of function, but loss of strength in affected limb) |
| Little (2023) (129)  Australia | 35  M  Snake unknown | Lateral epicondyle of elbow | Swelling  Pain  Pain on palpation & passive movement of fingers  Tenderness  Reduced sensation in distal limbs | ICP 150 mmHg in dorsal compartment & 74 mmHg in volar compartment | >9,5h | Not reported | Outside & inside hospital:  Pressure immobilization continued for 9,5h  Oxycodone  Fentanyl | >9,5h  1 Fasciotomy (dusky  appearance of muscle  tissue in dorsal  compartment)  ≥1 Debridement (s)  ≥1 SSG procedure (s) | Discharge (15d): Limb salvage |
| Matori et al. (2013) (130)  Japan | 66  F  *Protobothrops flavoviridis*  1h | Foot | Swelling  Pain (severe)  Pain on passive stretch  Erythema | ICP 70 mmHg in gastrocnemius muscle | 5h | APTT: 43.9s  INR: 1.10  Plt: 165  Coagulopathy | AV administered  3h: AV administered  Furosemide  IV fluids | 5h  1 Fasciotomy  2 SSG procedures | Discharge (63d):  No sequelae  Intra-operative haemorrhage due to the effect of hump-nosed viper venom |
| Kawamura et al. (2002) (131)  Japan | 63  M  *Agkistrodon blomhoffii* | Proximal phalanx of fifth digit | Swelling (progressive, ext. to shoulder)  Paralysis | ICP >41 mmHg (sustained elevation) | >20h | APTT: 28.7s  PT: 86.7%  Plt: 243  Coagulopathy | AV administered  >90h: AV administered  Admission to ICU  Mechanical ventilation  Wound cleansing | >20h  1 Fasciotomy | Discharge (54d): No sequelae  Respiratory paralysis due to neurotoxic envenoming |
| Cheng Chen et al. (2024) (132)  China | 51  M  Snake unknown  5h | Lower leg (calf) | Swelling  Pain  Muscular paralysis of foot extensor muscles  Reduced arterial pulsations in dorsalis pedis artery | Clinical only | Not reported | Plt: 5  Coagulation tests normal  Thrombocytopenia | AV administered  Antibiotics  Dexamethasone  Furosemide  Enoxaparin | ≥9d  1 Fasciotomy (necrotic  appearance of muscle in  anterior compartment)  1 Debridement | Discharge: Limb salvage with sequelae (limited dorsiflexion of the foot) |

**Abbreviations:** M, Male; F, Female; D, Day; h, Hour; min, Minutes; CS, Compartment syndrome; ext., extending (to); VAS, Visual Analog Scale (pain); MCP, Metacarpophalangeal joint; CR (T), Capillary refill time; MRI, Magnetic Resonance Imaging; CRT; ICP, Intracompartmental pressure; ΔP, Delta pressure, calculated as the diastolic blood pressure minus the intracompartmental pressure; 20WBCT, 20-minute whole blood clotting test; (A)PTT, Activated Partial Thromboplastin Time; PT, Prothrombin time; INR, International Normalized Ratio; Plt, Platelets; DIC, Disseminated intravascular coagulation; AV, Antivenom; Vit. K, Vitamin K; FFP, Fresh-Frozen Plasma; (p)RBC, Packed red blood cells; PCC, Prothrombin Complex Concentrate; IV fluids, Intravenous fluids; ICU, Intensive care unit; VAC, Vacuum-assisted wound closure; OR, Operating room; SSG, split-skin graft; post-op, post-operatively; CPK, Creatine Phosphokinase

^1^ ‘Captive snake’ refers to bites incurred from captive animals, including animals kept at zoos

^2^ When exact times were not reported, but a minimum or maximum time that elapsed between the bite and the event in question could be inferred, then this is

reported instead. If authors reported that an event occurred on a certain day of admission, e.g. ‘Day 1’, then this was denoted as ‘1d’

^3^ As measured at admission or pre-op, whichever was reported first. Author-reported coagulopathy/thrombocytopenia was noted. Where reference values were

unavailable, reference values from Bucaretchi et al. [39] were used for defining coagulopathy and thrombocytopenia: International normalized ratio (INR) <1.20,

Activated Partial Thromboplastin Time (APTT/PTT) <28s, Prothrombin time (PT) <14.3s, platelets (plt) 150–400 x 10^3^/mm^3^ or 150–400 x 10^9^/L

^4^ One vial of antivenom typically contains 10mL

^5^ If no exact time point of follow-up was reported, the day of hospital discharge (counting from hospital admission) is presented as the last verifiable moment of

patient assessment

^6^ If detailed clinical outcomes were not reported, but it could be inferred from the available information that no amputation had taken place, the outcome was

defined as ‘limb salvage’ only. If limb salvage could not be inferred, but death was not reported, the outcome was registered as ‘survival’.
