# Supplementary material for: Under pressure: Clinical management of venom-induced compartment syndrome in snakebite–A scoping review of the global literature
Source: PLoS Negl Trop Dis. 2026 Jul 31;20(7):e0014536. doi: 10.1371/journal.pntd.0014536 (PMC13427016; doi:10.1371/journal.pntd.0014536)
Supplement: S4 File — (DOCX) [file pntd.0014536.s004.docx]

**Supplementary Material S4: Animal studies investigating diagnostic and therapeutic approaches to snake venom-induced compartment syndrome**

| Author (Year)  Country | Objective, animal model & snake species of the venom used | Exposure (venom, route & site of injection), intervention group  (number of animals and protocol followed), control group (number of animals) | Key findings |
| --- | --- | --- | --- |
| Garfin et al. (1984) (133)  USA | Assess the effect of fasciotomy on tissue pressure & muscle necrosis  Dogs (n=11; 16 limbs)  *Crotalus viridis helleri* venom | Exposure:  IM/ SC injection of snake venom (3mg) into hind limbs  Intervention group:  Fasciectomy (n=6 limbs) followed by immediate primary closure of skin & intramuscular injection of venom  Control groups:  IM injection of venom without prior fasciectomy (n=10 limbs)  SC injection of venom without prior fasciectomy (n=6 limbs) | - SC venom injection did not result in markedly elevated ICP (mean 6 mmHg) or muscle necrosis after 24h - IM venom injection resulted in elevated ICP (mean 80 mmHg) after 24h in animals not treated with fasciectomy - ICP after 24h was lower (mean 20 mmHg) in animals treated with fasciectomy preceding the intramuscular injection of venom - Muscular necrosis was equally present in animals treated with fasciectomy & those not treated with fasciectomy |
| Garfin et al. (1985) (25)  USA | To determine whether ICP monitoring can help guide AV administration in envenomation of muscle compartments  Dogs (n=20; 31 limbs)  *Crotalus viridis helleri* venom | Exposure:  Venom (3mg) injection into anterolateral muscle compartment of hindlimb  Intervention group:  In 7 limbs, 1h after venom injection 4 vials of AV given (IV)  In 10 limbs, 1h after venom injection 8 vials of AV given (IV)  Control groups:  In 12 limbs injection of venom; no interventions  In 2 limbs injection of saline; no interventions | - In all groups, ICP $\geq$ 49 mmHg at the 2h measurement - The highest maximum ICP recorded during the 48h-lasting experiment was 70 mmHg (after 4h) in the group treated without AV - In the group treated with 4 vials of AV, ICP peaked after 2h & reached a maximum of 66 mmHg - In the group treated with 8 vials of AV, ICP peaked between 2–4h and reached a maximum of 49 mmHg |
| Bush et al. (2004) (134)  USA | Investigate effect of pressure immobilization on ICP, limb circumference & mortality  Pigs (n=20)  *Crotalus atrox* venom | Exposure:  Venom (20mg/kg) injection into tibialis anterior muscle of hind limb  Intervention group:  Pressure immobilization one minute after venom injection (n=10 pigs)  Control group:  No pressure immobilization after venom injection (n=9)  * 1 pig in control group  died | - Mean ICP 2h after pressure immobilization was 67 mmHg vs 24 mmHg without pressure immobilization - The mean limb circumference in at time of death in animals treated with pressure immobilization was larger as compared to untreated animals (14.3cm vs 19.1cm) - The median survival time was longer in the pressure immobilization group (191 min vs 155 min) - Pressure immobilization improves survival, but greatly increases ICP & therefore should not be used in bites by snakes with cytotoxic venom |
| Stewart et al. (1989) (135)  USA | To compare antivenom, surgical treatment (fasciotomy, irrigation, & debridement of nonviable muscle) & a combination of both with respect to ICP & preservation of limb function  Rabbits (n=44)  *Crotalus atrox* venom | Exposure:  Venom (3mg/kg) injection into left anterior compartment of hind limb  Intervention group (treatments initiated 30min after venom injection):  Untreated (n=10)  AV alone (2 vials; n=10)  AV plus surgery (fasciotomy, irrigation, debridement of nonviable muscle; n=10)  Surgery alone (n=10)  Control:  Injection with saline only (n=4) | - At 30min post-envenomation, mean ICP for all envenomed animals had increased from 3.2 mmHg (baseline) to 30.2 mmHg & there was no significant difference in ICP between the different groups - Animals treated only with surgery showed extensive oedema and haemorrhage in venom-injected limb, similar appearance as in untreated group - Animals treated only with AV showed no decrease in wet and dry muscle mass (in comparison to control limbs), while the other groups did - Animals that received AV had better subjectively assessed muscle function than those that did not (i.e. surgery alone/ untreated), higher measured muscle tension at 9 weeks, greater resistance to muscle fatigue & as the only group a 100% survival rate |
| Tanen et al. (2003) (136)  USA | Investigate effect of AV (Crofab) on perfusion pressure (mean arterial pressure minus the compartment pressure) following IM venom injection  Pigs (n=20)  *Crotalus atrox* venom | Exposure:  Venom (6mg/kg)  injection into tibialis anterior muscle  Intervention group:  AV (IV; CroFab, 8 vials) 1h after venom injection (n=10)  Control:  Saline (IV) 1h after venom injection (n=10)  *Four of the control animals died before reaching the study endpoint. Direct comparison of MAPs may be exaggerated in favour of the control group, as only animals with better MAP survived | - 1h after venom injection, mean perfusion pressures decreased by 41% from 54.1 mmHg to 31.7 mmHg in all venom-injected animals, signifying reduced limb perfusion - Animals receiving antivenom had a consistently higher perfusion pressure (57% larger area under the curve) in the 1–8h time interval following venom injection, indicating improved limb perfusion in the antivenom group as compared to control animals - No deaths occurred in the AV group, whereas four deaths occurred in the control group |
| Tanen et al. (2004) (26)  USA | To compare the effect of fasciotomy, with or without AV on myonecrosis following IM injection of venom  Pigs (n=20)  *Crotalus atrox* venom | Exposure:  Venom (6mg/kg)  injection into tibialis anterior muscle of both hindlimbs (i.e. 12mg/kg/animal). Fasciotomies performed on right hind leg in all animals immediately after venom injection  Intervention group:  AV infusion (8 vials, CroFab, IV) 1h after venom injection (n=10)  Control:  Saline infusion (IV) 1h after venom injection (n=10)  *Four animals in the control group died before reaching the study endpoint. | - Significantly lower compartment pressures in limbs subjected to fasciotomy vs limbs not subjected to fasciotomy - Significantly more myonecrosis 8h after venom injection in limbs that underwent fasciotomy than in limbs that did not (14.5% vs 2.5%) - In limbs not subjected to fasciotomy, no difference was found in myonecrosis at 8h between animals receiving saline or AV (10% vs 10%) |
| Garfin et al. (1979) (137)  USA | To study the effect of surgical decompression on muscle necrosis in IM & SC envenoming  Dogs (n=unclear; 22 limbs)  Crotalus viridis helleri venom | Exposure:  IM/SC venom injection (3mg) into hind limbs  Intervention group:  Fasciectomy (n=6 limbs) performed prior to IM venom injection  Comparison group:  SC venom injection (n=6 limbs)  IM venom injection (n=10 limbs) | - Equal increase in limb circumference regardless of injection depth - 2h after envenomation mean pressures in SC envenomed limbs were: SC 5.40 mmHg & ICP 7.0 mmHg - 2h after envenomation mean pressures in IM envenomed limbs without fasciectomy were: SC pressure 4.0 mmHg & ICP 83.75 mmHg - 2h after envenomation mean pressures in IM envenomed limbs with fasciectomy were: ICP 19.60 mmHg - Histologically, equal degree of necrosis, inflammation & haemorrhage observed in IM envenomed limbs, regardless of prior fasciectomy. In limbs with SC venom injection only, normal appearance of muscle compartments |
| Grace et al. (1980) (138)  USA | To compare AV, glucocorticoids & fasciotomy with respect to local complications following rattlesnake envenomation  Rabbits (n=96)  *Crotalus atrox* venom | Exposure:  IM venom injection 2mg/kg into hind limbs  Interventions (start 30min after venom injection):  AV (10mL, IV)  Steroids (dexamethasone sodium phosphate 5mg/kg/24h)  AV (10mL, IV) + steroids (same as group 2)  Fasciotomy  Control:  Venom injection without treatment | - Severe muscular necrosis observed in all animals, regardless of treatment received - Swelling decreased significantly in all treated animals as compared to untreated animals - Significant decrease in soft tissue haemorrhage in antivenin and antivenin + steroids group compared to untreated control animals - No reduction in swelling and soft tissue haemorrhage seen in animals treated only with steroids - No notable increase in compartmental pressure in neither treatment nor control groups |

**Abbreviations:** AV, Antivenom; IM, Intramuscular; IV, Intravenous; SC, Subcutaneous; SE, Standard error
