# Supplementary material for: Under pressure: Clinical management of venom-induced compartment syndrome in snakebite–A scoping review of the global literature
Source: PLoS Negl Trop Dis. 2026 Jul 31;20(7):e0014536. doi: 10.1371/journal.pntd.0014536 (PMC13427016; doi:10.1371/journal.pntd.0014536)
Supplement: S5 File — (DOCX) [file pntd.0014536.s005.docx]

**Supplementary Material S5: General literature on snake venom-induced compartment syndrome**

| Author, country, year | Objective | Type of study, sampling population, snake species | Number of total patients & patients with VICS (% of total patients) | Diagnostic methods for VICS (*diagnostic criteria used for VICS) | Treatment/ Interventions | Findings, Clinical outcomes/complications |
| --- | --- | --- | --- | --- | --- | --- |
| Darracq et al. (2015)  (27)  USA | Comparison of clinical characteristics of snakebite patients for whom fasciotomy was discussed but not performed, & patients for whom fasciotomy was performed | Retrospective observational study  All patients bitten by rattlesnakes included in poison system database in state of California & for whom fasciotomy was discussed or performed  *Crotalus spp.* | Total:  Fasciotomy discussed in 105/443 patients, of which 102 patients included in analysis  With VICS:  Not reported | ICP obtained in 2 (7%) patients treated with fasciotomy (70 & 36 mmHg) & in  six patients not treated with fasciotomy (recorded as 60, 20, 18, <15 mmHg, 'ok' and 'normal'). | Antivenom:  Median of 4,5 vials prior to fasciotomy & median 13,5 vials post-operatively; in non-surgical patients median of 18 vials  Fasciotomy:  28 patients   - 8 Finger - 7 Hand - 7 Leg - 4 Arm - 2 Foot | Findings:  In 74 cases (17%) fasciotomy was discussed, but not performed  Poison centre contacted in 24/28 (86%) cases in which fasciotomy was performed. In four cases fasciotomy was performed despite poison centre staff recommending not to.  No significant difference between patients treated with fasciotomy & those treated non-surgically in terms of age, gender, bite site, snakebite severity score & quantity of antivenom administered  Patients treated with fasciotomy hospitalized for median of 5 days vs 3 days for patients treated without fasciotomy |
| Downey et al. (1991)  (146)  USA | Review demographic features, treatment & clinical outcomes of patients with rattlesnake bites | Retrospective observational study  Hospitalized patients (one hospital) with a history of snakebite  *Crotalus spp.* (n=29), not reported/unknown snake species (n=7) | Total:  36 patients  With VICS:  25 patients (69%) | Clinical signs (tenseness & painful stretch, n=11; Dysesthesia, n=8; absent pulsation, n=1)  ICP (30–55 mmHg) obtained in three patients in interosseous compartment of hand (n=2) and leg (n=1)  *ICP $\geq$ 30mmHg used as cut-off to define VICS (reference test) | Antivenom:  22 (61%) patients in entire cohort  Fasciotomy:  25 (100%) patients   - 9 Hand & forearm - 8 Foot - 8 Leg - 7 Finger | Findings:  In two patients intraoperative finding of necrosis following diagnosis of CS  Complications:  Transfusion of RBCs  for anaemia post-op (n=4, all FASC)  Wound infections at fasciotomy site (n=2) |
| Ho et al. (2021)  (20)  Taiwan | To assess the utility of point-of-care ultrasound (POCUS) in distinguishing IM/SC tissue swelling & arterial blood flow in snake-bitten limbs | Prospective observational study  All snakebite patients presenting to one hospital  *P. mucrosquama- tus, T. stejnegeri, B. multicinctus, Colubridae spp.* | Total:  27 patients, of which 17 involved bites by *Crotalinae*  With VICS:  None | POCUS (in patients bitten by Crotalinae*,* n=17) to assess depth of tissue swelling & assessment of arterial blood flow using diastolic retrograde arterial flow with the unaffected limb as reference | Antivenom:  All patients received AV (1–22 vials/patient)  Fasciotomy:  None | Findings:  Swelling had a cobblestone appearance in subcutaneous tissue & did not extend beyond the fascia in any of the patients  No diastolic retrograde arterial flow detected in any of the affected limbs  No sequelae at two-week follow-up |
| Chih-Po Hsu et al.  (2015)  (147)  Taiwan | Identify predictors of VICS & study the clinical prognosis of patients with VICS | Retrospective observational study  All snakebite patients  aged 18–80 years presenting <72h following the bite to one hospital  *Trimeresurus stejnegeri* (n=1)  *Trimeresurus mucrosquamatus* (n=2)  *Naja atra* (n=1)  Unknown snake species (n=5) | Total:  136 patients  With VICS:  9 patients (7%) | Clinical signs (Six P’s: pain, pallor, paraesthesia, paralysis, poikilothermia, pulselessness)  * >2 P’s present and/or ICP ΔP$\leq$ 20 mmHg | Antivenom:  All ‘symptomatic’ patients received AV  Fasciotomy:  9 patients (100%)   - 4 Hand - 2 Forearm - 1 Foot - 1 Leg | Findings:  Ischaemia/necrosis in six patients with VICS  Complications:  Below-knee amputation (n=1, NO FASC)  Mean days of hospitalization in VICS patients was 17.2 days vs 3.3 days in non-VICS patients |
| Kim et al.  (2019)  (148)  Korea | Report experience with ICP measurements & fasciotomy in patients with snakebite envenoming | Retrospective observational study  All patients bitten by venomous snakes presenting to one hospital <48h after snakebite envenoming  Snake species not identified | Total:  59 patients  Suspected VICS:  33 patients (56%)  With confirmed VICS:  17 patients (29%) | ICP measured every 4h until symptoms resolved or 2 consecutive readings >40 mmHg (indication to perform fasciotomy)  *ICP >40 mmHg & no decrease in pressure 4h after initiating AV treatment | Antivenom:  Administered to all patients with VICS  Fasciotomy:  17 (100%)  patients   - 7 Finger - 4 Hand - 3 Foot - 2 Forearm - 1 Toe | Findings:  ICP measured in 33 patients in whom CS was suspected clinically of whom 17 (52%) diagnosed with VICS based on ICP measurement (mean ICP 49.6 mmHg, range 37–88 mmHg) |
| El Koraichi et al. (2011)  (149)  Morocco | Describe clinical characteristics of VICS in children envenomed by vipers in a setting where antivenom is unavailable | Retrospective observational study  Children admitted to paediatric intensive care unit & diagnosed with VICS  Snake species not identified (suspected *Viperidae*) | Total:  7 patients  With VICS:  7 patients | Clinical signs (pain, pain on passive stretch, oedema & hypoesthesias; no motor deficits) | Antivenom:  Not administered (Unavailable)  Fasciotomy:  7 patients (100%)   - 5 in lower extremity - 2 in upper extremity | Complications:  Transfusions of frozen plasma, platelets and RBCs (n=6, all FASC)  Wound infections (n=2, both FASC)  Death due to multi-organ failure (n=1, FASC) |
| Mars et al. (1994)  (150)  South Africa | Investigate pulse oximetry (SpO_2_) as an indicator of tissue perfusion & ICP in compartment syndrome | Prospective observational study  Convenience sample of patients (snakebite, n=4; no snakebite, n=10) referred for suspicion of elevated ICP  Snake species not identified | Total:  4 patients  With VICS:  4 patients | Case 1: Arm, ICP 64 mmHg  Case 2: Leg, ICP 28 mmHg  Case 3: Arm, ICP 54 mmHg  Case 4: Leg, ICP 24 mmHg | Antivenom:  Not reported  Fasciotomy:  1 patient (25%) | Findings:  SpO_2_ is not a reliable indicator of increased ICP  Complications:  Necrotic muscle found intra-operatively (n=1, FASC) |
| Mars et al. (1991)  (139)  South Africa | Direct ICP measurement performed prospectively in children in whom VICS suspected | Prospective & retrospective observational study  All patients admitted for snakebite to one hospital  Snake species not identified | Total:  Retrospective: 206 (all snakebite patients)  Prospective: 9 (only patients with suspicion of VICS)  With VICS:  7 patients (3%, retrospective)  9 patients (prospective) | Retrospective:  ICP measured in all patients treated with fasciotomy  Prospective:  ICP measured in 9 children with suspected VICS. Eight had ICP range of 8–18 mmHg & in all cases ΔP > 30 mmHg. In one patient ICP 64 & 67 mmHg | Antivenom:  Not reported  Fasciotomy:  5 patients (31%)   - 4 (all in leg) in   retrospective  study   - 1 (in leg) in   prospective  study | Findings:  Pressure measurements can identify patients with elevated pressures that are subcritical & reduce rate of surgical interventions  Complications:  Above-knee amputations (n=2, FASC)  Below-knee amputation (n=1, FASC)  Death (n=1, FASC)  No sequalae in the eight children managed conservatively in prospective study |
| Tuerkmen et al. (2016)  (140)  Turkey | Evaluation of an algorithmic approach to decision-making in the diagnosis and treatment of suspected VICS | Prospective observational study  All snakebite patients presenting to the emergency department of one hospital  Snake species not identified | Total:  97 patients  Suspected VICS:  40  Confirmed VICS:  3 patients (3%) | ICP  Doppler ultrasonography (failure to obtain pulse used to diagnose VICS)  Measurement of limb circumference (comparison to contralateral limb)  Pulse oximetry  *ICP >55 mmHg in three repeated measurements in 2-hour intervals | Antivenom:  84 patients (87%) received AV  Fasciotomy:  3 patients (100%)  Non-surgical treatment:  Mannitol 20% administered to all patients with suspected VICS | Findings:  Full recovery in 37 patients with suspected VICS managed conservatively with AV & mannitol. Serial evaluation of patients with CS-like symptoms using ICP monitoring can potentially reduce the number of fasciotomies performed.  Complications:  Skin grafting (n=1, FASC) |
| Wood et al. (2016)  (141)  South Africa | Assess diagnostic utility of ultrasound in the diagnosis of VICS | Prospective observational study  Convenience sample of patients presenting to one hospital with swelling following snakebite to the upper or lower limb  Snake species not identified | Total:  42 patients  With VICS:  1 patient (2%) | Thickness of subcutaneous & underlying muscle compartment in the envenomed limb measured using ultrasound and compared to the tissue thickness of respective compartment in contralateral limb (reference). The expansion coefficient for each tissue compartment was calculated from the two different limbs. | Antivenom:  18 patients  Fasciotomy:  1 patient (100%) | Findings:  Mean expansion coefficient was 2.0 (95% CI 1.7-2.3) in subcutaneous tissue & 1.06 (CI 1.0-1.1) in muscle tissue. The subcutaneous expansion coefficient differed between anatomic locations (lowest in foot, highest in leg). The patient with confirmed VICS (diagnostic method unclear) had the highest observed muscle expansion coefficient in the group (1.4, signifying 40% increased diameter of muscle compartment in bitten extremity compared to non-bitten extremity). This patient underwent fasciotomy, during which muscular necrosis observed. No significant correlation seen between muscle expansion coefficient & use of AV or use of debridement. |
| Hsieh et al. (2017)  (17)  Taiwan | To identify factors contributing to complications & the associated outcomes of snakebite in Taiwan | Retrospective observational study  All patients presenting to one hospital with a history of snakebite  *Trimeresurus stejnegeri*  (n=46)  *Naja atra*  (n=19)  *Trimeresurus mucrosquamatus*  (n=11)  *Bungarus multicinctus*  (n=6)  *Deinagkistrodon acutus*  (n=1)  *Crotalus spp.* (n=1)  Snake species not identified (n=59) | Total:  148 patients  With VICS:  31 patients (21%) | Not reported | Antivenom:  72% of all patients received AV  Fasciotomy:  Unclear | Findings:  Factors associated with CS: Bites on hand (OR 3.249 SE 0.521, P= 0.024), |
| Otero et al. (2002)  (10)  Colombia | To review clinical & epidemiological factors & complications of snakebite patients | Retrospective observational study  Children & adults presenting (transferred) to one hospital  *Bothrops spp.*  *Porthidium spp.*  *Bothriechis spp.* | Total:  39 patients  With VICS:  3 patients (8%) | ICP (measured repeatedly 1–2h after start of mannitol)  Three patients  had initial ICP of 37, 40 & 73 mmHg  *ICP >30 mmHg in children or >45 mmHg in adults | Antivenom:  31 patients received AV  Fasciotomy:  None  Non-surgical treatment:  IV infusion of mannitol (1-2g/kg) over 30-60min | Findings:  Decrease in ICP to 10 & 21 mmHg 1–2h after start of mannitol infusion (n=2)  Complications:  Volkmann’s ischaemic contracture, amputation & coagulopathy during initial treatment phase (n=1) |
| Garfin et al. (1979)  (142)  USA | Discuss concepts in the treatment of rattlesnake bites & VICS | Case series  Convenience sample of snakebite cases treated  *Crotalus spp.* | Total:  6 patients  With VICS:  None | ICP & SC tissue pressure measurement  *ICP >30 mmHg | Antivenom:  All 6 patients received AV  Fasciotomy:  None | Findings:  SC pressure ranged between 5–40 mmHg & IM pressure between 10-20 mmHg in all patients. In two patients, SC pressure > IM pressure. Clinically, dysesthesia, areas of anaesthesia, reduced motor function & pain on passive stretch recorded in patients, despite ICP readings being below threshold for CS. Clinical symptoms are not reliable indicators for the diagnosis of VICS.  Complications:  Normal findings in 5 patients on follow-up. Residual sensory deficit in 1 patient. |
| Essafti et al. (2022)  (143)  Morocco | Compare the outcomes of treatment in children before & after introduction of a snakebite treatment protocol & AV | Retrospective observational study  Children aged <15 years admitted to emergency department of one hospital  *Echis leucogaster* (n=4)  *Vipera latastei* (n=6)  *Cerastes cerastes* (n=2)  Snake species not identified (n=63) | Total:  75 patients  With VICS:  31 patients (41%) | Clinical signs (painful extensive oedema, paraesthesia, paralysis, pallor & pulselessness)  Measurement of limb diameter  Pulse oximetry  Non-invasive infrared spectroscopy | Antivenom:  39/75 patients received AV  Fasciotomy:  25 patients (81%) | Findings:  In the absence of antivenom, 47% (17/36) of patients were treated with fasciotomy vs 20% (8/39) when AV was available. |
| Spyres et al. (2023)  (14)  United States of America | Describe presentation & management of cases with suspected VICS following *Crotalidae* envenomation | Retrospective observational study  Cases of snakebite reported by toxicologists to the North American Snakebite Registry  *Crotalus spp.* (n=15)  *Agkistrodon* spp. (n=6)  Snake species not identified (n=1) | Total:  1604 patients  With VICS:  22 patients (1.4%) | Clinical signs  ICP measured in 5 patients, in four of whom ICP was elevated (>20 mmHg, range 24–80 mmHg) | Antivenom:  22/22 patients (100%)  Fasciotomy:  9 patients (41%)  Upper extremity (n=6)  Lower extremity (n=3) | Findings:  All patients with proven elevated ICP (>20 mmHg) were treated with fasciotomy; in a single patient with confirmed low ICP (8 mmHg) no fasciotomy was performed.  ICP measurement obtained in 5/22 patients (23%) with suspected VICS & in 44% of patients treated with fasciotomy. |
| Toffano et al. (2023)  (144)  Brazil | Present epidemiological, clinical & outcome data of patients (aged >18 years) with VICS | Prospective observational study  Snakebite patients aged >18 years admitted to the emergency department of one hospital  *Bothrops* spp.  *Crotalus* spp. | Total:  54 patients  With VICS:  7 patients (13%) | Clinical signs | Antivenom:  All patients undergoing fasciotomy received AV  Fasciotomy:  7 patients | Findings:  Mean time between snakebite & AV treatment in CS patients was 9.5h (range 5–20h)  Time from admission to fasciotomy 1–48h (range)  Outcomes/Complications:  Bacterial infection (n=7 patients, FASC)  Acute kidney injury (n=4 patients, FASC)  No long-term sequelae in any of the patients with VICS. |
| Xian et al. (2024)  (145)  China | Investigation of the relationship between limb circumferences and compartment pressures in bitten extremities of snakebite patients | Prospective observational study  All patients aged >18 years with limb swelling following snakebite & presenting to one hospital | Total:  115 patients  With VICS:  19 patients (17%) | *ICP >40 mmHg used as cut-off to define VICS (reference test)  For VICS group: n=6 in upper limb and n=13 in lower limb. For non-CS group n=27 in upper limb and n=69 in lower limb  Limb circumference (healthy limb as reference) | Antivenom:  At least one patient (not further reported)  Fasciotomy:  2 patients (11%; in the two patients that underwent fasciotomy, ICP was 65 and 60 mmHg) | Findings:  Circumference difference of $\geq$2.8cm had a sensitivity of 76.9% and specificity of 66.7% for VICS (ICP>40 mmHg).  Consistently higher circumference values in bitten limb in VICS group compared to no-VICS group.  Of the patients treated without fasciotomy, five had their ICP re-measured one day after the bite and in all, ICP was lower than before (<30 mmHg) |

**Abbreviations:** VICS, Venom-Induced Compartment Syndrome; ICP, Intracompartmental pressure; IM, Intramuscular; SC, Subcutaneous; POCUS, Point-of-Care Ultrasound; FASC, treated with fasciotomy; NO FASC, treated without fasciotomy; CS, Compartment syndrome; AV, Antivenom; ΔP, Delta pressure, calculated as the diastolic blood pressure minus the intracompartmental pressure
